# Electric Field Assisted Self-Healing of Open Circuits with Conductive Particle-Insulating Fluid Dispersions: Optimizing Dispersion Concentration

**DOI:** 10.1038/s41598-019-55801-8

**Published:** 2019-12-23

**Authors:** Virendra Parab, Oppili Prasad, Sreelal Pillai, Sanjiv Sambandan

**Affiliations:** 1 Department of Instrumentation and Applied Physics, Indian Institute of Science, Bangalore, 560012 India; 2 Centre for Nanoscience and Engineering, Indian Institute of Science, Instrumentation and Applied Physics, Bangalore, 560012 India; 3 Vikram Sarabai Space Centre, Indian Space Research Organisation, Trivandrum, India; 4 Department of Engineering, University of Cambridge, Cambridge, CB3 0FZ United Kingdom

**Keywords:** Electrical and electronic engineering, Condensed-matter physics, Engineering, Physics

## Abstract

Open circuit faults in electronic systems are a common failure mechanism, particularly in large area electronic systems such as display and image sensor arrays, flexible electronics and wearable electronics. To address this problem several methods to self heal open faults in real time have been investigated. One approach of interest to this work is the electric field assisted self-healing (eFASH) of open faults. eFASH uses a low concentration dispersion of conductive particles in an insulating fluid that is packaged over the interconnect. The electric field appearing in the open fault in a current carrying interconnect polarizes the conductive particles and chains them up to create a heal. This work studies the impact of dispersion concentration on the heal time, heal impedance and cross-talk when eFASH is used for self-healing. Theoretical predictions are supported by experimental evidence and an optimum dispersion concentration for effective self-healing is identified.

## Introduction

Open circuit faults in the interconnects of electronic systems occur due to several mechanisms such as current spikes during electrostatic discharge events, electro-migration, mechanical and thermal stress and environmental or chemical degradation. This problem is more significant in emerging technologies such as flexible and wearable electronic systems where there is a larger possibility for faults to occur during system operation^1,2^.

To address this problem, some of the passive approaches used to improve the tolerance of interconnects to mechanical stress related faults involve the tailoring of interconnect geometry^3–11^ and the use of stretchable conductive materials to build the interconnect^6,12–25^.

Active approaches involve the automatic real time repair (’self-healing’) of the open circuit fault in the interconnect. There have been several techniques investigated to achieve self-healing. The most common approach to self-healing interconnects is via the use of conductive inks embedded into the interconnect either as free flowing droplets or as encapsulated micro-spheres that break and spill the conductive liquid during fracture^26–31^. The ink could either be in elemental form (eg. Ga, In) or be a colloidal conductive ink. Healing is achieved by the mere flow of the liquid metal into the open gap. Other techniques such as the use of electrically conductive polymers or gels^32–34^ active transmission line modulation^35^, surface energy driven mechanisms^36^ have also been investigated.

**Figure 1:**
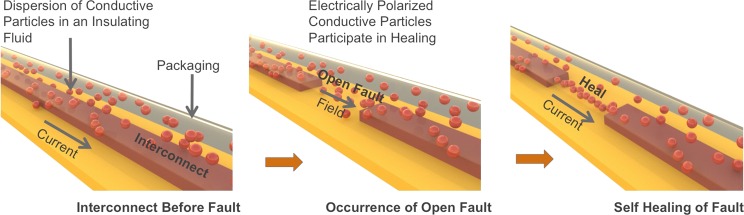
Electric field assisted self-healing (eFASH).

Yet another self-healing technique that is of interest to this work is the electric field assisted self-healing (eFASH)^37–42,43^. eFASH uses a low concentration dispersion (i.e. much lower than the percolation threshold) of electrically conductive particles in an insulating fluid. This dispersion is isolated and contained over the interconnects, for eg. by packaging it in conduits or vesicles with insulating walls that run alongside the interconnects (see Fig. 1). Upon the occurrence of an open circuit fault in a current carrying interconnect, the field appearing across the open gap polarizes the conductive particles in the dispersion. The polarized particles experience dipole-dipole attractive forces and eventually chain up to create a bridge across the gap thereby healing the fault. This mechanism turns on and shuts off by itself without external interference. The occurrence of the fault creates the electric field that triggers the mechanism. After the repair of the fault that results in a low resistance path across the open gap, the electric field across the gap disappears thereby stopping the mechanism.

Therefore, compared to self-healing using liquid metals or conductive inks, self-healing by eFASH is achieved not by the mere flow of the dispersion, but by the particles actively rushing to the site of the fault and locally increasing their concentration (on demand) to form a bridge across the gap. eFASH is an attractive approach to self-healing for several reasons. Firstly, as the dispersion is inherently insulating an accidental leak of the dispersion is less likely to cause a short circuit or cross-talk. This relaxes the constraints on the packaging techniques. Secondly, the eFASH technique does not require a change in the manner of fabrication of the interconnect. The dispersion can be added over the interconnects after they are patterned. The self-healing ability is therefore an add-on feature. Thirdly, eFASH achieves self-healing irrespective of the cause of the open failure - be it mechanical fracture, burnout due to electrostatic discharge, chemical corrosion etc. Finally, eFASH requires the use of relatively commonly available materials (eg. copper particles in silicone oil). In addition to these advantages, self healed interconnects using eFASH have been shown to permit mechanical stretching and compression without loss of functionality^37^. This makes eFASH an attractive prospect for flexible electronics.

There are three major performance parameters for self-healing. The first is the time taken to heal, *τ*_*h*_. The second is the steady state or final impedance of the heal, *R*_*h*∞_. The third is the cross-impedance between adjacent interconnects, *Z*_*c*_, which determines cross-talk and places a limit on the bandwidth of the circuit when the ability to self heal is incorporated. Since eFASH uses a sparse dispersion, the effectiveness of self-healing as defined by the performance parameters is sensitive to the dispersion concentration. A low dispersion concentration avoids accidental short circuits and reduces cross-talk between adjacent interconnects in case of packaging failures. However, a very low dispersion concentration results in a very large time to heal or no healing whatsoever. What then is the optimum dispersion concentration?

In this work we consider the impact of varying the dispersion concentration, i.e. from low (from an insulating ink) to high (towards a conductive ink), on the effectiveness of self-healing by the eFASH technique. We identify the theoretical dependence of each of the performance parameters on the dispersion concentration and then find an optimum dispersion concentration that permits most effective healing. Experiments and models are used to support the findings.

## Results and Discussions

### Situation under study: definitions

#### Interconnects

To develop analytical models for *τ*_*h*_ and *R*_*h*∞_ we consider a current carrying interconnect with an external series resistance *R*_*l*_ driven by a dc voltage *V*_*dc*_ experiencing an open circuit failure resulting in a gap of width *s*. For all experiments discussed in this work, *s* = 200 *μ*m, *V*_*dc*_ = 80 V, *R*_*l*_ = 4.4 kΩ.

To study the cross-impedance, *Z*_*c*_, we consider two adjacent interconnects separated by a distance *d*. Both interconnects have the dispersion packaged and isolated over them. One of the interconnects is driven by the impedance analyzer with an ac voltage of angular frequency *ω*. The frequency is swept to obtain the cross-impedance spectrum.

#### Properties of the dispersion

The dispersion used for self-healing consists of conductive particles homogeneously dispersed in an insulating fluid. While this study is not limited to any particular material or methods for the preparation of the dispersion, it is preferable that the fluid have a very low conductivity, an ability to withstand high electric fields, have a high boiling point and a viscosity that is large enough to discourage particle clustering and yet not too large to slow down the heal operation. The particles must have high conductivity and their geometry defines their polarizability and the viscous drag they experience while moving through the fluid. The particles are defined to have conductivity *σ*_*p*_, permittivity *ε*_*p*_, effective radius *r*_*p*_ and mass density *μ*_*p*_. The fluid is defined to have permittivity *ε*_*f*_, conductivity *σ*_*f*_ and viscosity *η*_*f*_. For experiments discussed in this work, all dispersions were prepared in silicone oil having *η*_*f*_ = 0.29 Ns/m^2^ and *ε*_*f*_ = 22.1 × 10^−12^ F/m.

The dispersion concentration is the key parameter for this study. The dispersion concentration in weight/volume is defined as Φ = (4*π*/3)*r*_*p*_^3^*μ*_*p*_*n* with *n* being the number of particles dispersed per unit volume of the fluid. We define the dimensionless ratio *φ* = Φ/*μ*_*p*_ as the normalized dispersion concentration to aid comparison between different types of particles. Thus, $n=\phi /((4\pi /3){r}_{p}^{3})$. When *φ* = 0, the dispersion is an insulating fluid. When *φ* = 1, the dispersion would be no different than a pure solid composed of the material of the particles. Therefore 0 ≤ *φ* ≤ 1. As the dispersion concentration is varied from low (from an insulating ink) to high (towards a conductive ink), *φ* varies from 0 to 1. In practice, the lower limit is defined by the minimum concentration of particles needed for successful self-healing whereas the maximum limit is defined by the percolation threshold beyond which the dispersion behaves like a conductive ink. The values of *φ* of interest to this work lies well within these limits. Table 1 defines the list of variables used in this work.

**Table 1:** List of variables.

Variable	Parameter Definition (Unit)	Value used in Study
*s*	length of open fault (m)	2 × 10^−4^
*d*	Distance between adjacent interconnects (m)	2 × 10^−3^
*R*_*l*_	external resistance (Ω)	4.4 × 10^3^
*V*_*dc*_	dc voltage driving the interconnect (V)	80
*ω*	frequency of ac component of voltage (Hz)	175 × 10^3^ to 15 × 10^6^
*ε*_*f*_	permittivity of the fluid (F/m)	2.21 × 10^−11^
*σ*_*p*_	conductivity of the particle (S/m)	5.9 × 10^7^ (copper)
*r*_*p*_	effective radius of the particle (m)	5 × 10^−6^
*μ*_*p*_	mass density of the particle (kg/m^3^)	8960 (copper)
*η*_*f*_	viscosity of the fluid (Ns/m^2^)	0.29
*n*	no. of particles/volume in dispersion (1 /m^3^)	
Φ	weight/volume of particles in dispersion (kg/m^3^)	
*φ*	normalized concentration, *φ* = Φ/*μ*_*p*_	
*x*	inter particle distance in dispersion (m)	
*P*	magnitude of the dipole moment (Cm)	
*τ*_*h*−*f*_	time taken to form the bridge (s)	
*τ*_*h*−*ox*_	time taken to breakdown surface oxide (s)	
*τ*_*h*−*s*_	time take to sinter the bridge (s)	
*τ*_*h*_	total heal time, *τ*_*h*_ = *τ*_*h*−*f*_ + *τ*_*h*−*ox*_ + *τ*_*h*−*s*_ (s)	
*R*_*h*∞_	resistance of a single sintered bridge (Ω)	
*Z*_*c*_	impedance between adjacent interconnects (S)	

### The mechanism of self-healing

**Figure 2:**
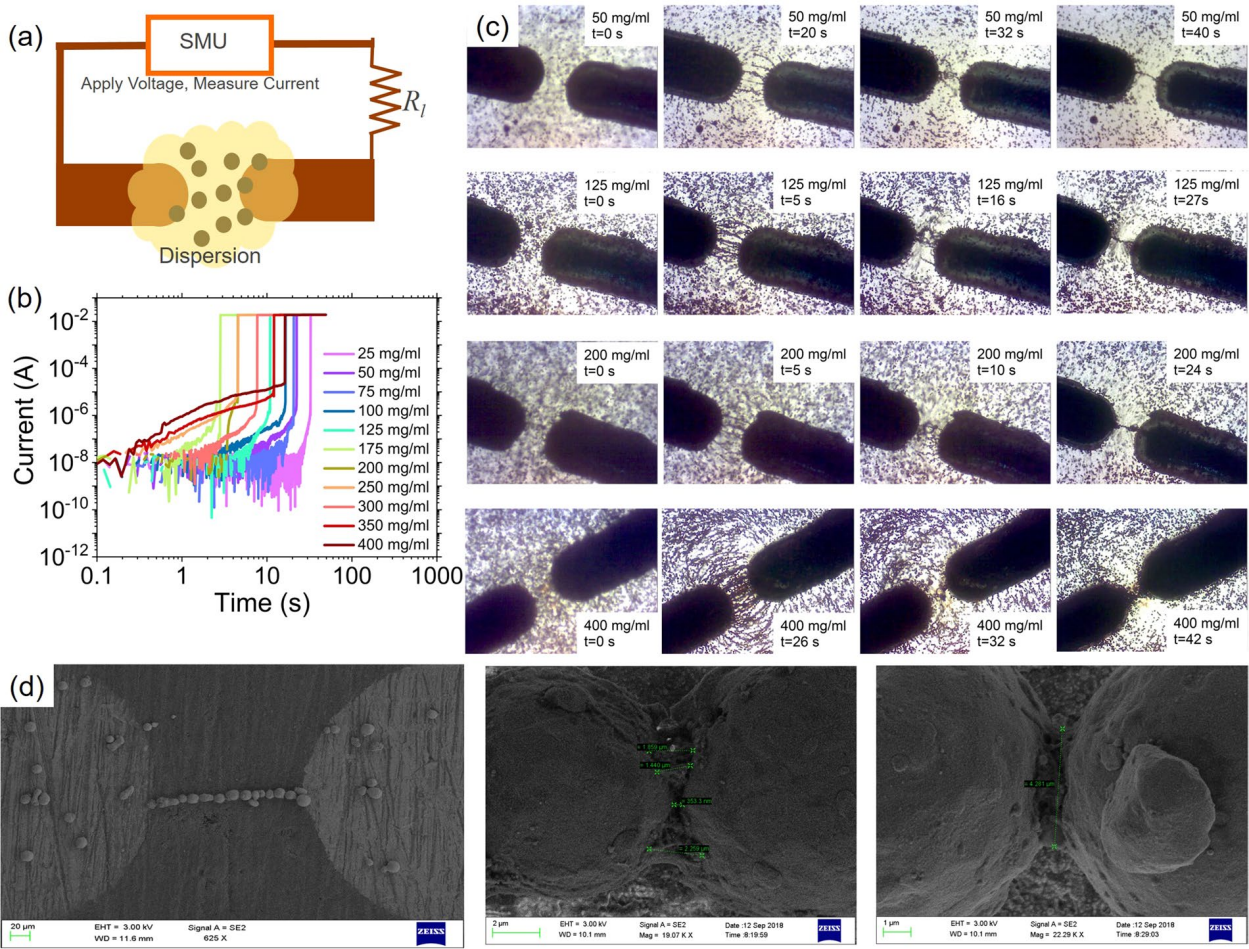
The mechanism of electric field assisted self-healing (eFASH). (**a**) Experimental setup. For these experiments, *R*_*l*_ = 4.4 kΩ. The dispersion used was dispersion of copper particles, radius *r*_*p*_ = 5 *μ*m in silicone oil, viscosity *η*_*f*_ = 0.29 Ns/m^2^. (**b**) Current versus time during self-healing for different dispersion concentrations. (See Section S1 Supplementary Material) (**c**) Photographs taken during the healing process for different dispersion concentrations. (**d**) SEM image of the sintered heal. The region of the bridge was first rinsed with iso propyl alchohol to remove any oil before obtaining the SEM image.

The experimental setup to demonstrate self-healing is shown in Fig. 2a. The two electrodes emulated the two ends of the open interconnect. The resistor of impedance, *R*_*l*_, represented the external impedance in series with the interconnect in a circuit. For this demonstration, a dispersion of copper particles (5 *μ*m radius) in silicone oil with varying concentrations was used and *R*_*l*_ = 4.4 kΩ. The use of the external resistance is critical for good emulation of the mechanism since it allows the voltage across the open gap to adapt and vary with time (despite a constant external voltage being applied) based depending on the time varying resistance across the gap during the healing process.

Figure 2b shows the plot of current versus time for different dispersion concentrations during the healing process. From Fig. 2b, we see that there are three regimes in the current versus time plot that define healing with copper dispersions. Initially, the current is very low and shows almost no variation with time. Then, the current begins to gradually increase with time before shooting up rapidly and stabilizing to a steady state. Figure 2c illustrates the sequence of events as captured from experimental videos for different dispersion concentrations. It was observed that upon the application of the electric field, the particles first chained up to form multiple parallel chains across the fault. However, despite the formation of a bridge (or multiple bridges), the current was not immediately restored. Instead the current climbed gradually. Finally, the bridges dissipate leaving a single bridge in place.

Based on these observations and best correlation of the video evidence with the current variation in time, we speculate that the process of self-healing occurs through four stages - polarization leading to a dipole moment in the particles, dipole-dipole attraction and formation of bridges, the breakdown of surface oxides or insulation films and finally sintering and completion of the heal (Fig. 2d).

#### Stage 1: Polarization and induced dipole moment in the particles

If an open fault resulting in an open gap of effective length *s* were to occur in a current carrying interconnect driven by a dc voltage *V*_*dc*_, an electric field *ξ* ≈ *V*_*dc*_/*s* would appear across this gap. This results in the polarization of the conductive particles of the dispersion in the vicinity of the gap i.e., in the volume space (2*π*/3)(*s*/2)^3^. The polarized particles have a dipole moment *P* that scales as $P \sim {r}_{p}^{3}{\varepsilon }_{f}\xi$. This moment of the occurrence of the fault is represented by the very low currents at close to time *t* = 0 in Fig. 2b and the first column of photographs (across different concentrations) in Fig. 2c.

#### Stage 2: Dipole-dipole attraction and the formation of bridges

The polarized particles now attract other polarized particles lying in cone of attraction via dipole-dipole forces that scale as ~*P*^2^/(*ε*_*f*_*x*^4^) with *x* being the centre-to-centre distance between two polarized particles at any time *t*. Upon experiencing this force, the particles initially accelerate and rapidly reach a velocity determined by the viscous drag. For particles with an effective spherical radius, *r*_*p*_, Stoke’s law defines the drag to be ~*η*_*f*_*r*_*p*_(*dx*/*dt*). Driven by dipole-dipole attraction, the particles of the dispersion present in the gap chain up to form an electrostatically held bridge across the gap. This bridge is stable. If a particle were to dislocate and leave the bridge, it would create a new open gap. The high electric field in this gap would once again polarize a nearby particle and bring it in to complete the bridge.

It is expected that after the occurrence of the fault, multiple bridges form across the gap. The local volume space of interest has ~(2*π*/3)(*s*/2)^3^*n* = (*φ*/2)(*s*/2*r*_*p*_)^3^ polarized particles. If all of these particles participate in the formation of bridges, ~(*φ*/2)(*s*/2*r*_*p*_)^2^ bridges form across the gap. The formation of bridges is represented by the second and third column (across different concentrations) of images in Fig. 2c.

#### Stage 3: Breakdown of surface insulation film in particles

Despite the formation of a bridge, the current through the interconnect does not immediately shoot up. We speculate this to be the result of the presence of insulating films on the surface of the particles eg. metal particles typically have surface oxides. The presence of oxide is confirmed by X-ray Photoelectron Spectroscopy measurements on copper particles (Supplementary Material Section S4 and Section S5). These insulating films exist at the interfaces of the particles constituting the electrostatically held bridge. Thus, despite the presence of a bridge of conductive particles across the gap, the resistance of the bridge remains high therefore restricting current flow through the interconnect. The high impedance of the bridge also results in a larger voltage drop across the gap thereby resulting in a larger field across the insulating film at the particle-particle interface. With time, the insulating film breaks down and a larger current begins to flow through the bridge. This slow increase in current is seen in Fig. 2b and is particularly prominent at high dispersion concentrations.

#### Stage 4: Sintering

Once the insulating film between the particles of the electrostatically held bridge has broken down at the particle-particle interface, a significant current flows through each of the multiple bridges. Due to small variations, eg. length of the bridge, degree of oxide breakdown at the particle-particle interface etc., the resistance of each bridge varies and the bridge with the least resistance transports the highest current. With time, the current causes Joule heating in the bridge and the bridge carrying the highest current heats the quickest. In the case of certain materials, eg. copper particles, the heating leads to the sintering of the particles. Sintering causes a considerable drop in the resistance of the bridge. Therefore there exists an intrinsic positive feedback mechanism during sintering wherein the bridge having the least resistance to begin with heats up quickest resulting a further decrease in resistance. Eventually, the bridge that sinters first creates a low resistance path (a practical short circuit) across the gap. The short circuit results in the loss of a voltage drop across the gap and therefore the loss of polarization in the particles that are not yet a part of a sintered bridge. This along with strong convective currents in the vicinity of the high temperature sintered bridge cause the loose particles to dissipate away. Eventually there exists one low resistance bridge of resistance *R*_*h*∞_ and the current shoots up as seen in Fig. 2b. At this instance, the open fault is considered healed. This event is seen in the final column (across difference concentrations) of photographs in Fig. 2c. Figure 2d shows the SEM of the sintered heal and the particle-particle binding. While sintering is observed in metallic particles eg. copper, it is absent in many other conductive particles eg. graphite. Since sintering results in very low impedance permanent heals, it is preferable to use particles that sinter.

### Performance parameters for self-healing interconnects

#### Heal time, *τ*_*h*_

**Figure 3:**
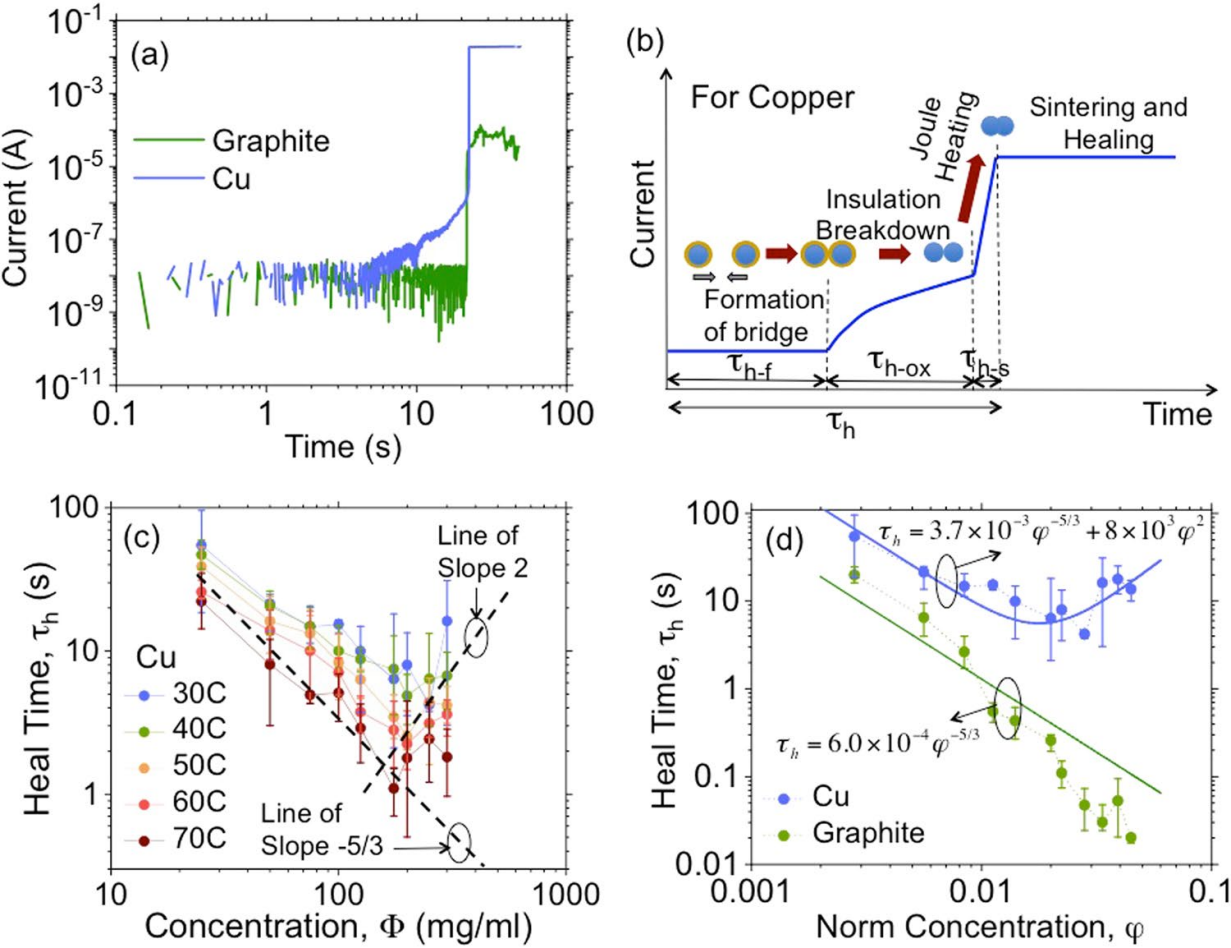
Modeling heal time, *τ*_*h*_. (**a**) Comparison of healing with dispersions using copper (oxide film and sintering expected) and graphite (no oxide and no sintering expected). (**b**) Cartoon illustration of healing with copper dispersions indicating the mechanism. (**c**) Heal time versus dispersion concentration in weight/volume of copper dispersions at different temperatures (Data sets for copper dispersions in Section S1, Figs. S1 to S47 of Supplementary Material). Lines of slope −5/3 and 2 are also sketched. (**d**) Comparison of heal time for copper and graphite dispersions at 30 C (Data sets for graphite dispersions in Section S2, Figs. S48 to S79 of Supplementary Material). The models based on Eq. 1 are plotted in solid lines.

This dynamics is strongly dependent on whether the particles sinter or not. Figure 3a shows self-healing using dispersions of copper and graphite (see Methods) in silicone oil with the concentration at *φ* = 0.005. Copper particles show all regimes of the stages discussed earlier. In the case of graphite, we neither expect any oxide film nor sintering and therefore healing simply occurs after Stage 2 but with a much weaker current carrying capacity. Therefore, in the case of graphite as seen in Fig. 3a we see the clear absence of the steady increase in current before the abrupt increase. Since sintering offers the advantage of a permanent heal and much lower heal resistance, our primary interest is in copper dispersions with graphite dispersions used as a counter example. The dynamics and sequence of events during self-healing via eFASH with copper dispersions is illustrated via the cartoon shown in Fig. 3b.

The total time taken for self-healing is the sum of three components with *τ*_*h*_ = *τ*_*h*−*f*_ + *τ*_*h*−*ox*_ + *τ*_*h*−*s*_. The first component, *τ*_*h*−*f*_, is the time taken for the formation of bridges of electrostatically held particles across the open gap (Stage 1 and Stage 2). The second component, *τ*_*h*−*ox*_ is the time taken to breakdown any surface oxides or insulating surface films at the interfaces of the particles constituting the bridge and establish a significant current flow (Stage 3). The third component, *τ*_*h*−*s*_ is the time taken for Joule heating in the bridge to sinter (if possible) the particles in the bridge (Stage 4). After time *τ*_*h*_ the open fault is healed with the heal having a low resistance.

To estimate *τ*_*h*−*f*_, we first estimate the time for two polarized particles to move through the fluid and come in contact. The time constant for two particles to attract each other and come in contact is obtained from equating the driving force of dipole-dipole attraction to the viscous drag. This time constant multiplied by a factor of *s*/2*r*_*p*_ (the number of particles in a single bridge across the gap) defines *τ*_*h*−*f*_. It can be shown that *τ*_*h*−*f*_ ∝ (*η*_*f*_/*ε*_*f*_)(*s*/*r*_*p*_)(*s*/*V*_*dc*_)^2^*φ*^−5/3^.

To estimate *τ*_*h*−*ox*_, we need to estimate the time to breakdown any surface oxides or insulating films at the particle-particle interface of the electrostatically held bridges. As the insulating film breaks down, the resistance of the bridge begins to decrease. Let this time dependent resistance of a single bridge be *R*_*h*_(*t*). Since there appear ~(*φ*/2)(*s*/2*r*_*p*_)^2^ parallel bridges at the end of Stage 2, the voltage drop across the gap after considering the effective parallel combination of resistances due to all bridges is *V*_*h*_(*t*) ~ *V*_*dc*_*R*_*h*_(*t*)/*(R*_*h*_(*t*) + *R*_*l*_(*φ*/2)(*s*/2*r*_*p*_)^2^). The voltage across the oxide between adjacent particles is therefore ~*V*_*h*_(*t*)(2*r*_*p*_/*s*). This voltage in turn determines the field in the oxide film sandwiched between two adjacent particles of the bridge. A reasonably good prediction of *τ*_*h*−*ox*_ is given by the Lloyd’s model^44^. The model estimates the time to breakdown of a dielectric to be inversely proportional to the product of the probability of defect creation and the current density. The current density depends on the mechanism of charge transport through the oxide and can only be determined by further experiments. Assuming the transport is limited by the oxide film and not the electron emission from the metal particle, the charge transport mechanism could be ohmic (at low fields), space charge limited, Poole-Frenkel like or hopping limited. For the purpose of this study, we subsume the product of the current density and the defect creation probability into a power law dependence on the electric field across the insulating film such that ln(*τ*_*h*−*ox*_) ∝ ln((2*r*_*p*_/*s*)*V*_*h*_(*t*)) with the constant of proportionality determined empirically. Clearly this constant of proportionality would be <0 since *τ*_*h*−*ox*_ is expected to decrease for increasing voltage across the oxide. Initially, i.e. just after the formation of the bridges at *t* = *τ*_*h*−*f*_, *R*_*h*_(*t*) is large, *V*_*h*_(*t*) is large and the field through the oxide is large. As the oxide at some of the particle interfaces begins to break down, *R*_*h*_(*t*) begins to decrease thereby reducing *V*_*h*_(*t*) and the field. This results in the breakdown process slowing down and is seen as a slower but steady increase in current in Fig. 2b. Therefore the break down time would be largely dominated by events occurring when *R*_*h*_(*t*) is ‘small’. To quantify ‘small’, it is noted that when the parallel resistance of all bridges becomes <*R*_*l*_, most of *V*_*dc*_ is dropped across *R*_*l*_ with *V*_*h*_(*t*) becoming small. Therefore, the breakdown process slows down when *R*_*h*_(*t*) < *R*_*l*_(*s*/2*r*_*p*_)^2^(*φ*/2). Therefore, to approximately estimate *τ*_*h*−*ox*_, we redefine *V*_*h*_(*t*) = *V*_*dc*_*R*_*h*_(*t*)/(*R*_*h*_(*t*) + *R*_*l*_(*φ*/2)(*s*/2*r*_*p*_)^2^) ∝ *V*_*dc*_(*r*_*p*_/*s*)^2^/*φ*. In essence this implies that a larger *φ* resulting in a larger number of parallel bridges implies a lower *V*_*h*_(*t*) and therefore a larger breakdown time.

To estimate *τ*_*h*−*s*_, we consider the time for the bridge to sinter. After time *τ*_*h*−*f*_ + *τ*_*h*−*ox*_, the oxide coating between the particles of the bridge break down considerably and a significant current begins to flow through the bridge. This current causes Joule heating and *τ*_*h* = *s*_ would be obtained by taking into account the rate of heat creation, the thermal mass of the bridge, the heat dissipation mechanisms and the temperature required for sintering. However, since the thermal mass of the bridge is low (product of mass and specific heat ≈1e − 11 J/K) and since the fluid does not significantly aid heat transport (thermal conductivity ≈0.15 W/mK), *τ*_*h*−*s*_ is negligible.

Therefore, the total heal time can be written in a general manner as,

1 $$\begin{array}{ccc}{\tau }_{h} & = & {\tau }_{h-f}+{\tau }_{h-ox}+{\tau }_{h-s}\approx {\tau }_{h-f}+{\tau }_{h-ox}\\  & \approx  & {k}_{1}\frac{{\eta }_{f}}{{\varepsilon }_{f}}\frac{s}{{r}_{p}}\frac{{s}^{2}}{{V}_{dc}^{2}}{\phi }^{{k}_{2}}+{k}_{3}{(\frac{\phi {(s/{r}_{p})}^{3}}{{V}_{dc}})}^{{k}_{4}}\end{array}$$

where *k*_1_ > 0, *k*_2_ < 0, *k*_3_ > 0 and *k*_4_ > 0 are constant coefficients with *k*_3_ having units sV^*k4*^.

Figure 3c shows the dependence of *τ*_*h*_ as a function of dispersion concentration, Φ, using dispersions of copper particles (*r*_*p*_ = 5 *μ*m) in silicone oil (See Methods). Healing was not observed for Φ < 25 mg/ml. The dispersion became extremely viscous and non homogeneous for Φ > 400 mg/ml. Therefore values of Φ within these limits was used for experiments.

Figure 3d shows the variation of *τ*_*h*_ for dispersions of copper and graphite in silicone oil (See Methods) with the dispersion maintained at 30C. With copper dispersions, both the presence of a surface oxide and sintering are expected and the model for *τ*_*h*_ is expected to be well described by Eq. 1. For copper dispersions, the heal time for low *φ* is dominated by the bridge formation time and therefore *τ*_*h*_ ≈ *τ*_*h*−*f*_ decreases with increasing *φ*. On the other hand, the heal time for high *φ* is dominated by the oxide breakdown time and therefore *τ*_*h*_ ≈ *τ*_*h*−*ox*_ increases with increasing *φ*. For intermediate values of *φ*, *τ*_*h*_ = *τ*_*h*−*f*_ + *τ*_*h*−*ox*_ shows a minima for some 0 < *φ* < 1. Therefore, for copper, the coefficients *k*_2_ and *k*_4_ were extracted from the regions of low and high *φ*, respectively (equivalently, low and high Φ, respectively). As observed from the slopes of the dashed lines in Fig. 3c, k_2_ = −5/3 and *k*_4_ = 2, fit the data well. For copper dispersions, *k*_1_ ≈ 1.12 × 10^−3^ and *k*_3_ = 0.0125 sV^2^ fit data well. With graphite dispersions neither the presence of a surface oxide nor sintering was expected. Therefore, in the case of graphite dispersions, *τ*_*h*−*ox*_ = 0, *τ*_*h*−*s*_ = 0 and *τ*_*h*_ = *τ*_*h*−*f*_. Since the value of *k*_2_ is material independent, *k*_2_ = −5/3 is a good fit for graphite dispersions as well. For graphite dispersions, *k*_1_ ≈ 1.8 × 10^−4^ fits data well. The models for heal time are plotted as solid lines in Fig. 3d. Models based on Eq. 1 based on the extracted coefficients are plotted with solid lines along side experimental data. There is good corroboration between model and experiment.

#### Steady state heal resistance, *R*_*h*∞_

The second performance parameter, i.e. the current carrying capacity of the self healed interconnect is defined by the steady state heal resistance of *R*_*h*∞_. The resistance of this bridge would depend on the conductivity of the particle, area of cross-section of the bridge and the length of the bridge. Since there remains in effect only one bridge irrespective of the concentration, the steady state heal impedance is independent of *φ*. The only condition on *φ* is that there be enough particles (i.e. at least *s*/2*r*_*p*_) in the volume space (2*π*/3)(*s*/2)^3^ available to form a bridge. Therefore *φ* > 2(2*r*_*p*_/*s*)^2^ is a strict requirement.

2 $${R}_{h{\rm{\infty }}}=\{\begin{array}{cc}{k}_{5}s/({\sigma }_{p}{r}_{p}^{2}) & {\rm{i}}{\rm{f}}\,\phi \ge 2{(2{r}_{p}/s)}^{2}\\ {\rm{\infty }} & {\rm{i}}{\rm{f}}\,\phi  < 2{(2{r}_{p}/s)}^{2}\end{array}$$

Here *k*_5_ is a constant coefficient that is determined by the effective contact cross-section area between the sintered particles of the bridge. If the bridge was a solid wire of radius *r*_*p*_ and length *s*, *k*_5_ would take on its minimum value of 1. However, the effective cross-section area is lesser than *π*${r}_{p}^{2}$ and therefore, *k*_5_ > 1.

**Figure 4:**
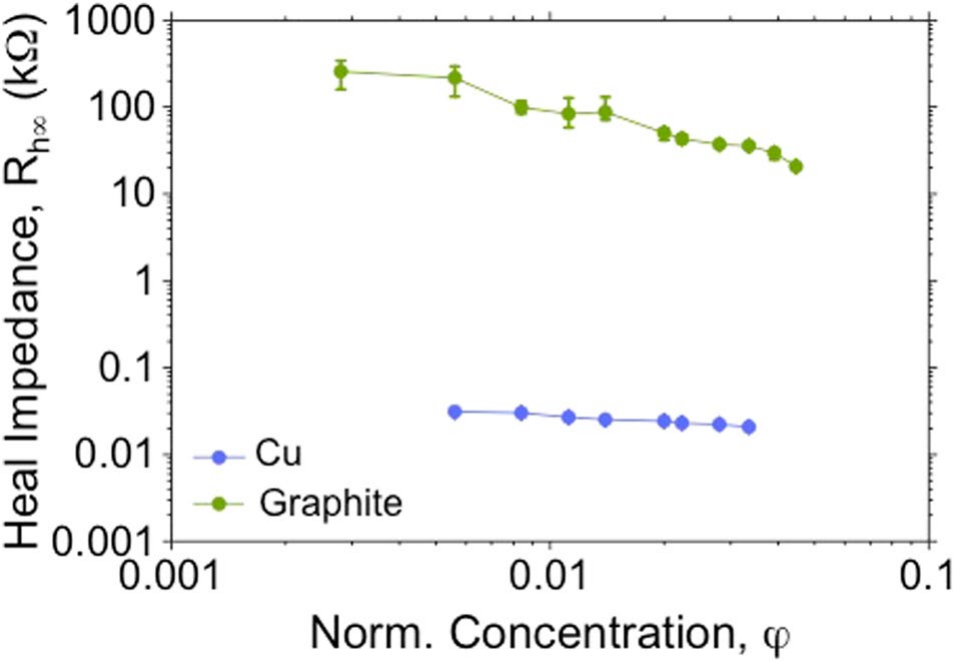
Dependence of steady state heal resistance, *R*_*h*∞_ on *φ* for copper and graphite dispersions in silicone oil.

Figure 4 shows the dependence of *R*_*h*∞_ as a function of *φ* for copper and graphite dispersions. For copper, as predicted, *R*_*h*∞_ is almost independent of *φ* as sintering results in the formation of just one bridge irrespective of the concentration. In the case of graphite, there is no sintering and the presence of multiple parallel bridges results in a lower heal resistance and a stronger dependence on *φ*. However, the advantage of using copper is that *R*_*h*∞_ is low. From the experimental data for copper dispersions, *k*_5_*s*/(*σ*_*p*_${r}_{p}^{2}$) ≈ 30 Ω. Using *σ*_*p*_ = 5.9 × 10^7^ S/m, *k*_5_ = 225.

#### Cross-impedance, *Z*_*c*_

The third performance parameter is the cross-talk between adjacent interconnects defined by the cross-impedance, *Z*_*c*_. Cross-talk is undesirable since any high frequency signal passing through one interconnect leaks onto the adjacent interconnects thereby corrupting the signal it carries. This places a bound on the maximum operating frequency.

With the addition of self-healing ability, cross-talk is expected to become significant as the packaging of the dispersion using conduits induces a significantly three dimensional profile to the interconnect. This increase in cross-talk is a direct consequence of the packaging and would be present irrespective of whether self-healing is utilized or not. For *φ* greater than the percolation limit, the dispersion would behave like a metallic ink and the capacitive coupling would be similar to that between two three dimensional interconnects. For the range of *φ* investigated in this work, i.e. much lower than the percolation threshold, the cross-talk is not expected to vary significantly. However, as *φ* increases, a small amount of non-homogeneity results in more particle clusters and the clusters being electrically connected to the interconnect. This effectively reduces cross-impedance and increases cross-talk. In the presence of packaging, it can be expected that the cross-impedance, *Z*_*c*_, is purely capacitive with a weak dependence on *φ* such that,

3 $${Z}_{c}={k}_{6}(d/\omega ){\phi }^{{k}_{7}}$$

Here *ω* is the frequency of the small signal, *d* is the distance between the interconnect, *k*_6_ a constant coefficient (with units Ω/*ms*) that depends on the effective overlap cross-section of the two interconnects and the permittivity of the medium between the interconnects. The parameter *k*_7_ another dimensionless constant coefficient.

**Figure 5:**
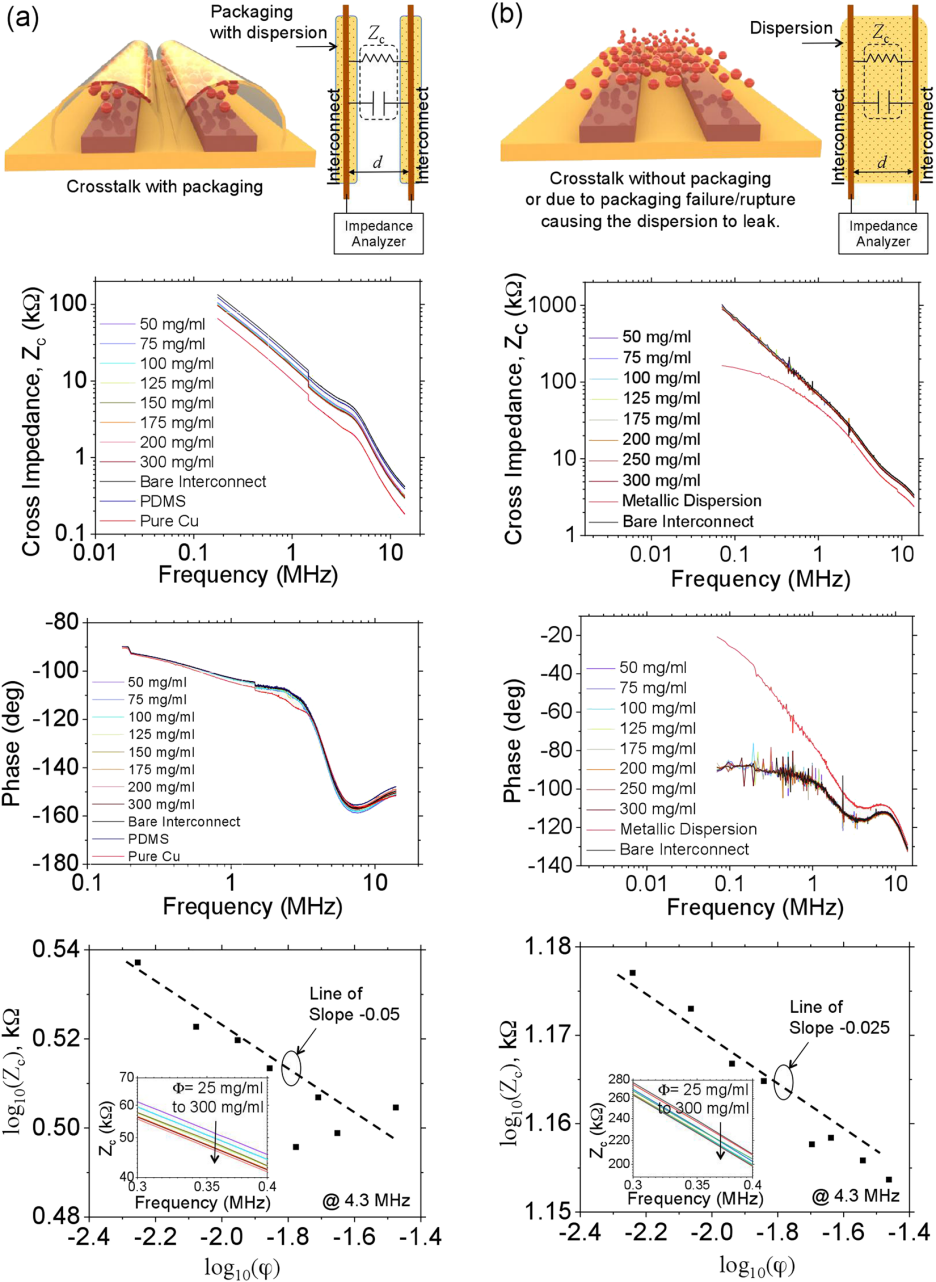
Impedance spectroscopy and dependence of cross-impedance, *Z*_*c*_ on *φ* (**a**) with packaging (**b**) without packaging. The dispersion used was copper in silicone oil.

Figure 5a illustrates the experimental setup to determine *Z*_*c*_ via impedance spectroscopy with the packaging present (see Methods). In the presence of packaging, *Z*_*c*_ is only slightly dependent on *φ* and decreases with increasing *φ*. The impedance is capacitive with the phase at −90 deg. At higher frequencies, the phase deviates to −180 deg indicating the presence of another pole. From the plot of log(*Z*_*c*_) versus log(*φ*) we find that *k*_7_ ≈ −0.05 and *k*_6_ ≈ 5 × 10^13^ Ω/ms.

An extreme but important case is related to the failure of the packaging or when no packaging is used (Fig. 5b). In such a case, the dispersion would overlap two adjacent interconnects. For *φ* greater than the percolation limit, this would lead to an undesirable short circuit. For any general *φ*, *Z*_*c*_ would be defined by a parallel combination of the resistive and capacitive component. For very high dispersion concentrations and low frequencies the resistive component would be much smaller than the capacitive component and determine *Z*_*c*_. However, for moderate dispersion concentrations or relatively high frequencies the capacitive component would still be the one that determines *Z*_*c*_. Figure 5b illustrates the experimental setup to determine *Z*_*c*_ in the absence of packaging or when the packaging fails. For very large *φ* (metallic dispersion), the *Z*_*c*_ is low and resistive as seen from the phase approaching 0 deg. For lower concentrations the impedance is largely capacitive with multiple poles and zeroes showing up at higher frequencies. Therefore, for all practical purposes, Eq. 3 still determines the cross-impedance with *k*_7_ ≈ −0.025 and *k*_6_ ≈ 1.5 × 10^14^ Ω/ms.

A clear advantage of using no packaging is that self-healing can be achieved without the complexity involved with the packaging for as long as the dispersion concentration is kept low. From the point of view of cross-impedance, the use of packaging creates a leaky path at all frequencies thereby lowering the effective impedance. For example, PDMS has approximately the same relative permittivity of silicone oil and using it as a packaging material results in a larger effective cross-section overlap between two adjacent interconnects as its sheet-thickness would be significant compared to the thickness of the interconnect while at the same time requiring a larger headroom to package the dispersion over the interconnect. On the other hand, the dispersion used without packaging smears itself into a sufficiently thin film that offers higher impedance. It is therefore seen in Fig. 5a,b that *Z*_*c*_ with packaging is lower than that without.

### Optimization

The dependence of the three performance parameters, *τ*_*h*_, *R*_*h*∞_ and *Z*_*c*_, on *φ* is summarized by Eqs. 1, 2 and 3. Of these *R*_*h*∞_ is independent of *φ* (for particles that sinter) and only imposes of a strict condition of *φ* ≥ 2(2*r*_*p*_/*s*)^2^. For values of *φ* below this limit, there is no healing. With regards to *Z*_*c*_, there is a small but steady dependence on *φ* with *Z*_*c*_ steadily decreasing with increasing *φ*. A critical limit is to keep *φ* well below the percolation threshold to avoid short circuits between adjacent interconnects in the case of failure of packaging. Another point of view is that since *Z*_*c*_ does not depend on *φ* very strongly for low *φ*, the packaging of the dispersion may be completely avoided. This is a significant advantage eFAH offers as compared to other techniques for self-healing. The final parameter, the heal time *τ*_*h*_, shows an interesting dependence on *φ*. For metallic particles that can sinter (preferred for their low *R*_*h*∞_), *τ*_*h*_ first decreases with increasing *φ* and then begins to increase as *φ* increases. This parameter is the most critical from the point of view of applications as well as from the point of view of the dependence on the dispersion concentration.

**Figure 6:**
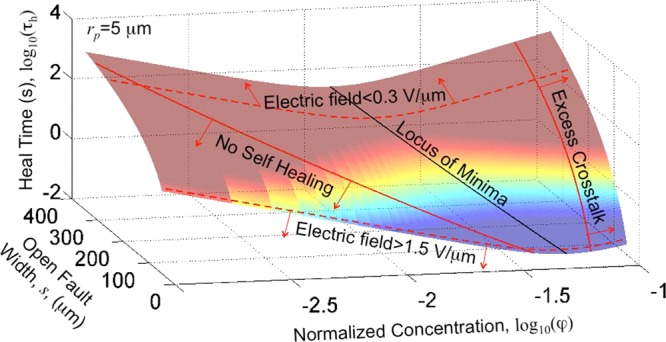
Optimization. Dependence of heal time *τ*_*h*_ on *φ* and length of open fault *s*.

Figure 6 shows the dependence of *τ*_*h*_ on *φ* for different gap lengths, *s*. While all other parameters regarding the dispersion and particles can be engineered, the gap length of the open fault is a parameter that cannot be predicted. The gap length determines the electric field strength, the length and resistance of the heal and defines the volume space and number of particles available (because they are polarized by the field) for healing. Figure 6 also shows the critical bounds on *φ* due to limits imposed by cross-talk and the steady state heal impedance. Although not the focus of this work, there also exist bounds on the electric field (shown in dashed lines). For very low fields, the dipole-dipole forces cannot overcome friction and there is no healing. For very large fields, healing occurs very rapidly, but the heal can be destroyed by the current surge for low *R*_*l*_^43^. These four boundary lines mark the optimum region of operation for effective healing. From Eq. 1, it is seen that the minimum heal time occurs when *φ* = *φ*_*opt*_, defining the optimum dispersion concentration

4 $${\phi }_{opt}={(\frac{-{k}_{1}{k}_{2}}{{k}_{3}{k}_{4}}\frac{{\eta }_{f}}{{\varepsilon }_{f}}{(\frac{s}{{r}_{p}})}^{1-3{k}_{4}}\frac{{s}^{2}}{{V}_{dc}^{2-{k}_{4}}})}^{1/({k}_{4}-{k}_{2})}$$

Table 2 summarizes the values of the model coefficients. Setting *k*_1_ = 1.12 × 10^−3^, *k*_2_ = −5/3, *k*_3_ = 0.0125 and *k*_4_ = 2, *φ*_*opt*_ = (0.075(*η*_*f*_/*ε*_*f*_)(*r*_*p*_/s)^5^*s*^2^)^3/11^. For *s* = 200 *μ*m, this corresponds to a *φ* = 0.017 which for copper is equivalent to Φ = *μ*_*p*_*φ* ≈ 150 mg/ml.

**Table 2:** Model coefficients.

Coefficient	Unit	Theoretical Estimate	Emperical Estimate
*k*_1_		(0, ∞)	1.12 × 10^−3^
*k*_2_		−5/3	−5/3
*k*_3_	sV^*k*^_4_	(0, ∞)	0.0125
*k*_4_		2	2
*k*_5_		[1, ∞)	225
*k*_6_	Ω/ms	(0, ∞)	5 × 10^13^
*k*_7_		(−∞, 0)	−0.025 to −0.05

This value of *φ* = 0.017 is the optimum value of the dispersion concentration for any dispersion made with conductive particles that have a surface oxide and eventually sinter during healing via eFASH.

## Conclusion

This work focused on the impact of dispersion concentration on the performance parameters of self-healing using the eFASH technique. Three performance parameters studied were the heal time, *τ*_*h*_, steady state heal impedance, *R*_*h*∞_, and cross-talk determined by the cross-impedance, *Z*_*c*_. The dispersion concentration was best described by the normalized concentration, *φ* that allowed for an easy comparison of different dispersions irrespective of the conductive particle used. In other words, once the optimum *φ* = *φ*_*opt*_ is achieved, the ideal dispersion concentration based on any particle in weight/volume is *φ*_*opt*_*μ*_*p*_. Therefore, this work predicts a general result in this regard.

In general, two kinds of conductive particles could be used to prepare the dispersion - particles that sinter after healing via eFASH and particles that do not sinter after healing. The former is preferable since the heal then becomes permanent and has a very low value of *R*_*h*∞_ which in some sense is the whole point of performing healing.

Considering only particles that sinter, the heal time *τ*_*h*_ was seen to first decrease and then increase with increasing *φ*. From the experiments of Fig. 2, the decrease of *τ*_*h*_ is due to the reducing inter-particle distance with increasing *φ* resulting in shorter timescales for particles to chain up to form the bridge. The increase in *τ*_*h*_ for higher *φ* was speculated to be due to the increase in breakdown time of any surface oxide or insulating film present at the particle-particle interface in the bridge. The breakdown time increased with increasing *φ* since higher concentrations permitted multiple parallel bridges across the gap resulting in a lower voltage drop across the heal (and hence a lower field in the surface oxide).

For particles that could sinter, the steady state heal impedance *R*_*h*∞_ was seen to be almost independent of *φ* as healing resulted in only one bridge irrespective of the particle concentration. The only condition on *φ* was that there should be enough particles to form at least one bridge across the open fault.

The impact of implementing eFASH on cross-talk between adjacent interconnects was studied for two cases, the first when the dispersion was packaged and contained over the interconnect and the second when no packaging was used. In both cases, increasing the dispersion concentration reduced cross-impedance, *Z*_*c*_ and increased cross-talk. The dependence of *Z*_*c*_ on *φ* was small for the range of concentrations used in this work. However, when *φ* began to approach the percolation limit, *Z*_*c*_ significantly decreased in the latter case with the resistive component dominating at lower frequencies as expected. A major advantage of eFASH was highlighted in the fact that for moderate values of *φ*, the dispersion could be left unpackaged without any significant impact on cross-talk. This possibility offers a significant advantage from the point of manufacturing and implementation of eFASH at large scales.

The optimum *φ* = *φ*_*opt*_ was identified after considering the two bounds placed by *R*_*h*∞_ i.e. the minimum *φ* needed to achieve healing and by *Z*_*c*_ i.e the maximum *φ* permissible before cross-talk became significant. Within these bounds, *φ*_*opt*_ was identified as that which achieved minimum heal time. For particles that sintered, *φ*_*opt*_ ≈ 0.017. If copper particles are considered, this corresponded to an ideal dispersion concentration of 150 mg/ml.

## Methods

### Dispersion preparation

Dispersions of copper in silicone oil were prepared using copper microspheres of average diameter 10 *μ*m (Alfa Aesar Product No. 042689) in silicone oil having a kinematic viscosity of 300 cSt (S. D. Fine-Chem Limited, Product No. 25072). Dispersions of various concentrations were were prepared by measuring the weight of copper microspheres to be dispersed in a known volume of silicone oil (resulting in different Φ). The dispersion was sonicated for 1 hour while keeping the oil at 80 C followed by mechanical stirring. No other stabilization techniques was used. While low concentration Φ < 300 mg/ml (and *φ* < 0.033) dispersions were relatively homogeneous, dispersions of higher concentration resulted in some clustering. Dispersions with Φ > 400 mg/ml (and *φ* > 0.044) became difficult to prepare with consistency.

Dispersions of graphite flakes of average diameter 7 *μ*m–10 *μ*m (Alfa Aesar Product No. 43480) in silicone oil were prepared using the same technique as dispersions of copper in silicone oil. The range of *φ* (i.e. 0.002 ≤ *φ* ≤ 0.044) was maintained to enable a comparison regarding heal time and heal impedance from copper and graphite dispersions.

### Measurement of heal time

Heal time as a function of *φ* was measured using the experimental setup shown in Fig. 2. To measure the heal time, the plot of current versus time was used. The plot was obtained by using a Keithley 2410 to apply the voltage (thereby emulating the occurrence of the fault), monitor the time and measure the current. The start of the experiment was time *t* = 0. The time when the current spiked represented the time of sintering and end of healing *t* = *τ*_*h*_.

### Measurement of heal impedance

Heal impedance was measured by probing the heal at the end of healing process with a digital multimeter.

### Measurement of cross-impedance

All cross-impedance measurements were performed using the Keysight E4990A Impedance Analyzer. Two copper tracks of 1 mm wide and 2 mm apart (*d* = 2 × 10^−3^ m) were used for the study. The cross-impedance between the bare interconnects was first measured to provide the reference *Z*_*c*_.

To measure cross impedance with the packaging, the packaging was prepared using a technique similar to that discussed in^28^ (Also see Section S6, Figs. S82 to S84, Supplementary Material). The interconnect layout was used to create a 3D printed mold with the interconnects represented by raised features. Polydimethyl siloxane (PDMS) was molded to form vesicles that mapped to the positions of the interconnects. The patterned PDMS was laid over the interconnects and the dispersion was injected into the vesicles resulting in the dispersion being packaged and isolated over the interconnects. The impedance spectrum between the adjacent interconnects was first measured without any dispersion thereby illustrating the impact of the PDMS packaging alone. Dispersions of varying concentrations (50 ≤ Φ ≤ 300 mg/ml, equivalently 0.0055 ≤ *φ* ≤ 0.033) was used to study the impact of *φ* on *Z*_*c*_.

To measure cross impedance without packaging, dispersions of varying concentrations (50 ≤ Φ ≤ 300 mg/ml, equivalently 0.0055 ≤ *φ* ≤ 0.033) were dispersed between the bare interconnects with the dispersion straddling both interconnects along its length. The cross-impedance was measured to estimate the impact of *φ* on *Z*_*c*_.

## Supplementary information

Supplementary Information

Supplementary Information 1

Supplementary Information 2

Supplementary Information 3

Supplementary Information 4
