# Supplementary material for: Electric Field Assisted Self-Healing of Open Circuits with Conductive Particle-Insulating Fluid Dispersions: Optimizing Dispersion Concentration
Source: Sci Rep. 2019 Dec 23;9:19700. doi: 10.1038/s41598-019-55801-8 (PMC6928145; doi:10.1038/s41598-019-55801-8)
Supplement: Supplementary file 1 — Supplementary Information [file 41598_2019_55801_MOESM1_ESM.pdf]

Electric Field Assisted Self-Healing of Open Circuits with Conductive Particle-Insulating Fluid Dispersions: Optimizing Dispersion Concentration

Supplemental Material

Virendra Parab[1], Oppili Prasad[2,1] , Sreelal Pillai[3] and Sanjiv Sambandan[1,4]

1. *Department of Instrumentation and Applied Physics, Indian Institute of Science, Bangalore 560012*
2. *Centre for Nanoscience and Engineering, Indian Institute of Science, Bangalore 560012*
3. *Vikram Sarabai Space Centre, Indian Space Research Organisation, Trivandrum, India*
4. *Department of Engineering, University of Cambridge, Cambridge, UK CB30FF*

Corresponding Author email: ss698@cam.ac.uk

S1. SELF-HEALING WITH DIFFERENT TEMPERATURE

A. 30 degree Celsius

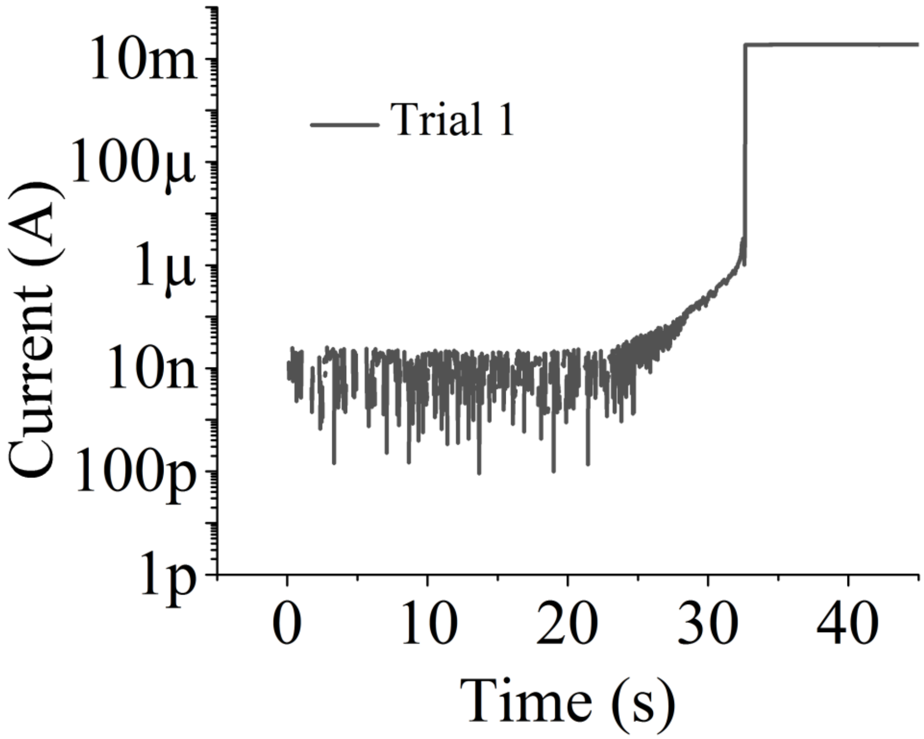

FIG. S1. Concentration = 25 mg/ml

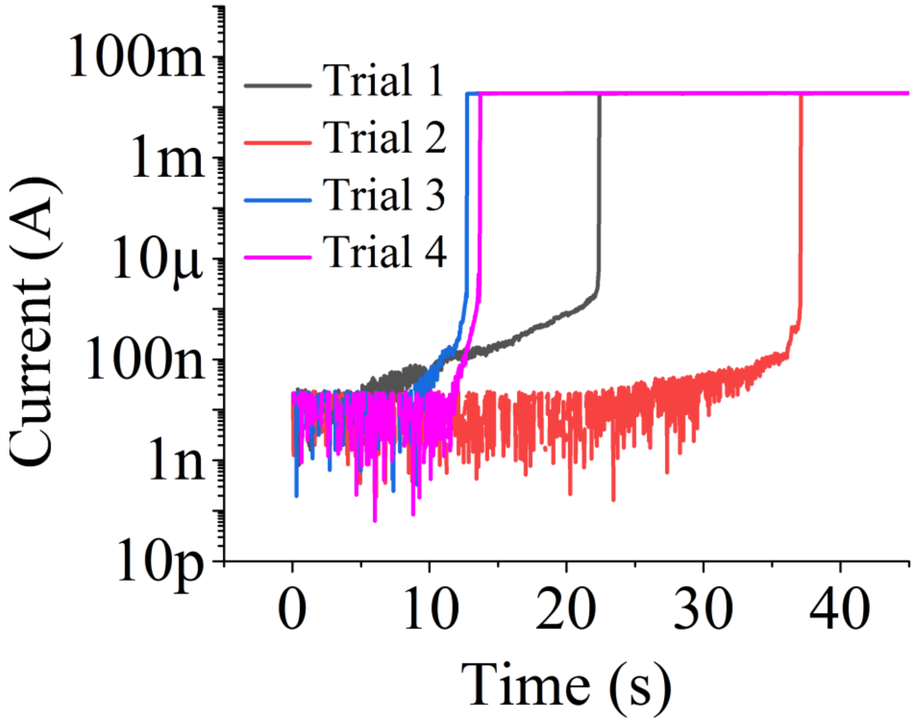

FIG. S2. Concentration = 50 mg/ml

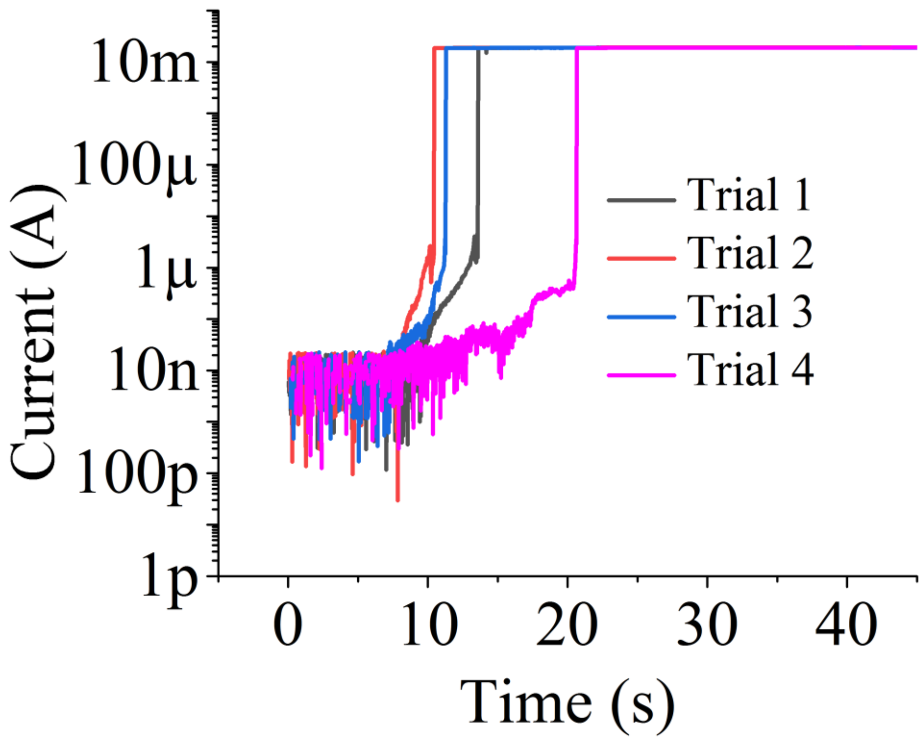

FIG. S3. Concentration = 75 mg/ml

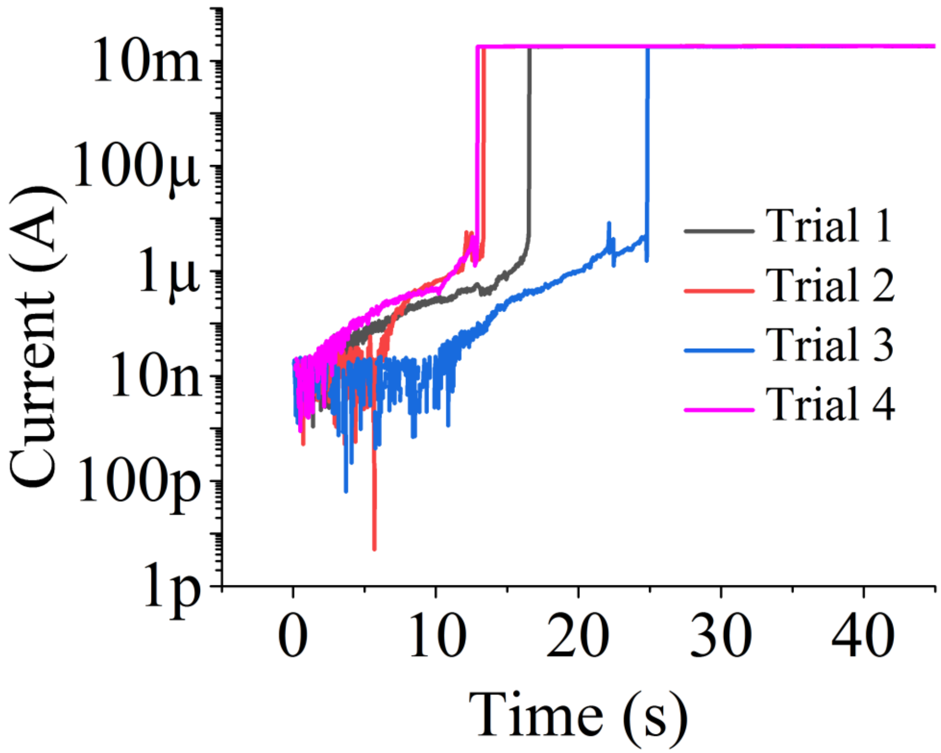

FIG. S4. Concentration = 100 mg/ml

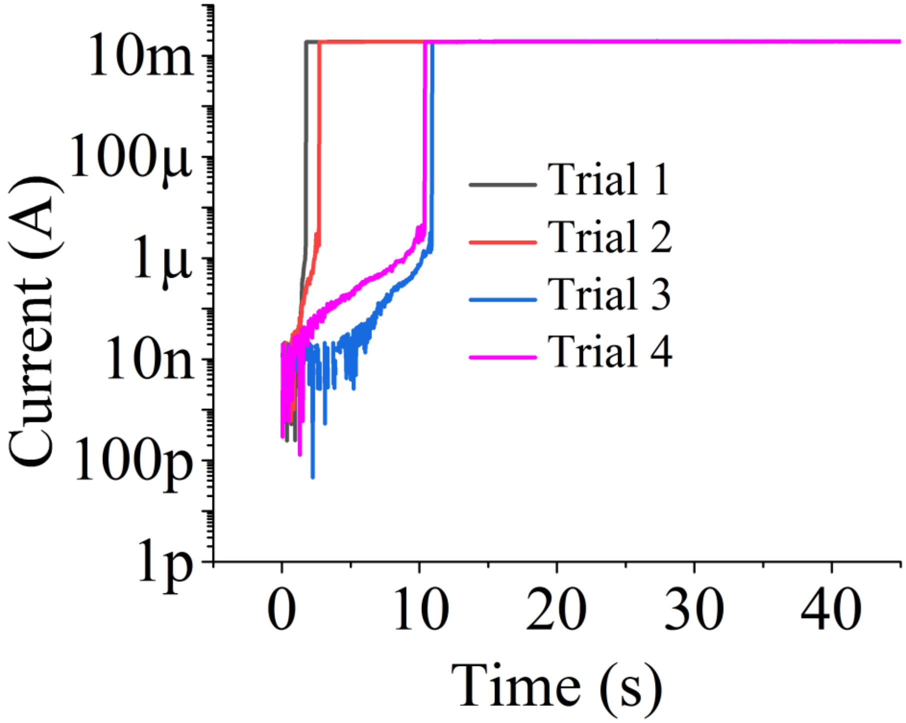

FIG. S5. Concentration = 125 mg/ml

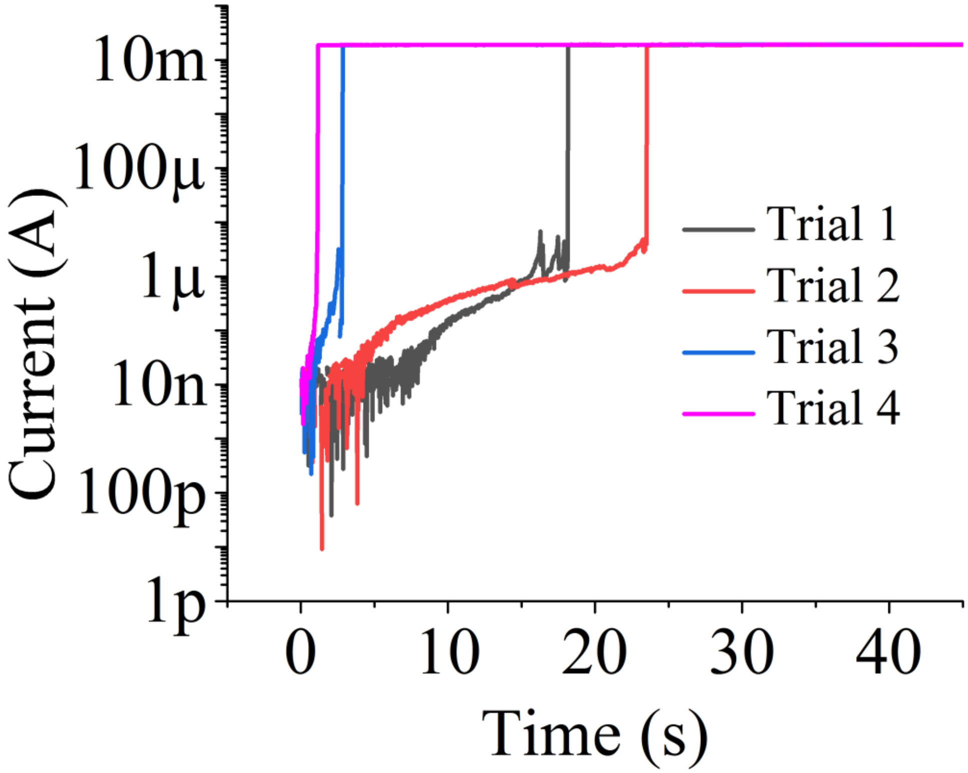

FIG. S6. Concentration = 175 mg/ml

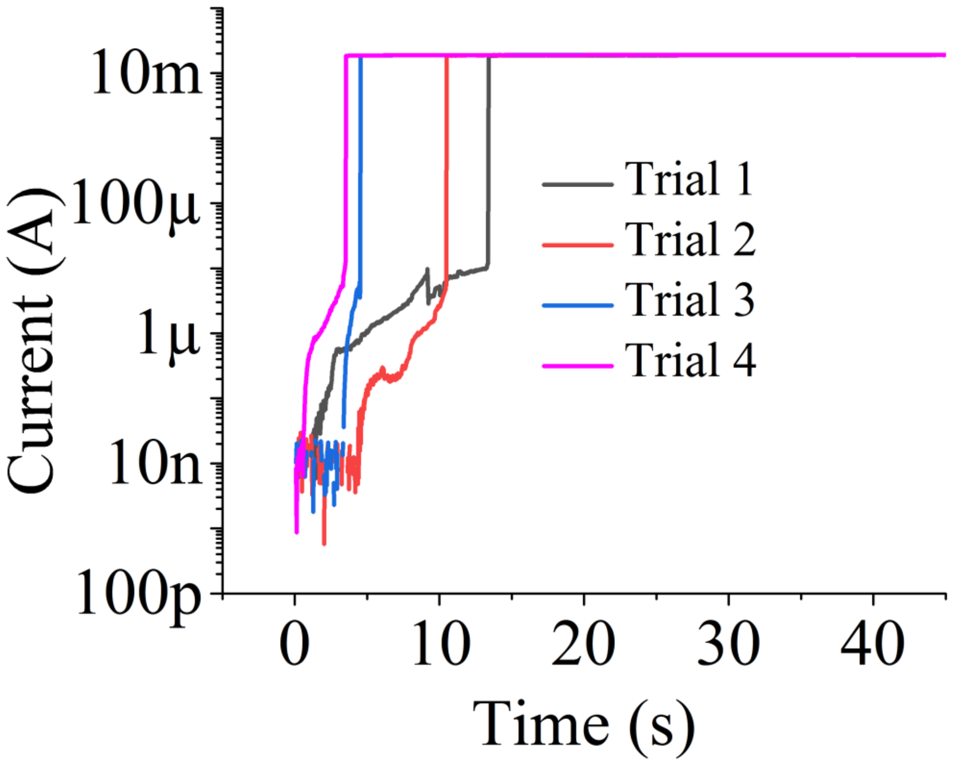

FIG. S7. Concentration = 200 mg/ml

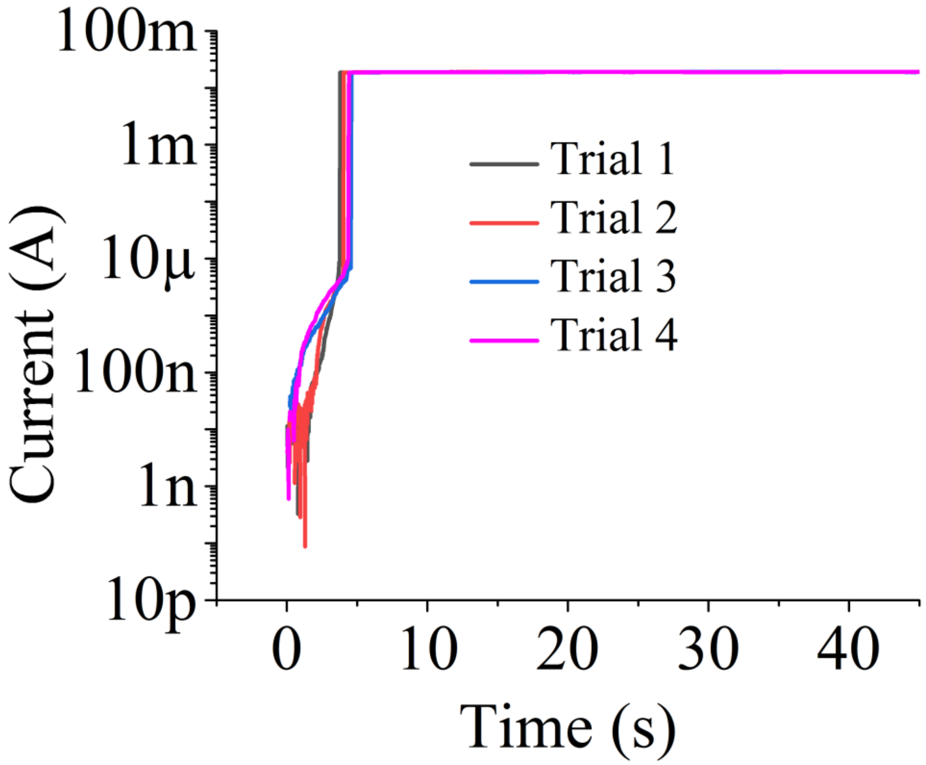

FIG. S8. Concentration = 250 mg/ml

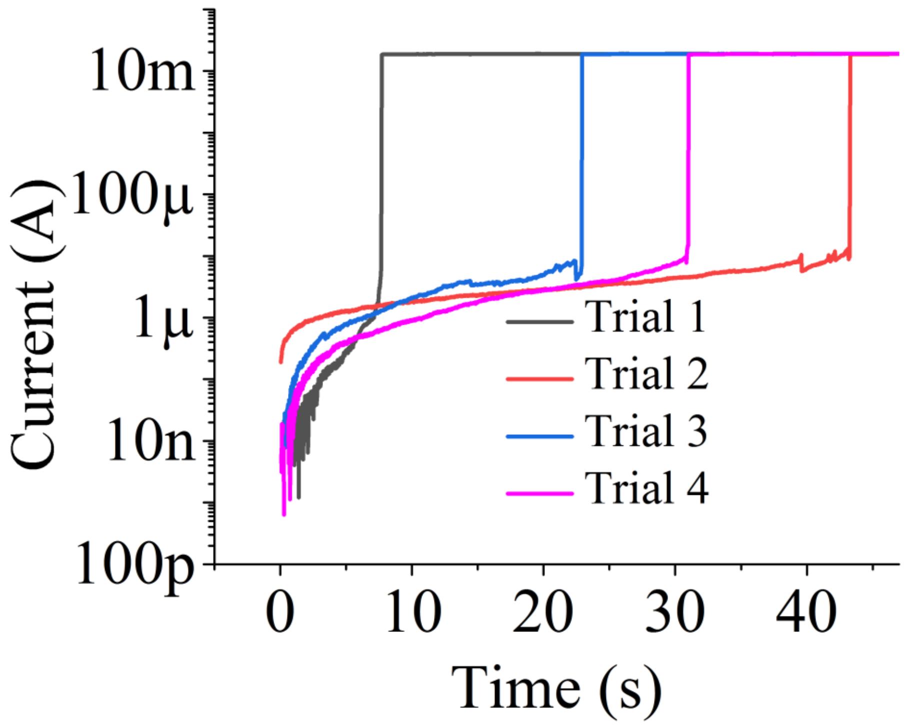

FIG. S9. Concentration = 300 mg/ml

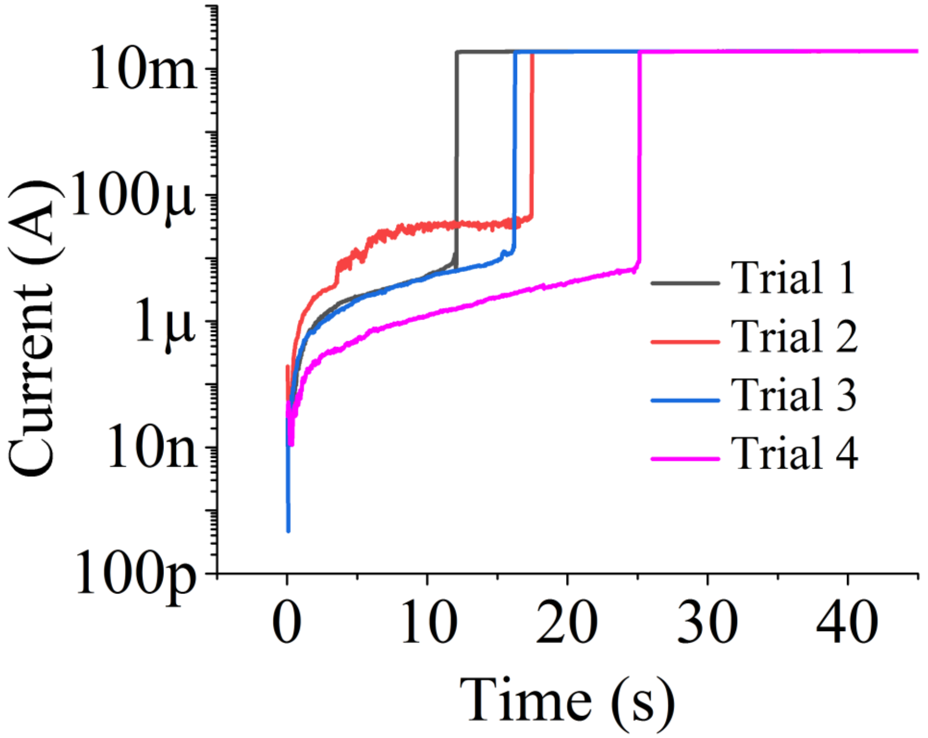

FIG. S10. Concentration = 350 mg/ml

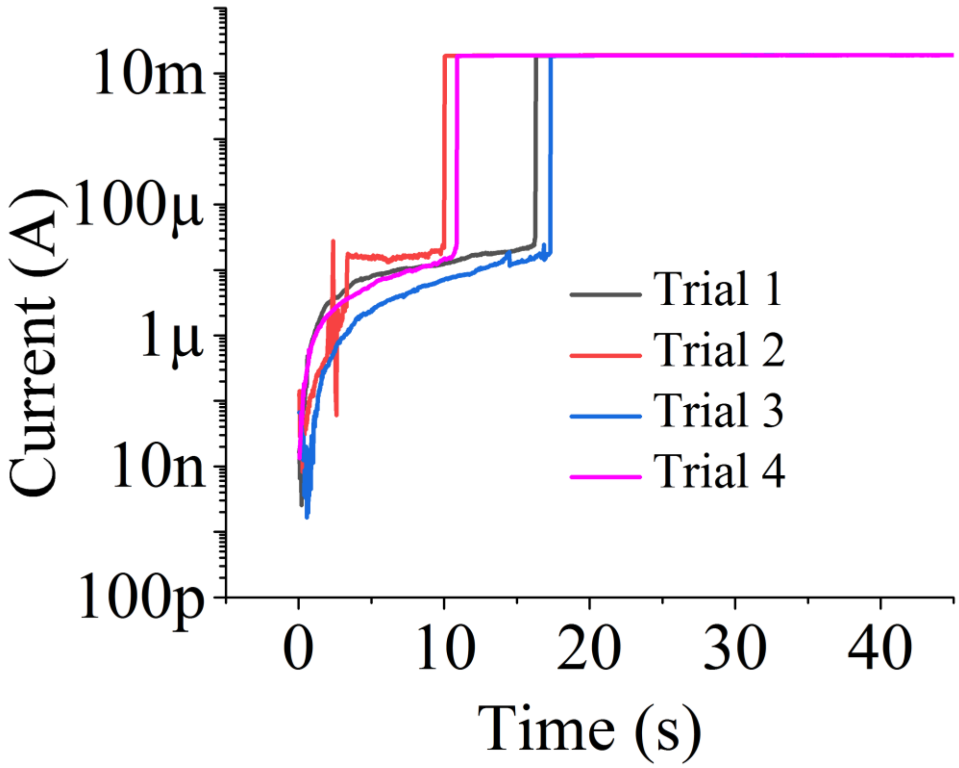

FIG. S11. Concentration = 400 mg/ml

B. 40 degree Celsius

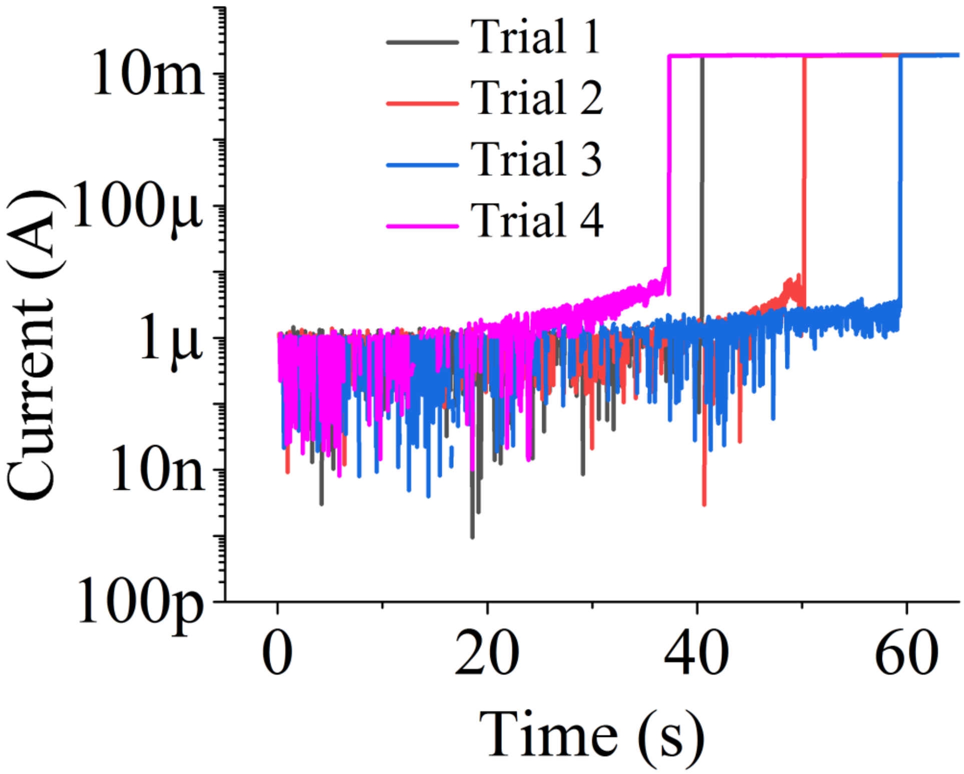

FIG. S12. Concentration = 25 mg/ml

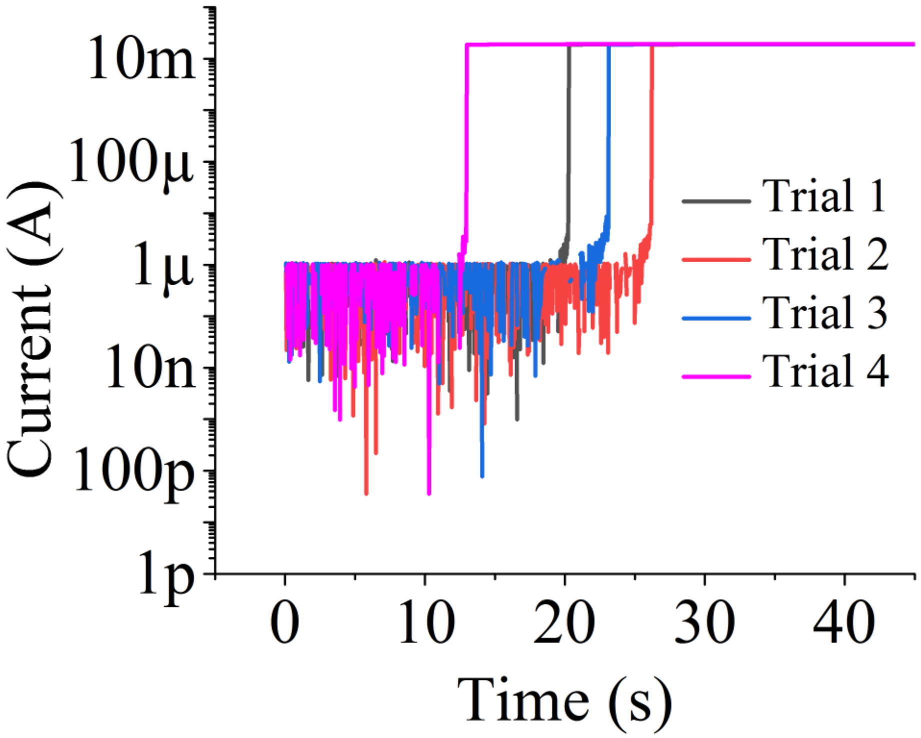

FIG. S13. Concentration = 50 mg/ml

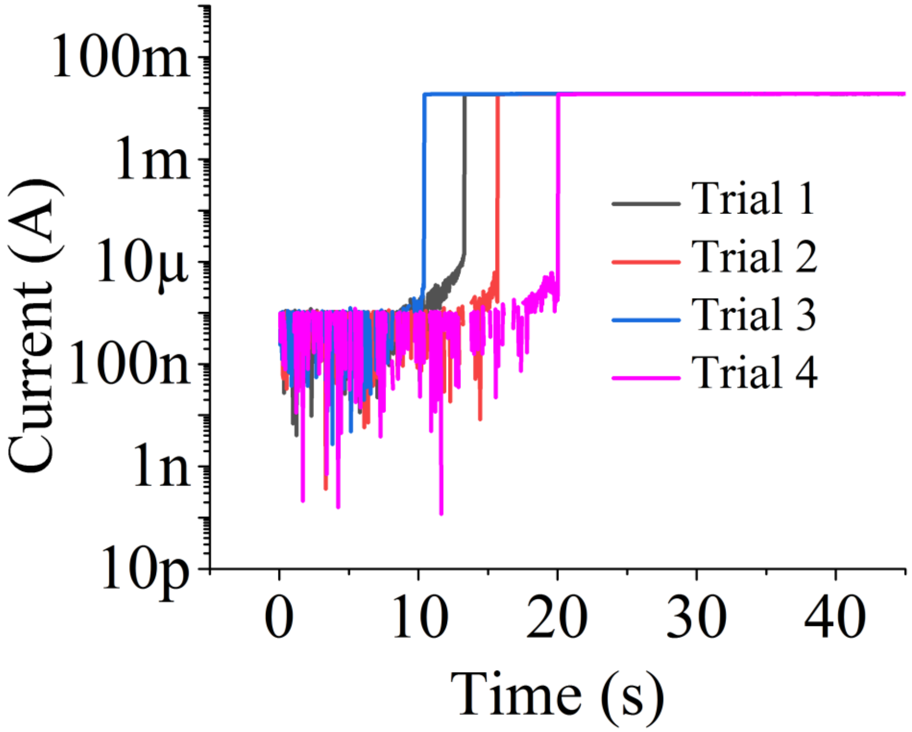

FIG. S14. Concentration = 75 mg/ml

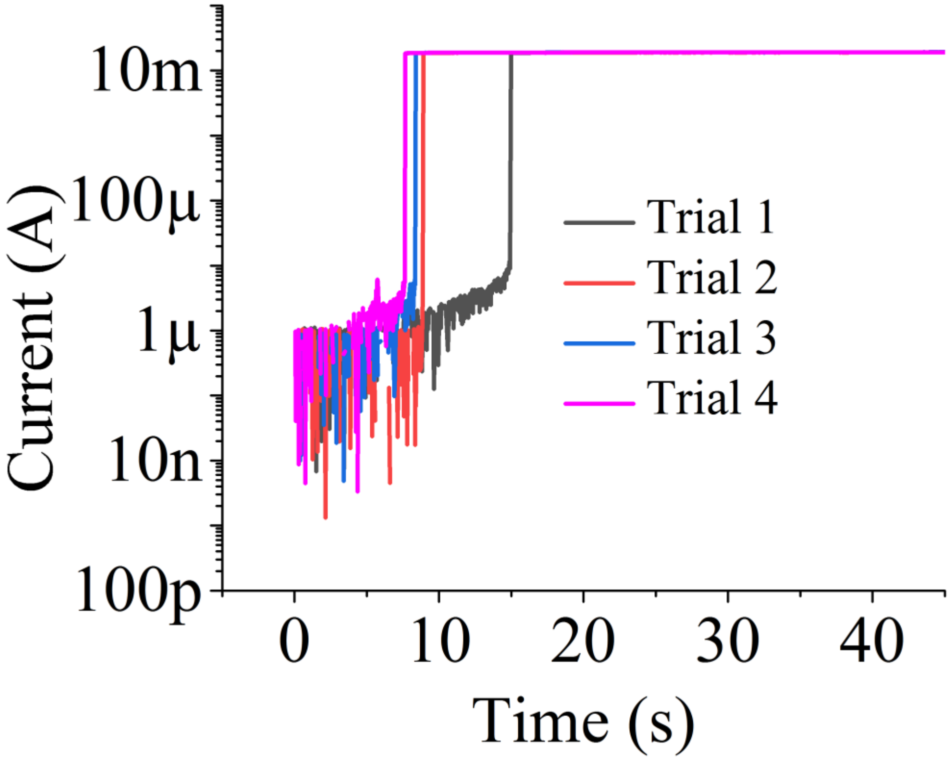

FIG. S15. Concentration = 100 mg/ml

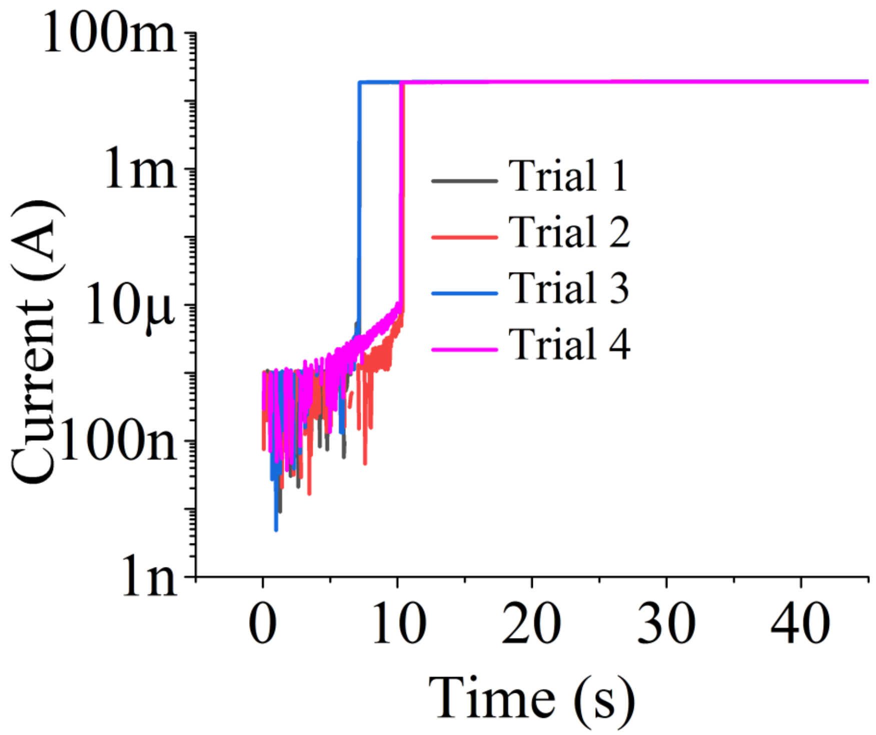

FIG. S16. Concentration = 125 mg/ml

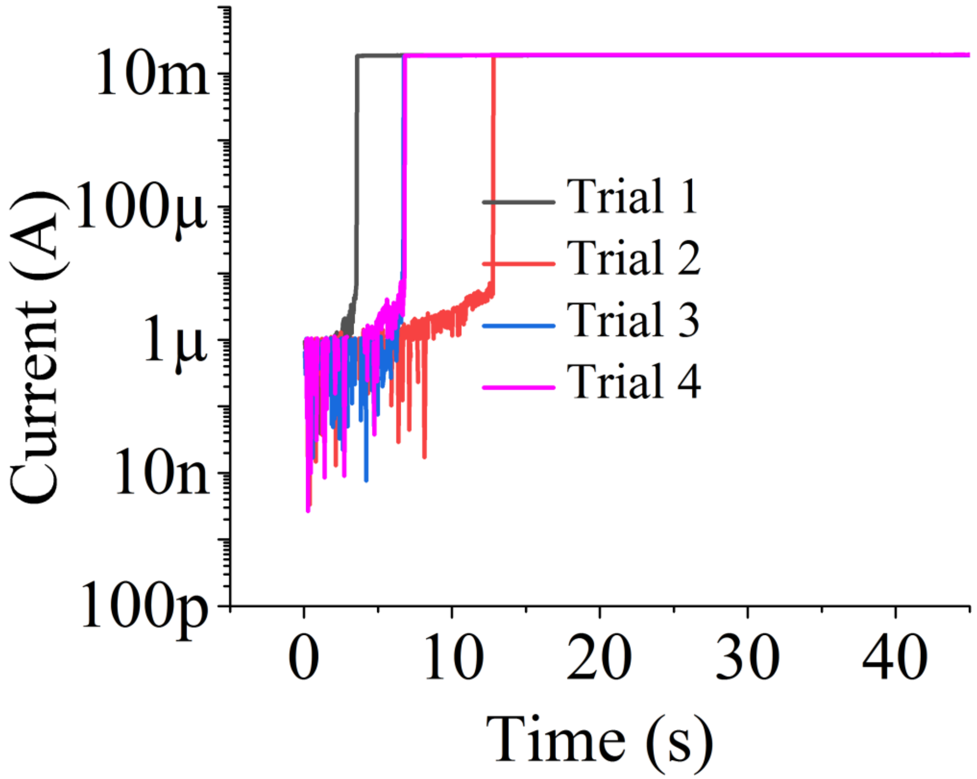

FIG. S17. Concentration = 175 mg/ml

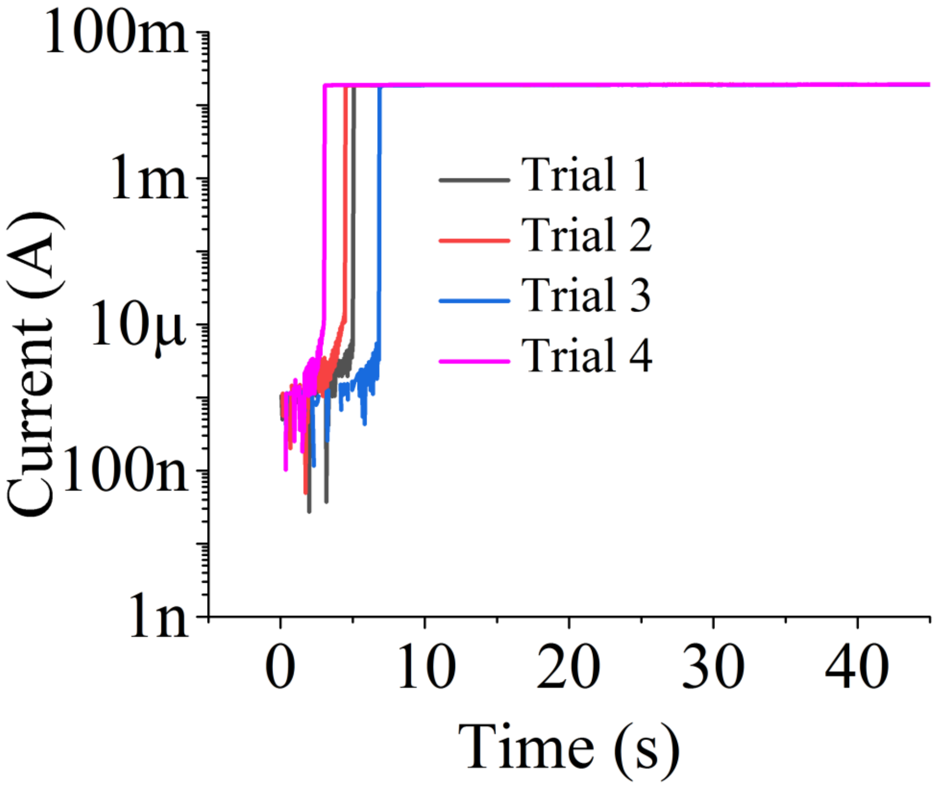

FIG. S18. Concentration = 200 mg/ml

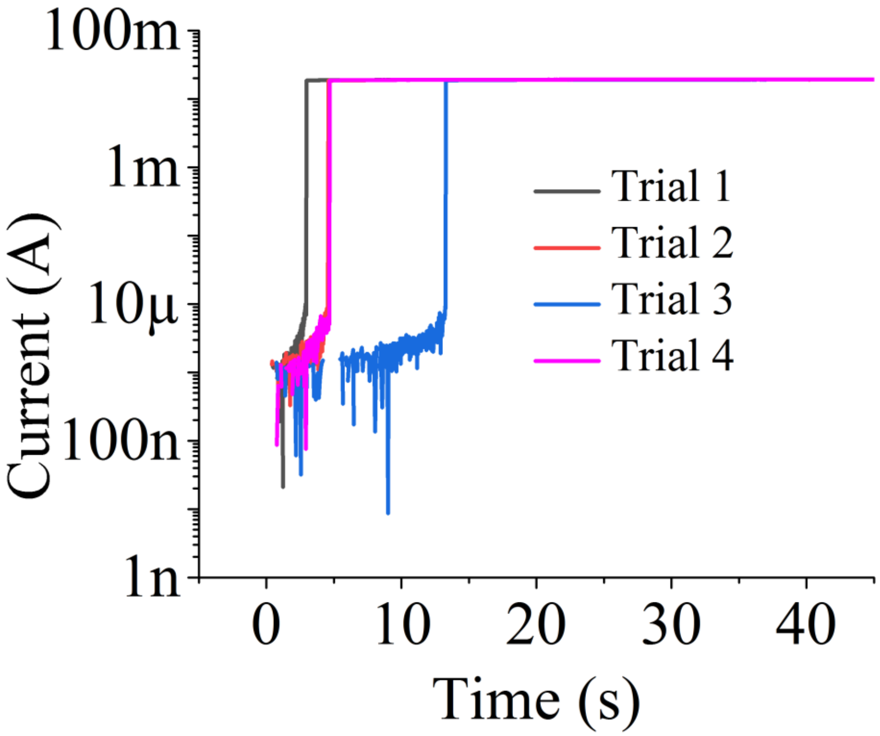

FIG. S19. Concentration = 250 mg/ml

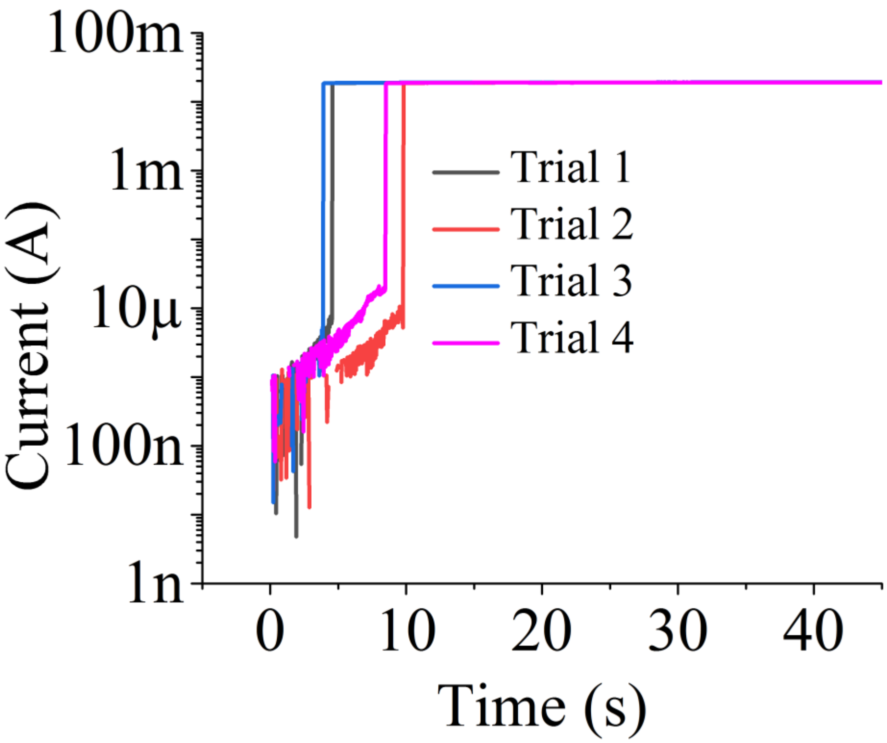

FIG. S20. Concentration = 300 mg/ml

C. 50 degree Celsius

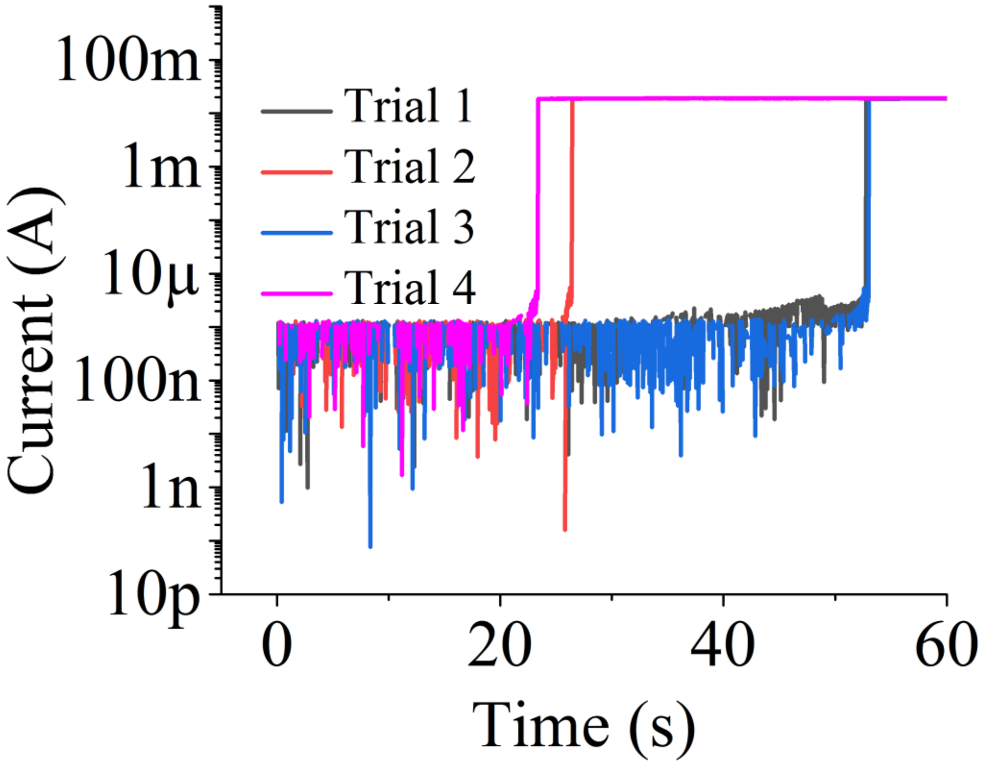

FIG. S21. Concentration = 25 mg/ml

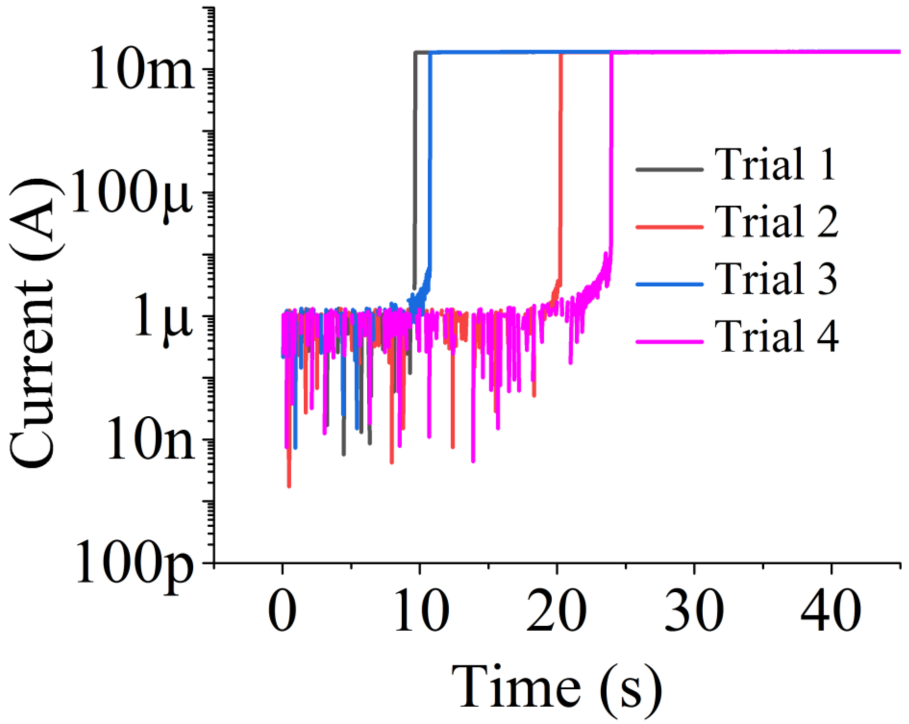

FIG. S22. Concentration = 50 mg/ml

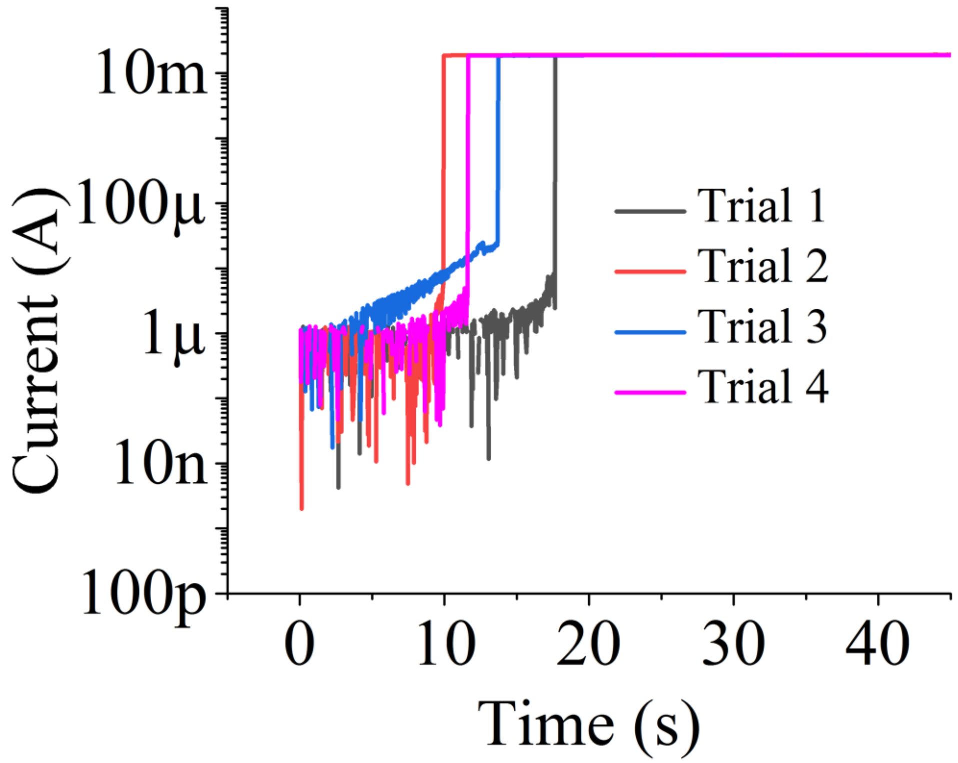

FIG. S23. Concentration = 75 mg/ml

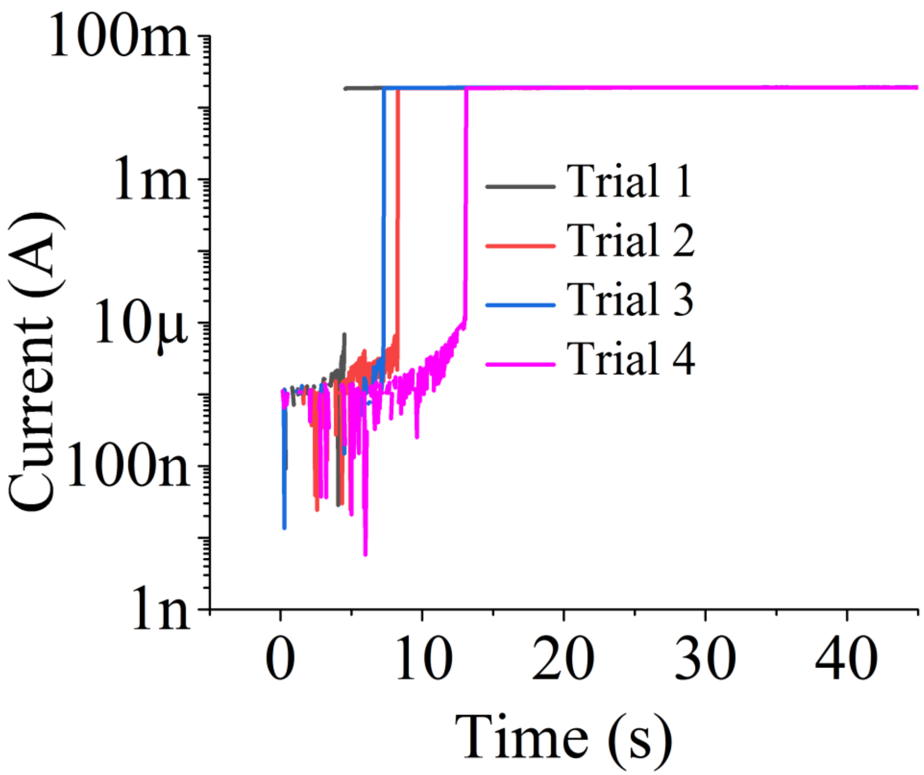

FIG. S24. Concentration = 100 mg/ml

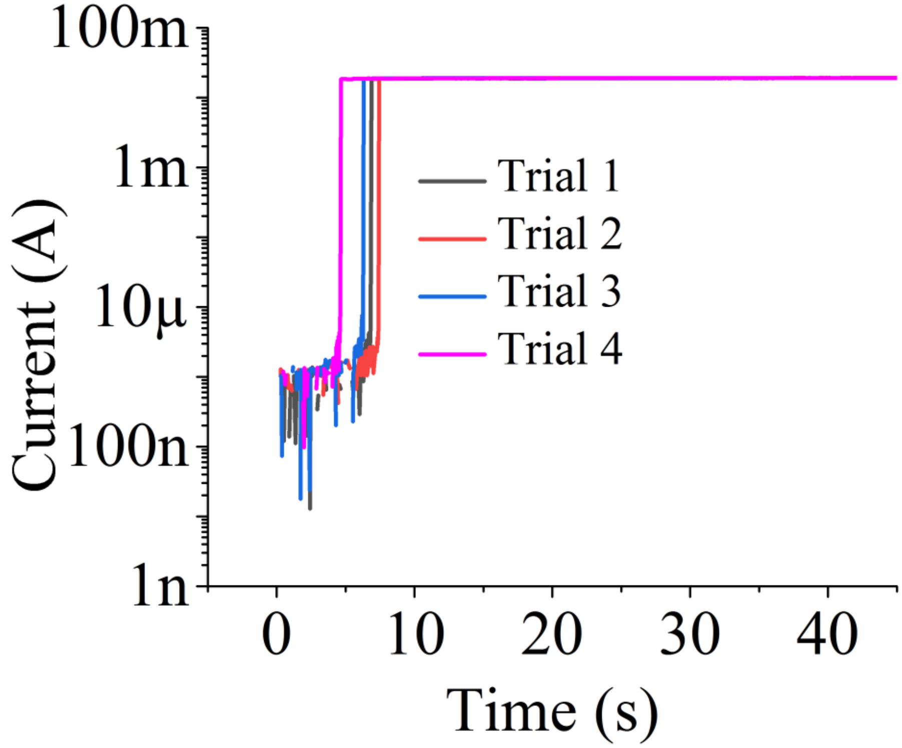

FIG. S25. Concentration = 125 mg/ml

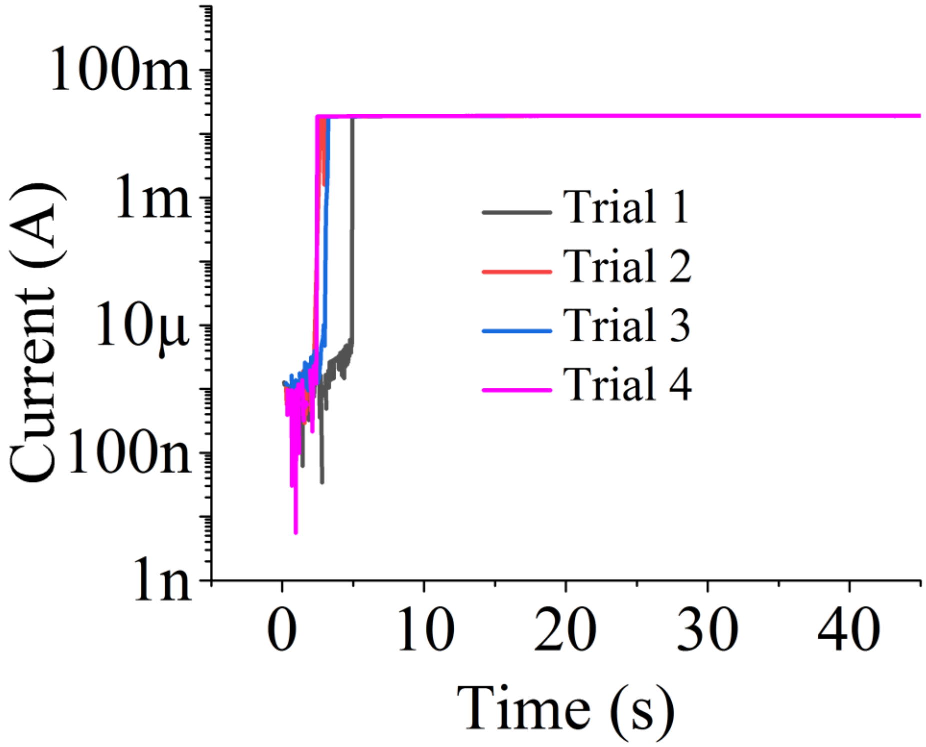

FIG. S26. Concentration = 175 mg/ml

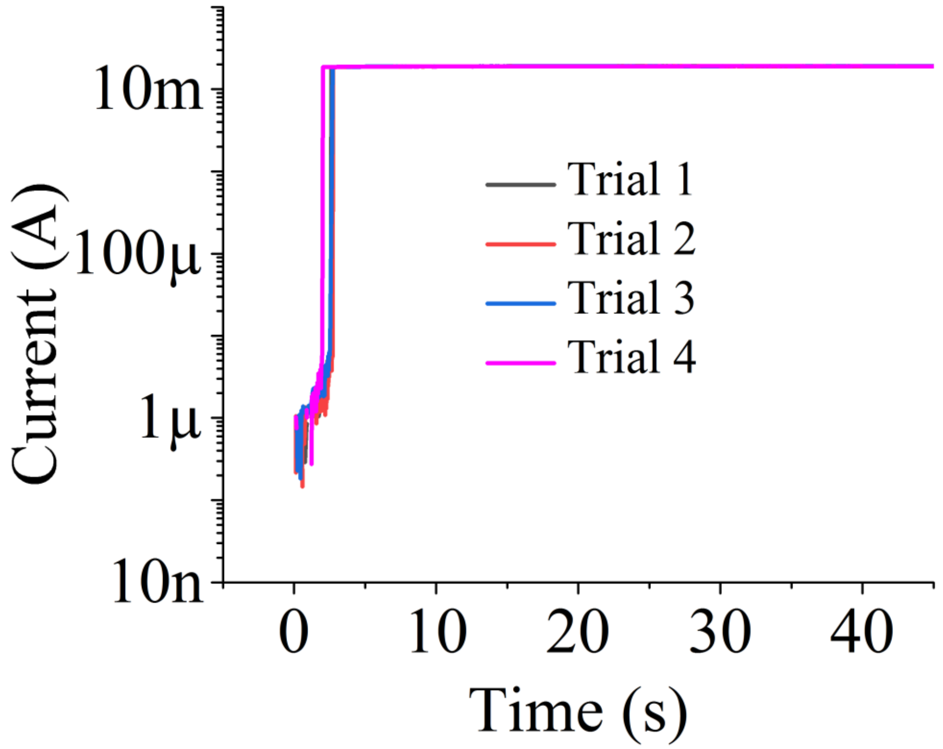

FIG. S27. Concentration = 200 mg/ml

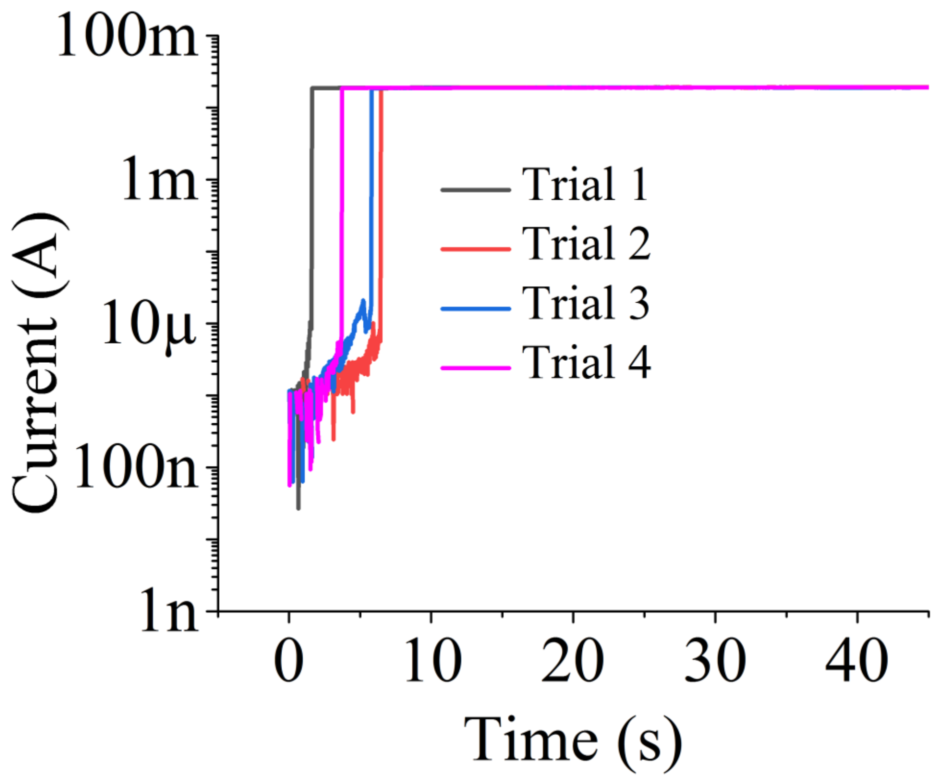

FIG. S28. Concentration = 250 mg/ml

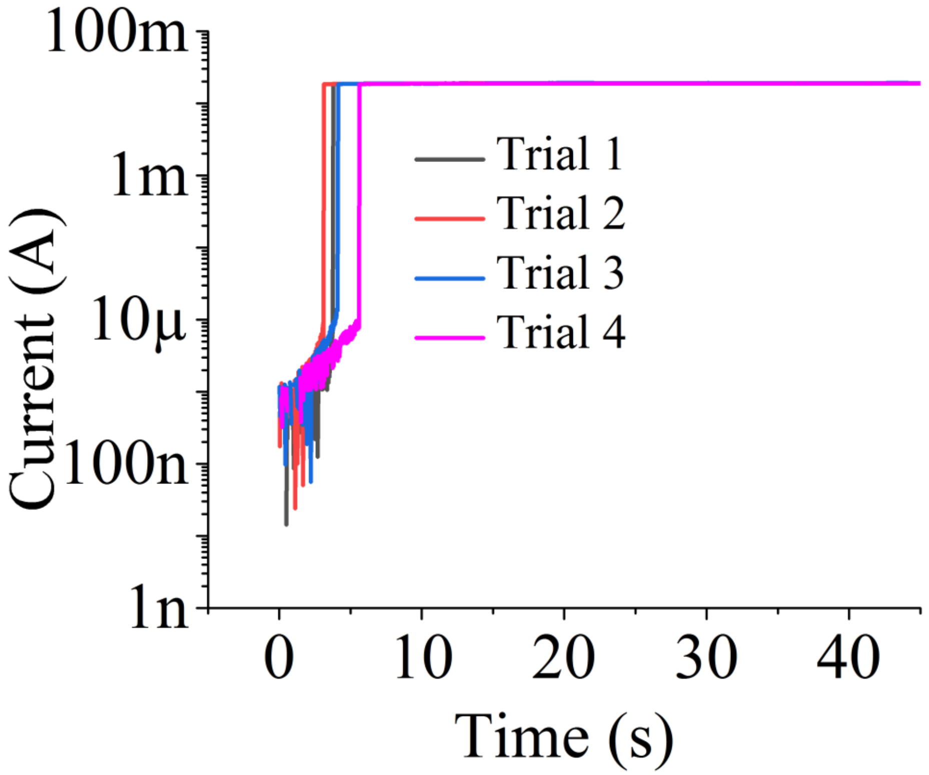

FIG. S29. Concentration = 300 mg/ml

D. 60 degree Celsius

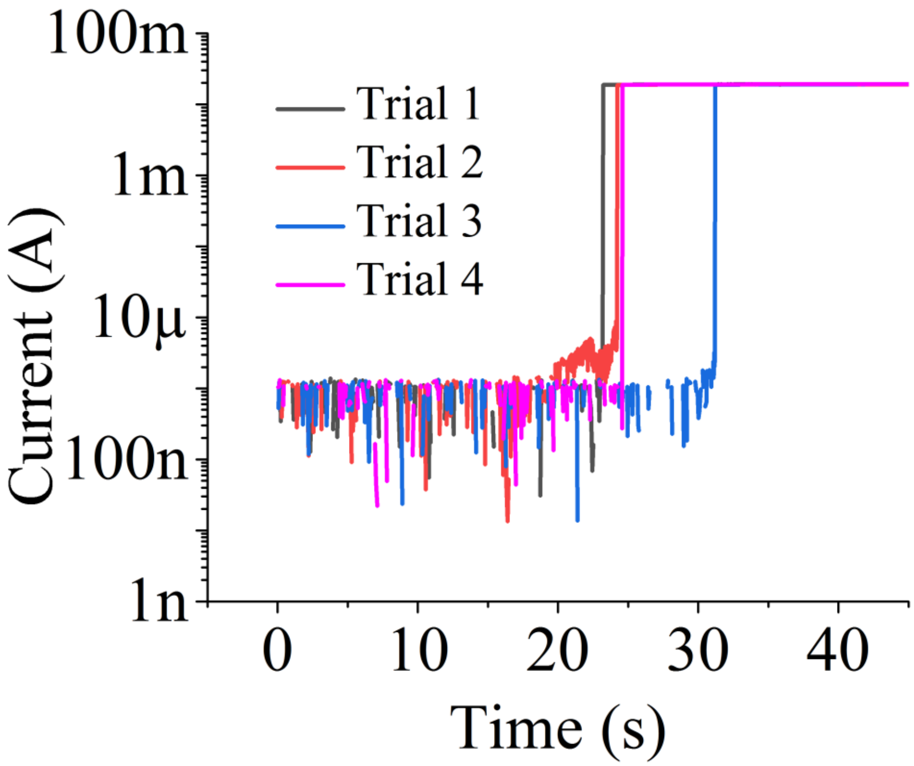

FIG. S30. Concentration = 25 mg/ml

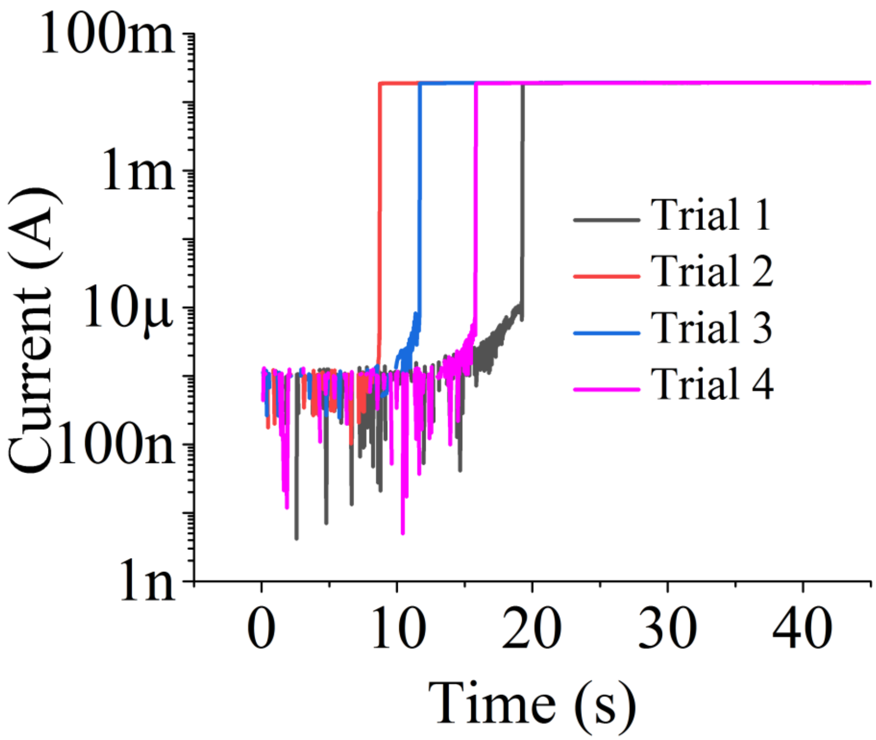

FIG. S31. Concentration = 50 mg/ml

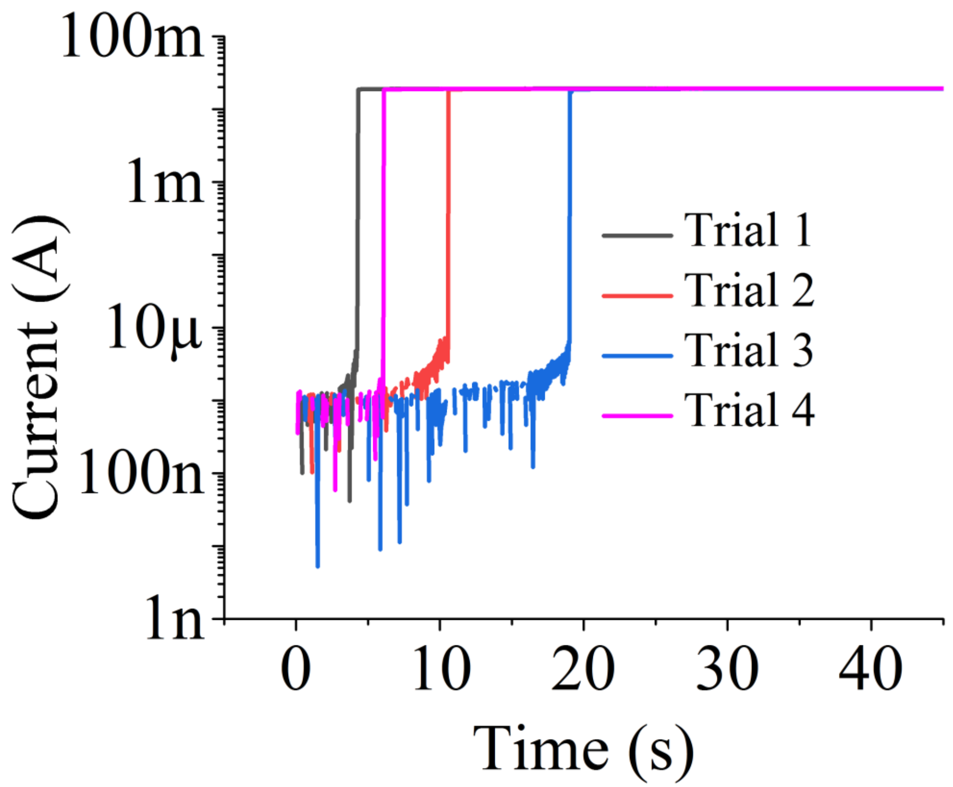

FIG. S32. Concentration = 75 mg/ml

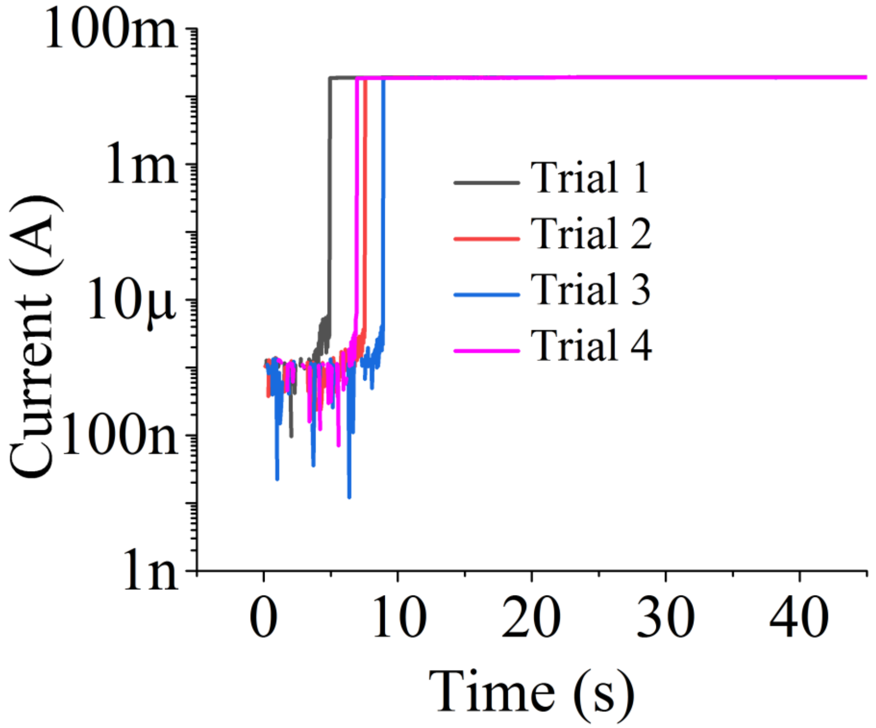

FIG. S33. Concentration = 100 mg/ml

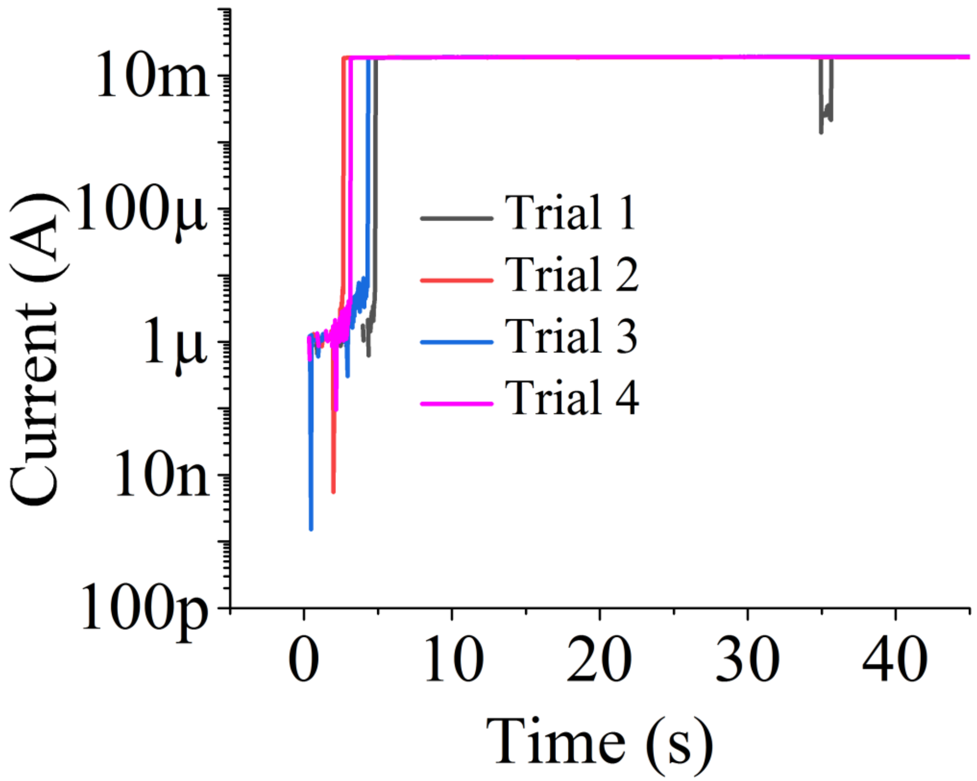

FIG. S34. Concentration = 125 mg/ml

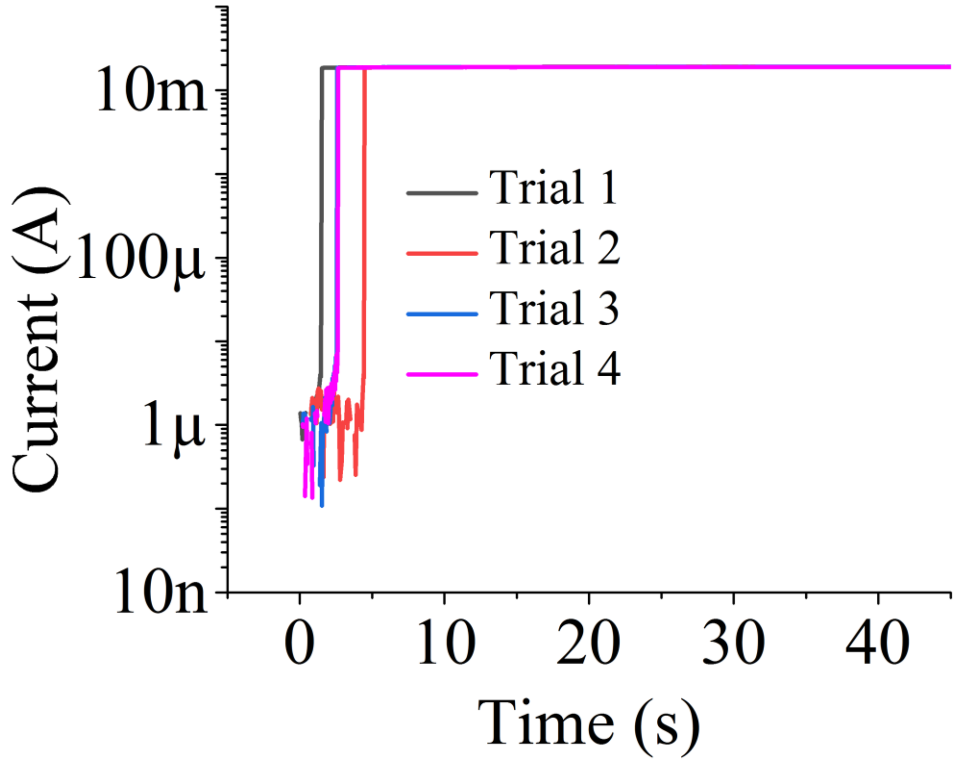

FIG. S35. Concentration = 175 mg/ml

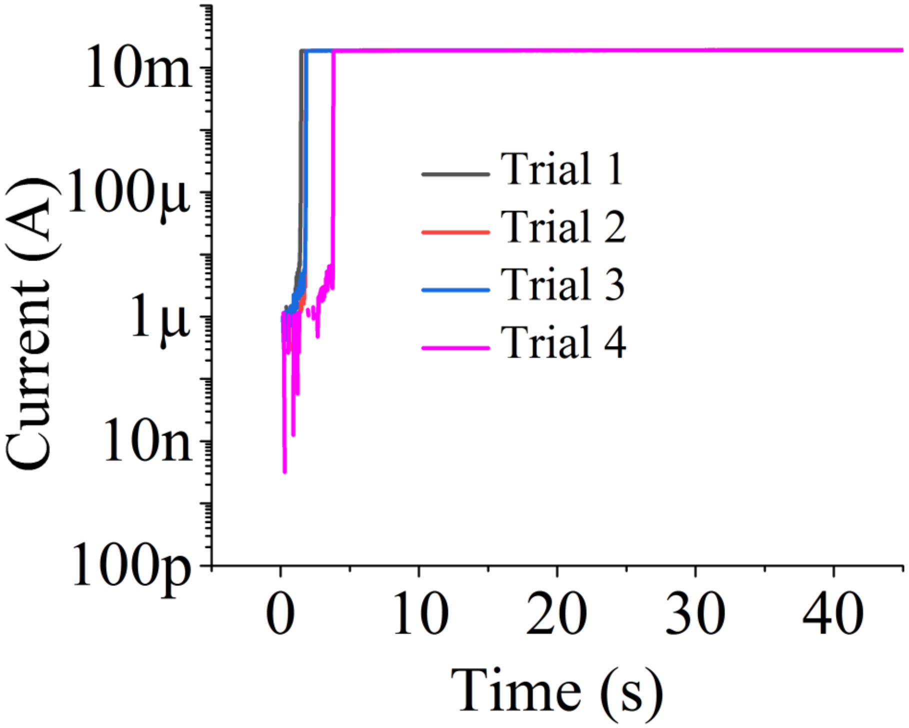

FIG. S36. Concentration = 200 mg/ml

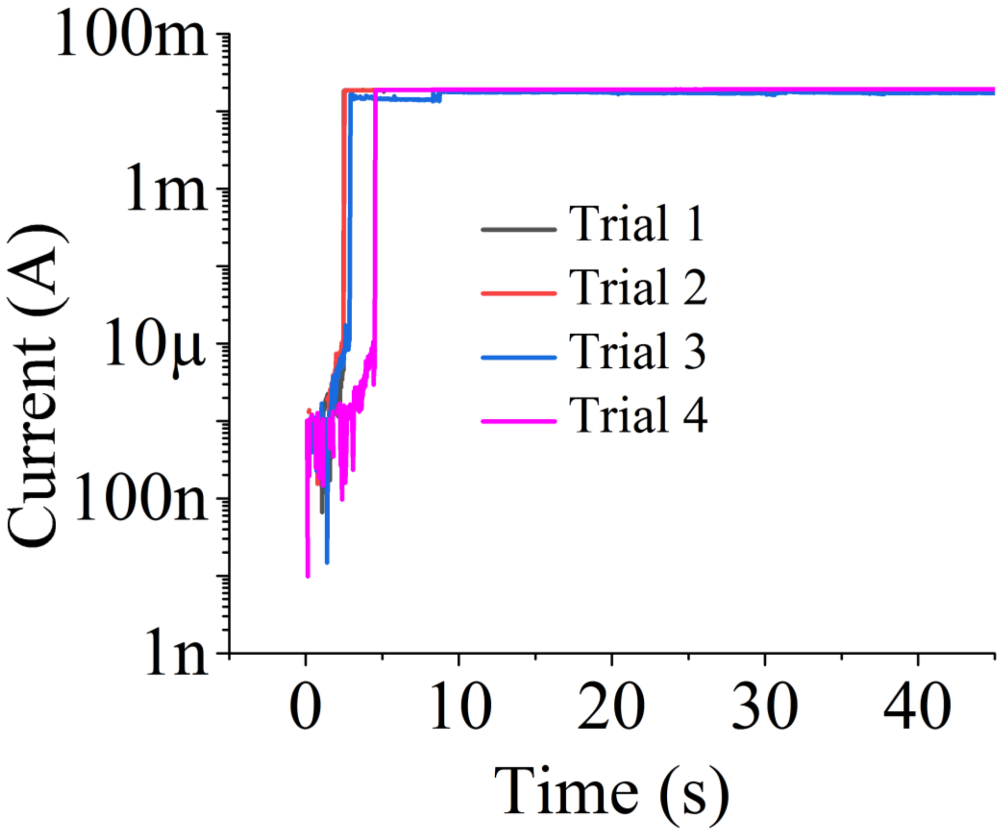

FIG. S37. Concentration = 250 mg/ml

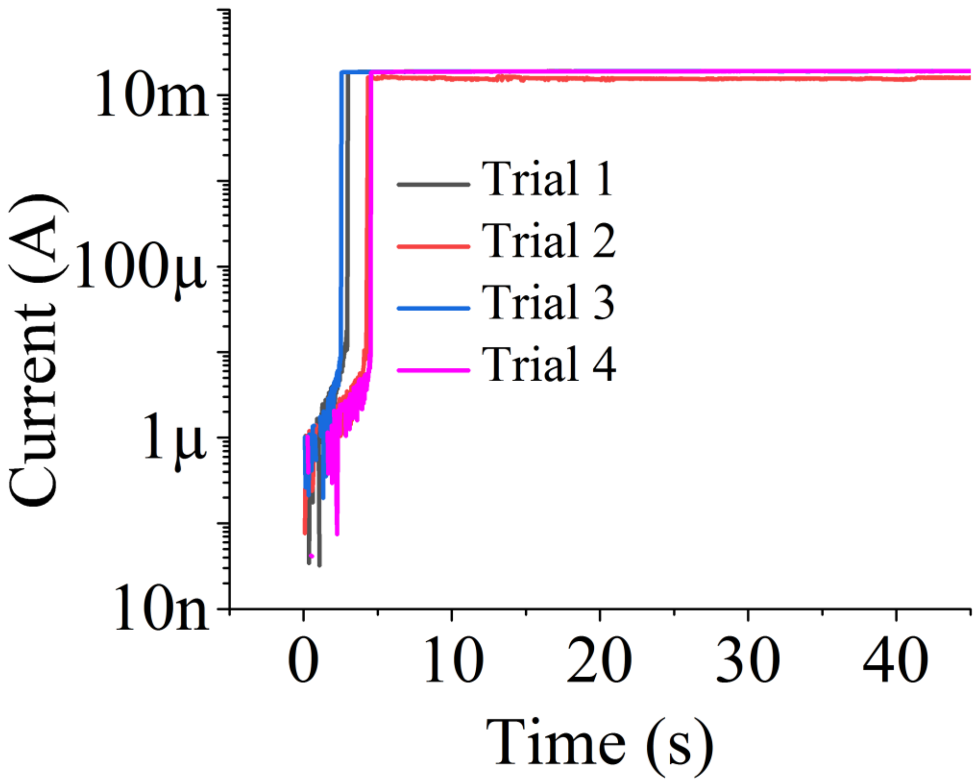

FIG. S38. Concentration = 300 mg/ml

E. 50 degree Celsius

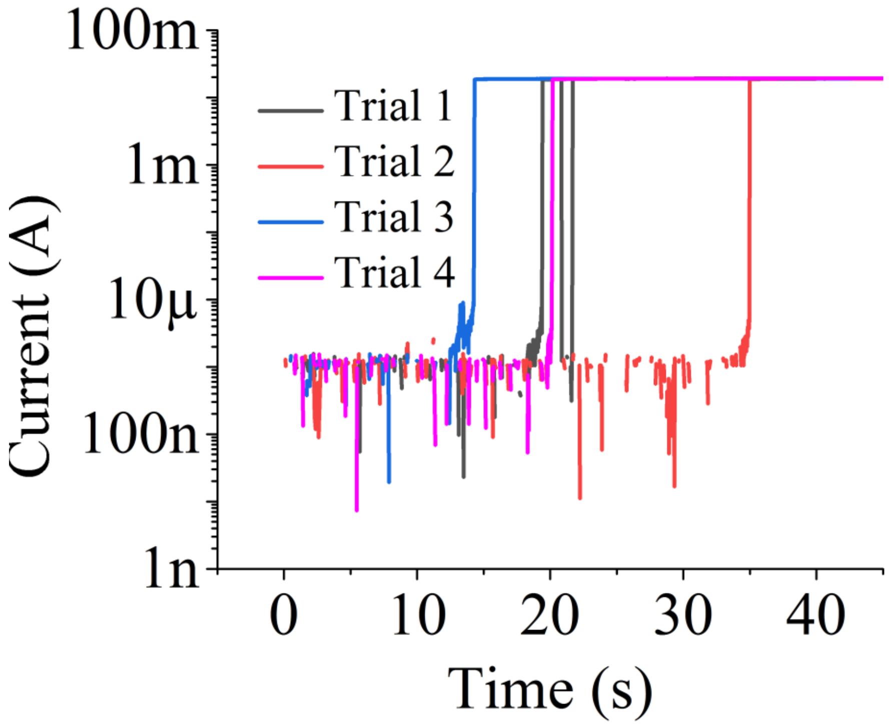

FIG. S39. Concentration = 25 mg/ml

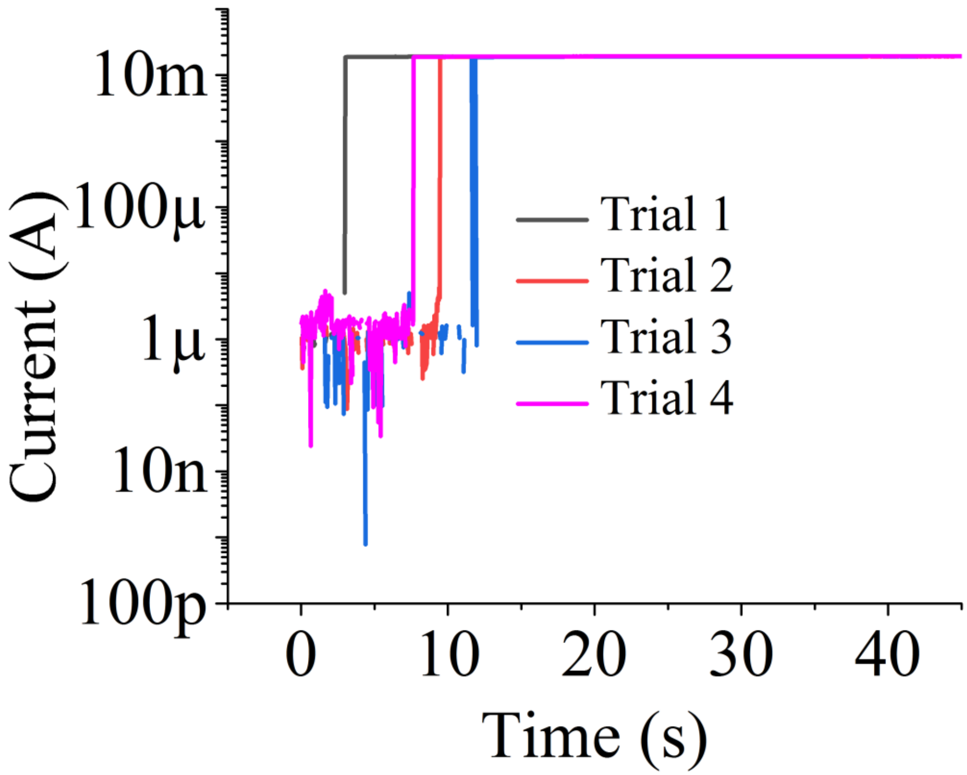

FIG. S40. Concentration = 50 mg/ml

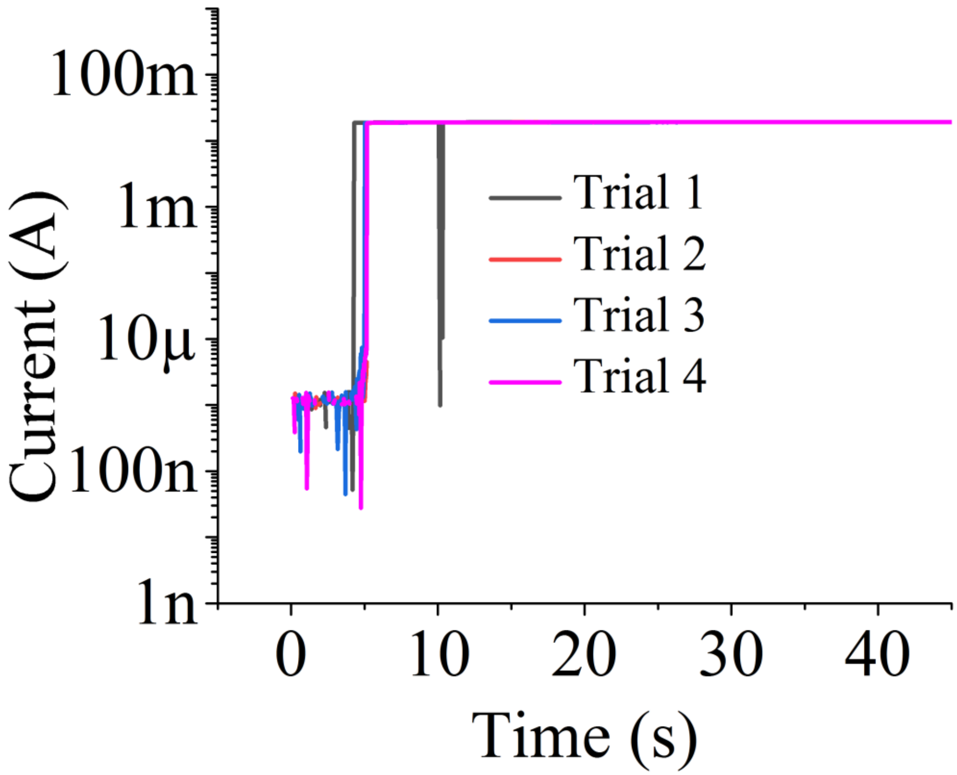

FIG. S41. Concentration = 75 mg/ml

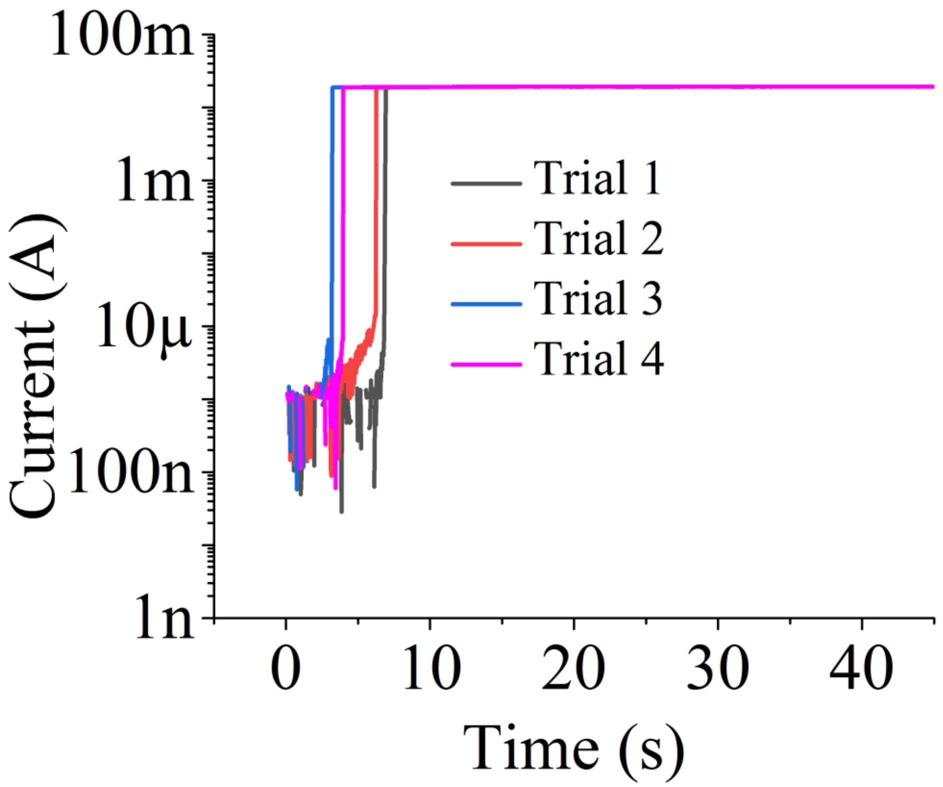

FIG. S42. Concentration = 100 mg/ml

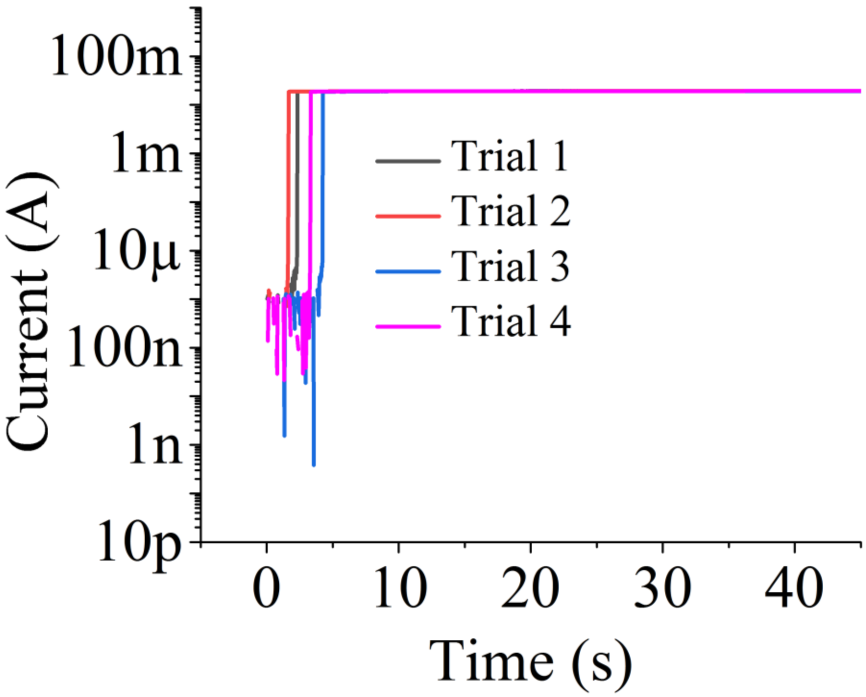

FIG. S43. Concentration = 125 mg/ml

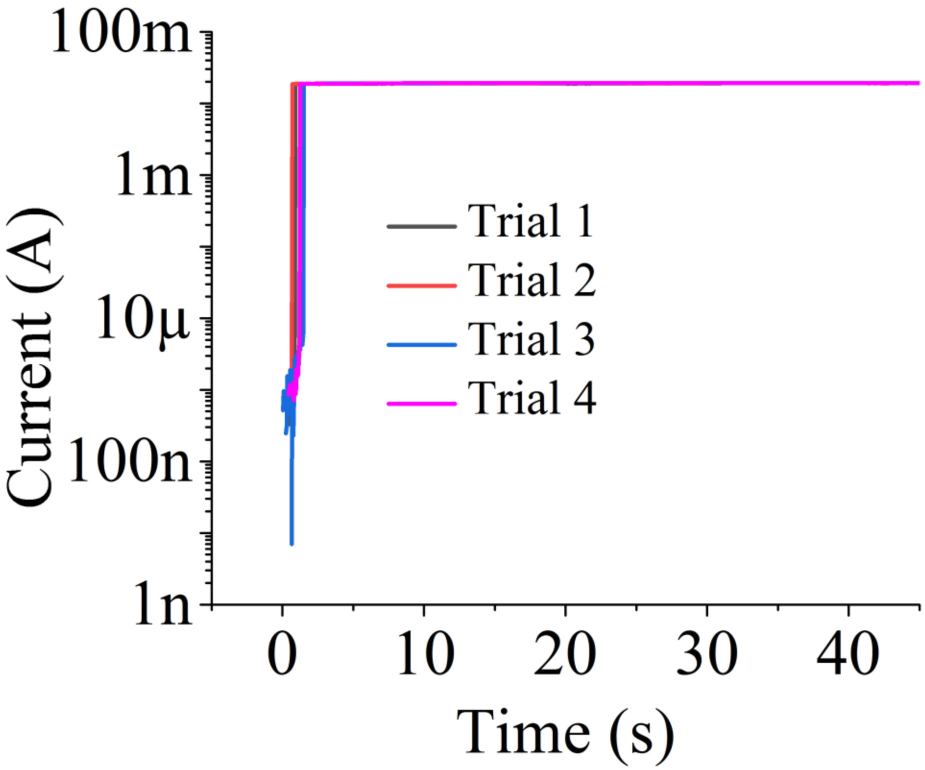

FIG. S44. Concentration = 175 mg/ml

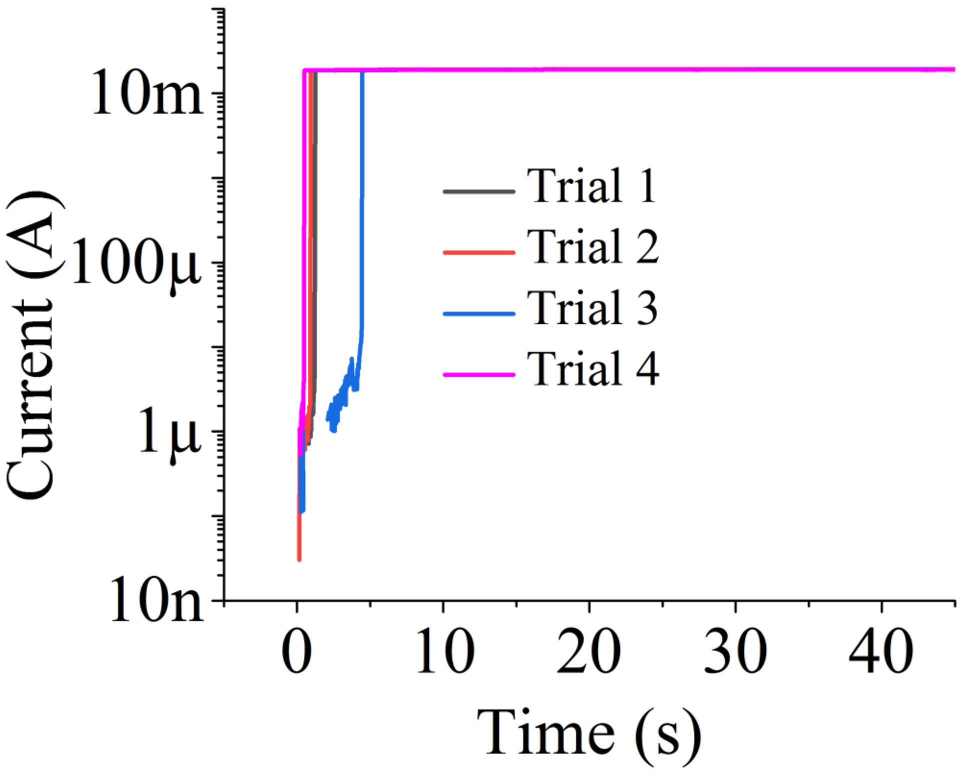

FIG. S45. Concentration = 200 mg/ml

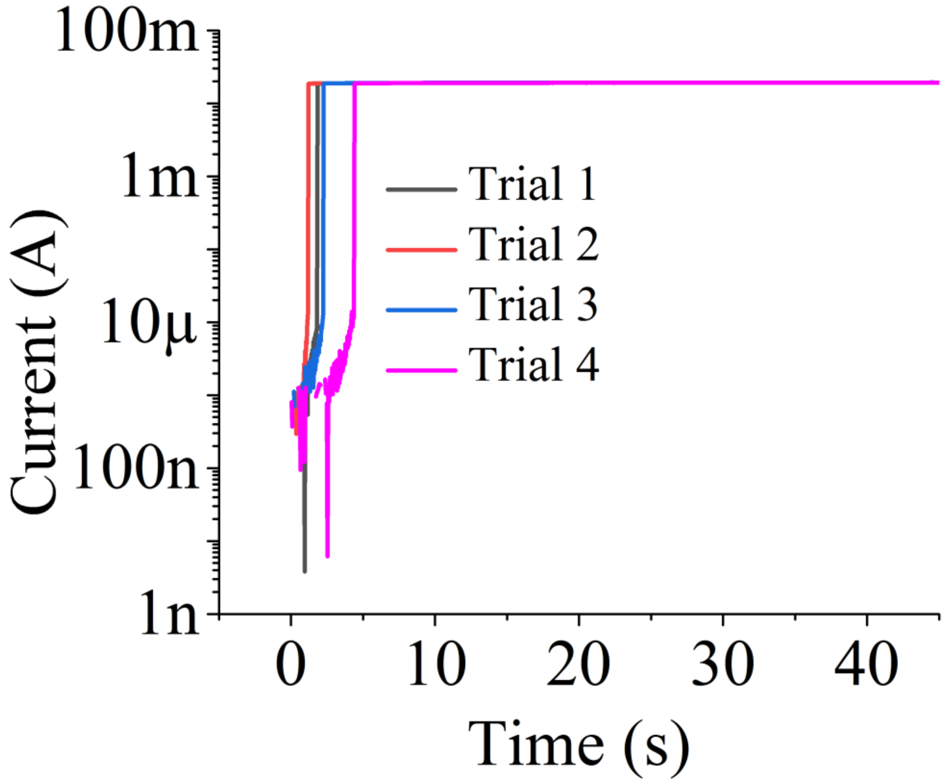

FIG. S46. Concentration = 250 mg/ml

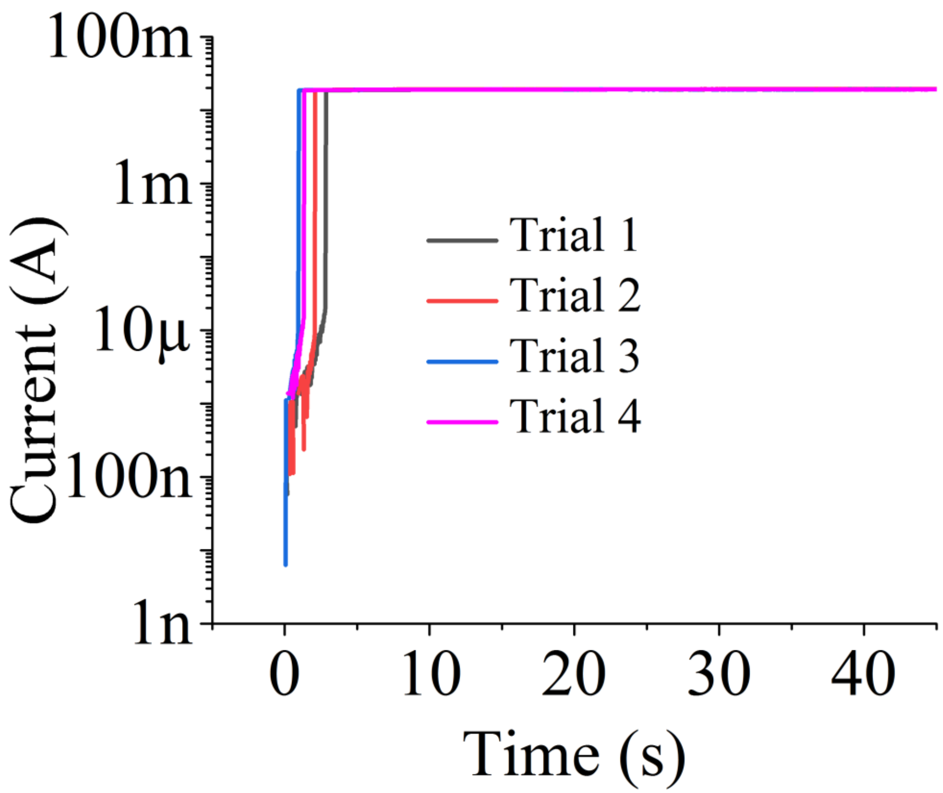

FIG. S47. Concentration = 300 mg/ml

S2. SELF-HEALING WITH GRAPHITE

A. Time Graph

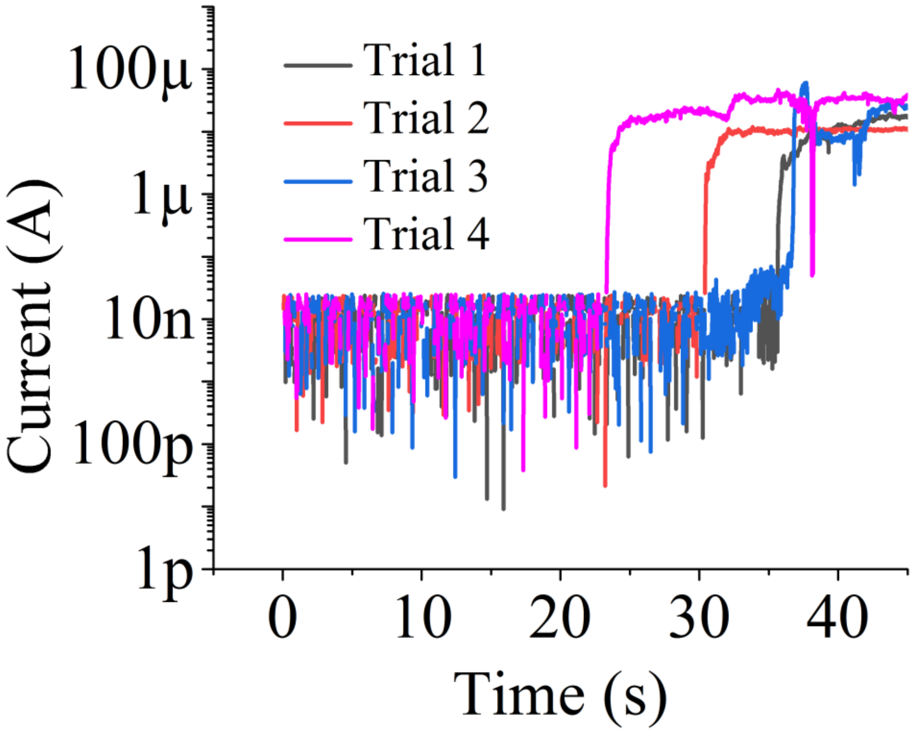

FIG. S48. Concentration = 3 mg/ml

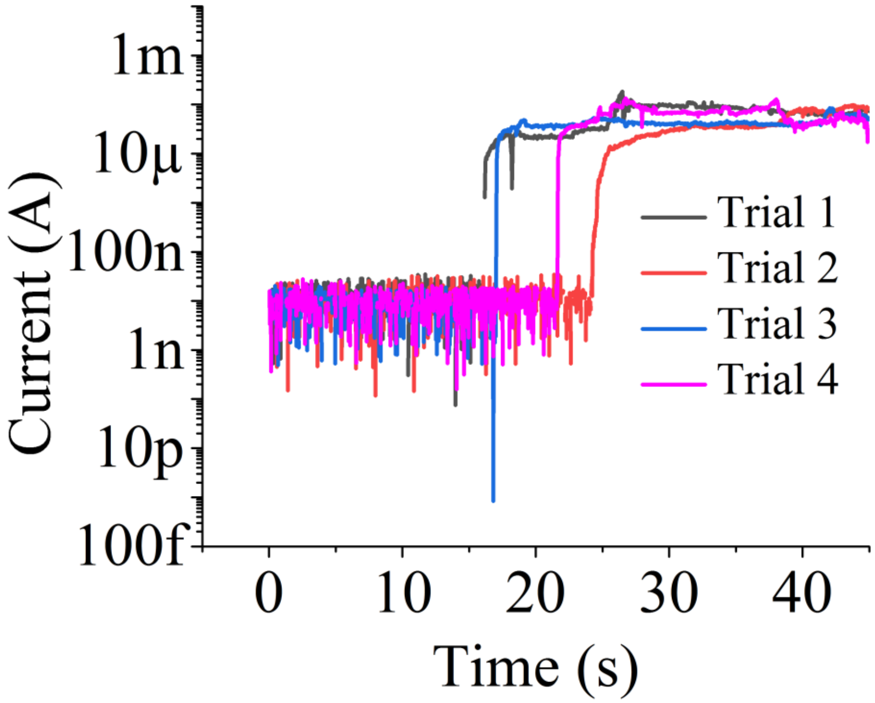

FIG. S49. Concentration = 6 mg/ml

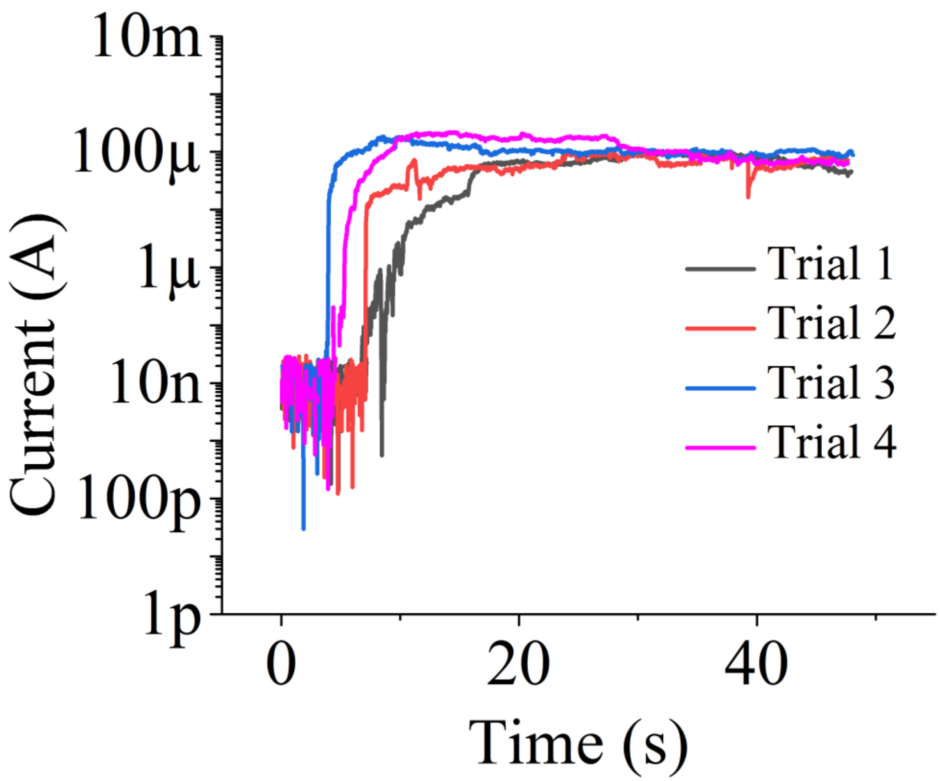

FIG. S50. Concentration = 13 mg/ml

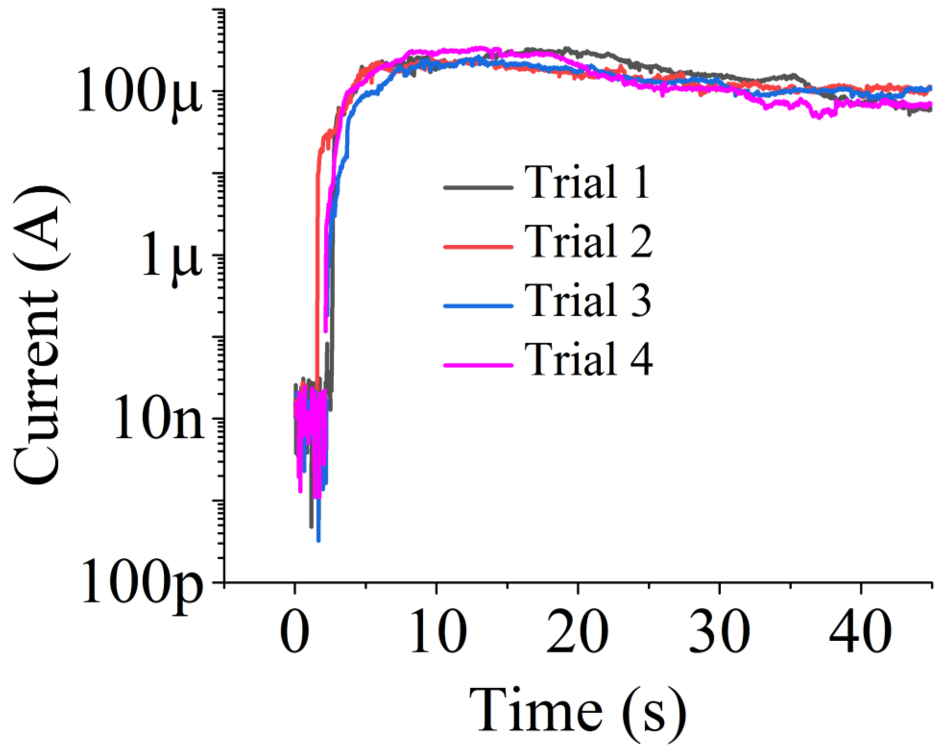

FIG. S51. Concentration = 19 mg/ml

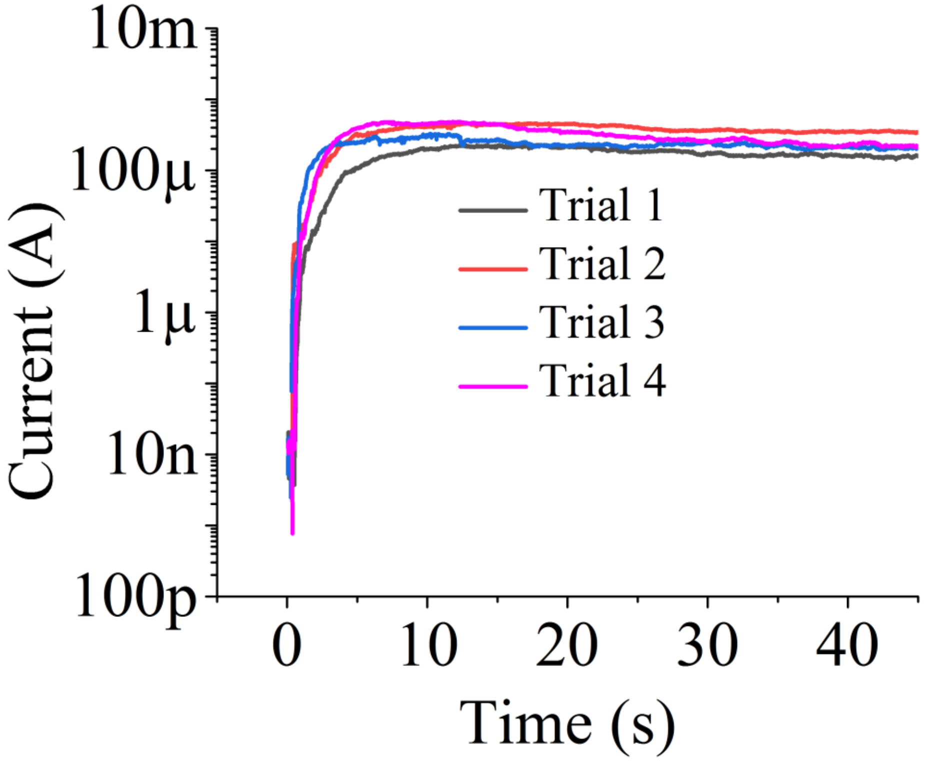

FIG. S52. Concentration = 25 mg/ml

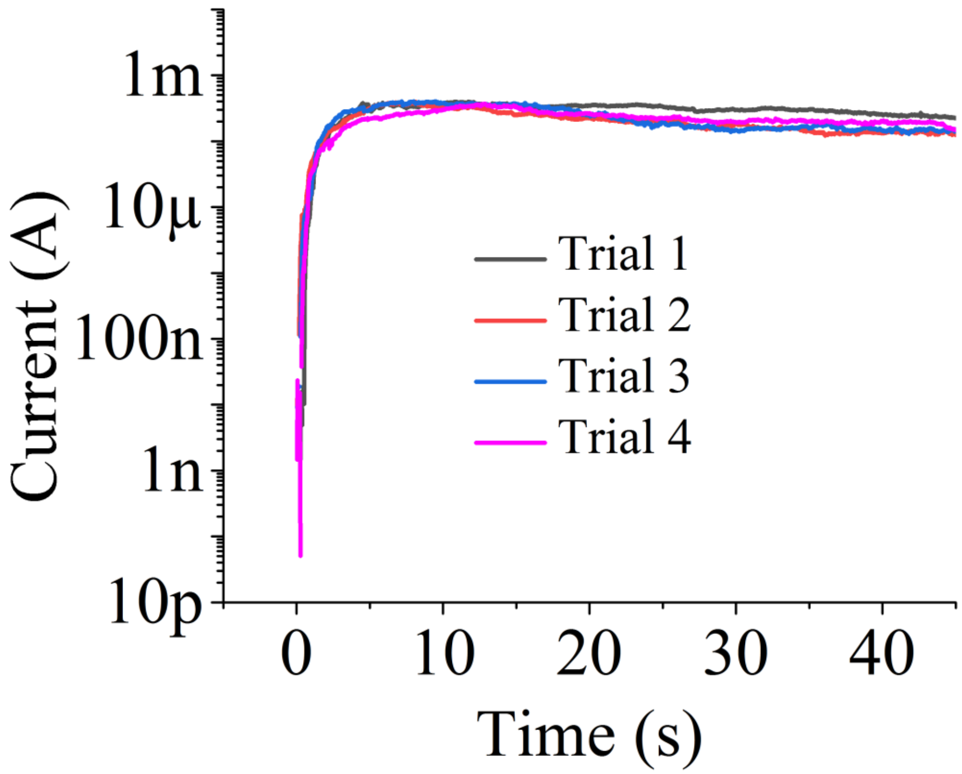

FIG. S53. Concentration = 32 mg/ml

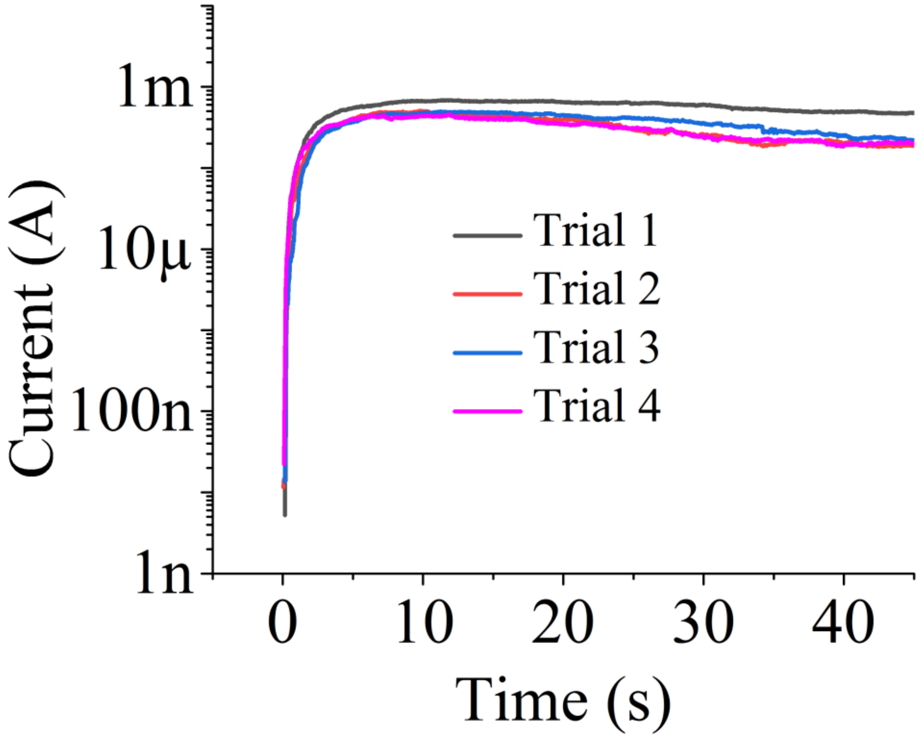

FIG. S54. Concentration = 44 mg/ml

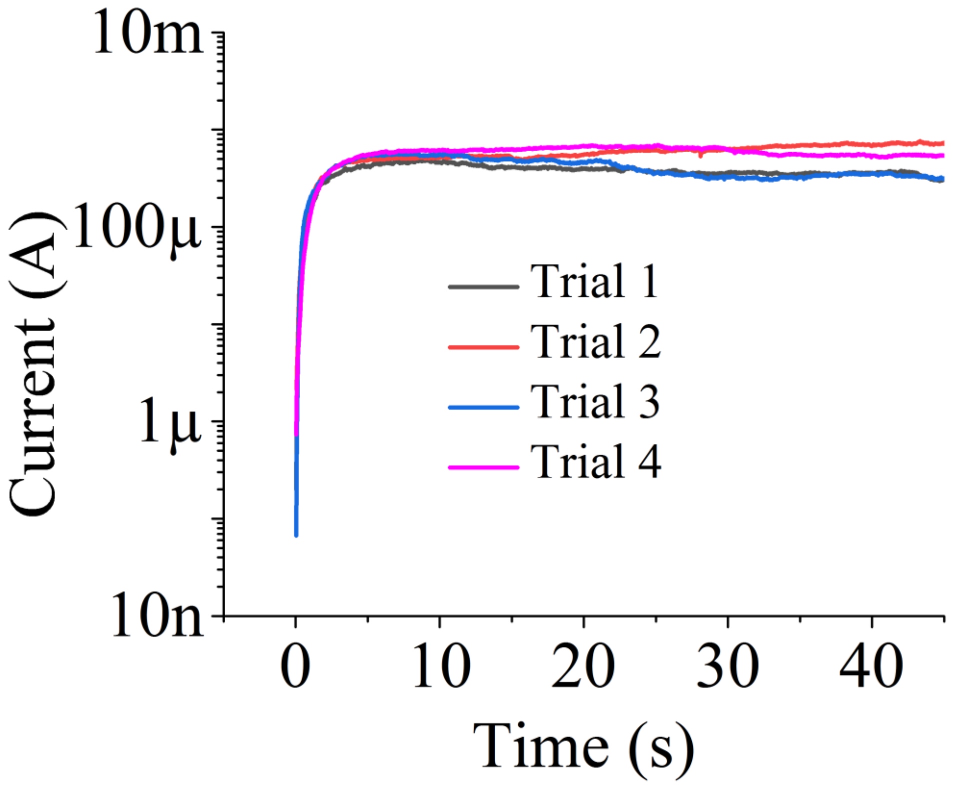

FIG. S55. Concentration = 51 mg/ml

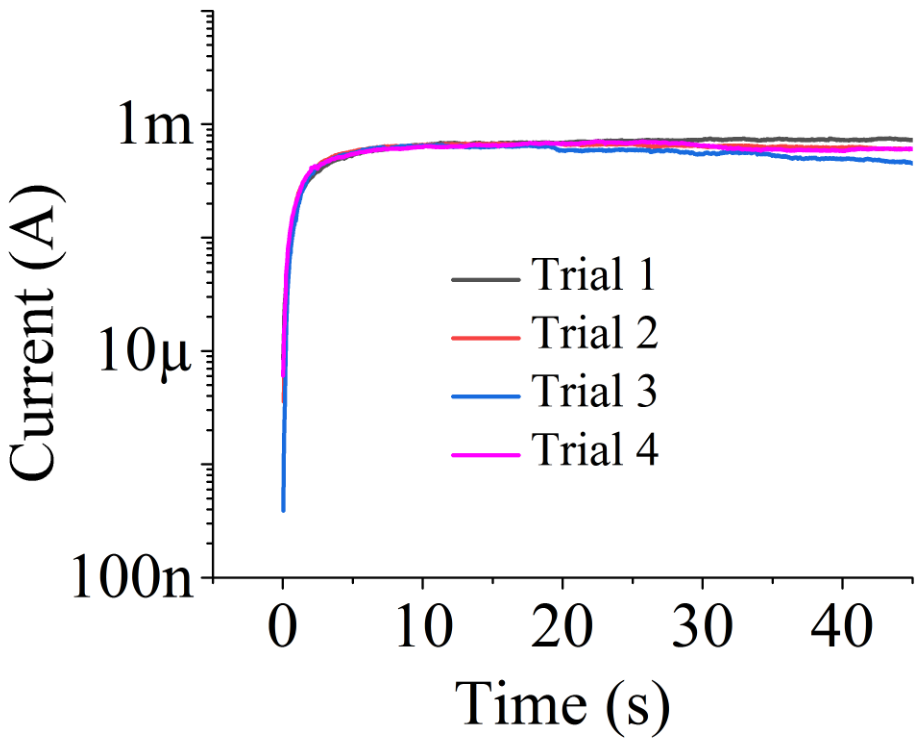

FIG. S56. Concentration = 63 mg/ml

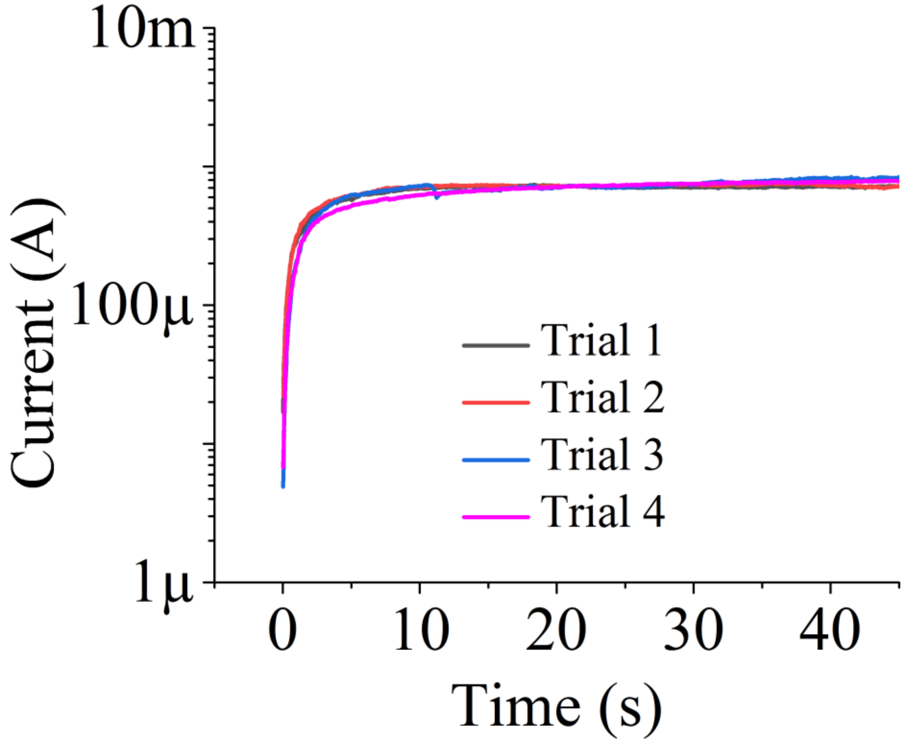

FIG. S57. Concentration = 76 mg/ml

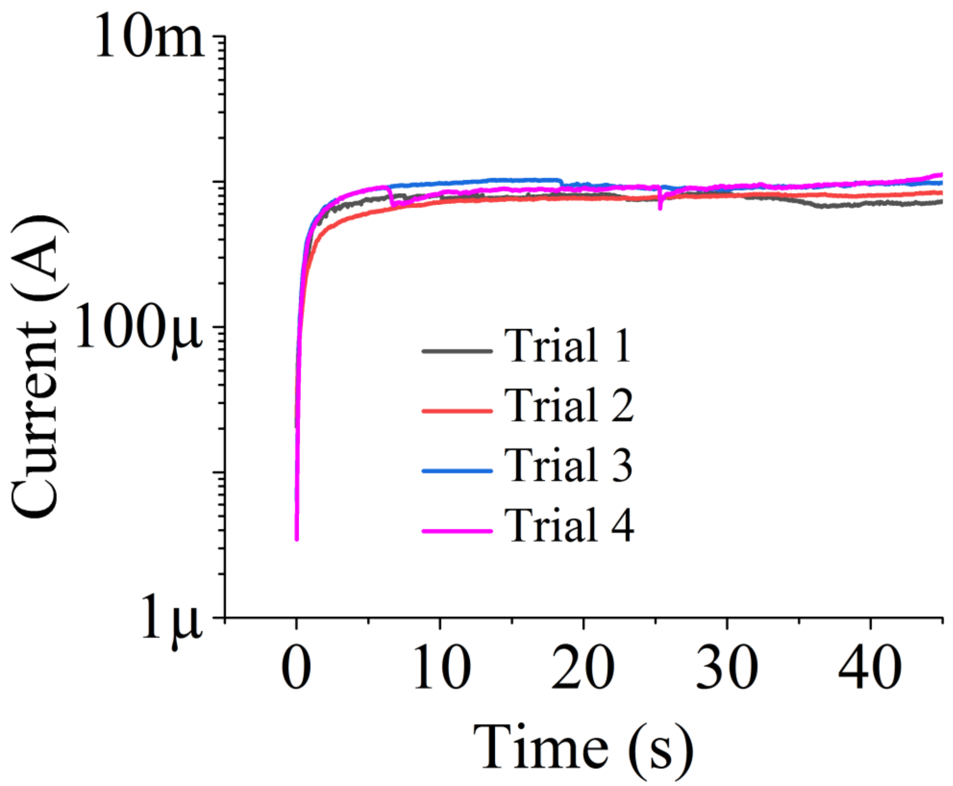

FIG. S58. Concentration = 89 mg/ml

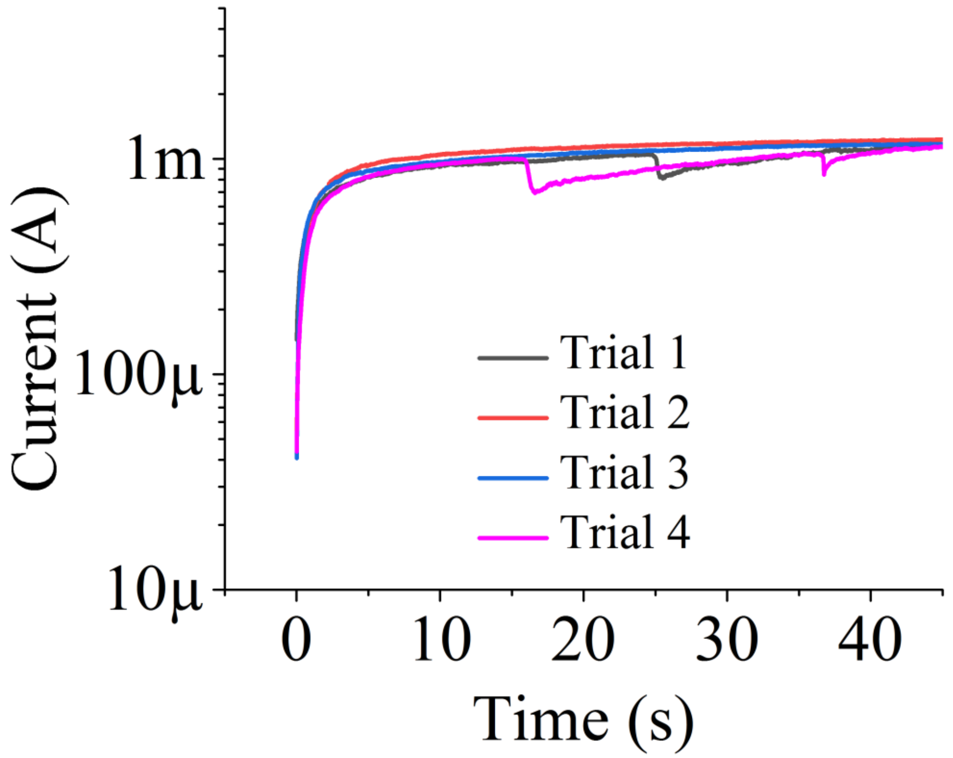

FIG. S59. Concentration = 101 mg/ml

B. Resistance

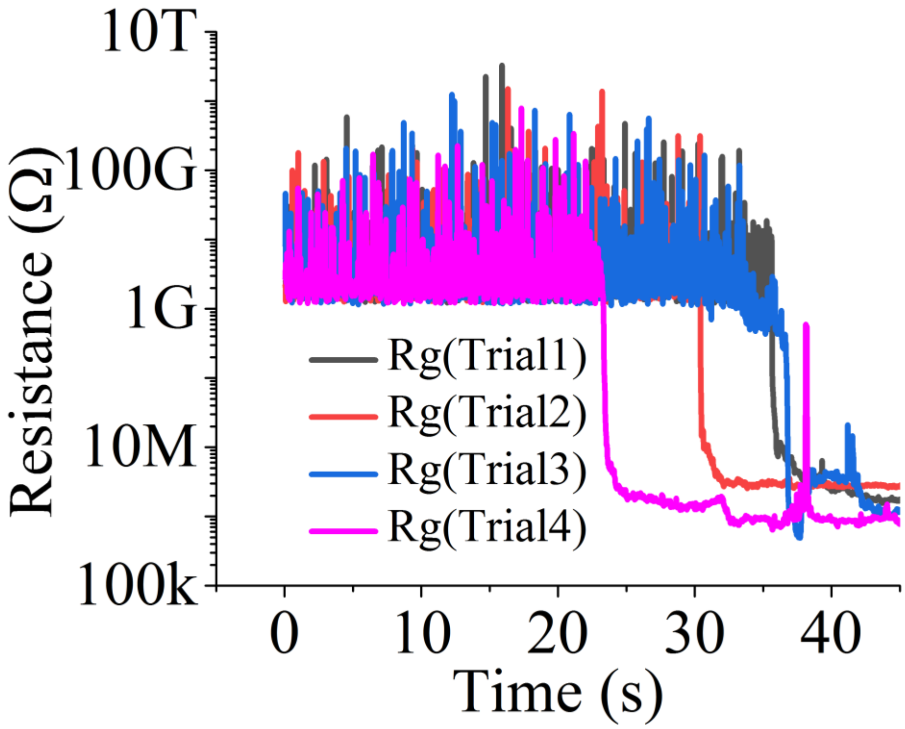

FIG. S60. Concentration = 3 mg/ml

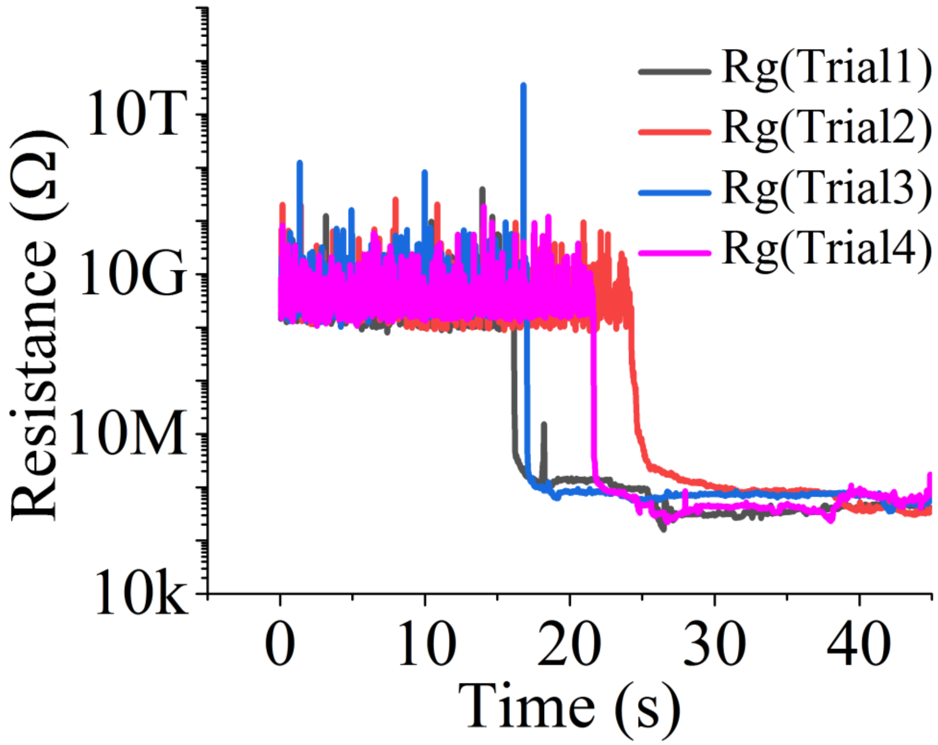

FIG. S61. Concentration = 6 mg/ml

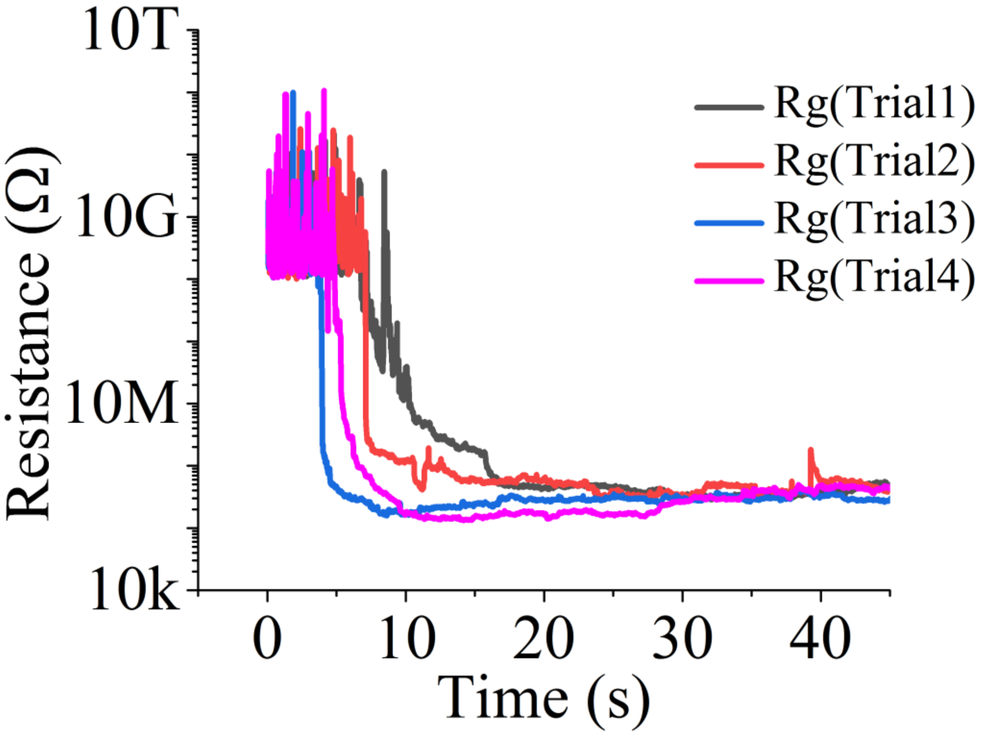

FIG. S62. Concentration = 13 mg/ml

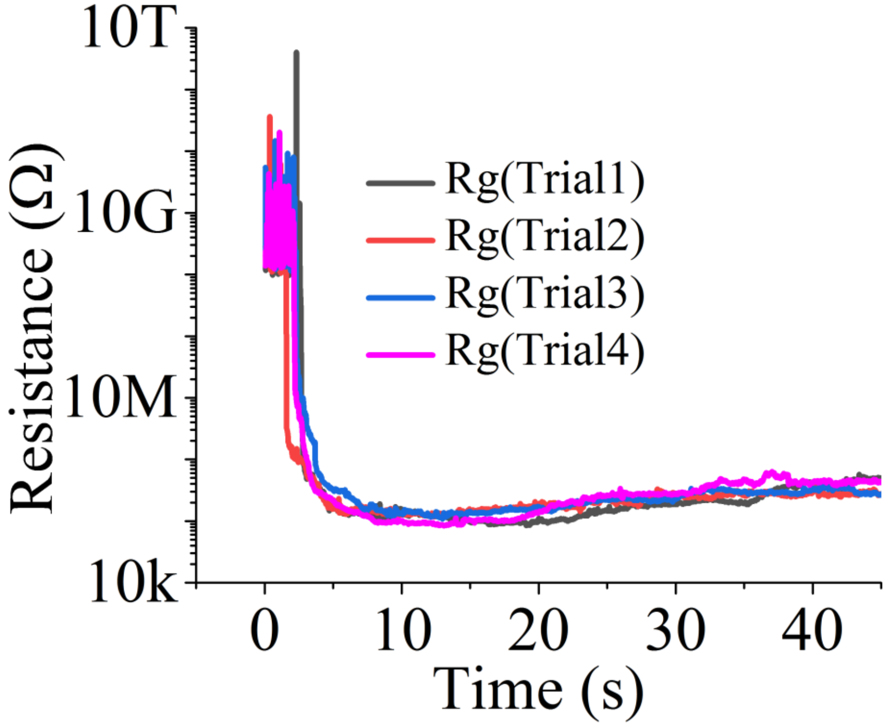

FIG. S63. Concentration = 19 mg/ml

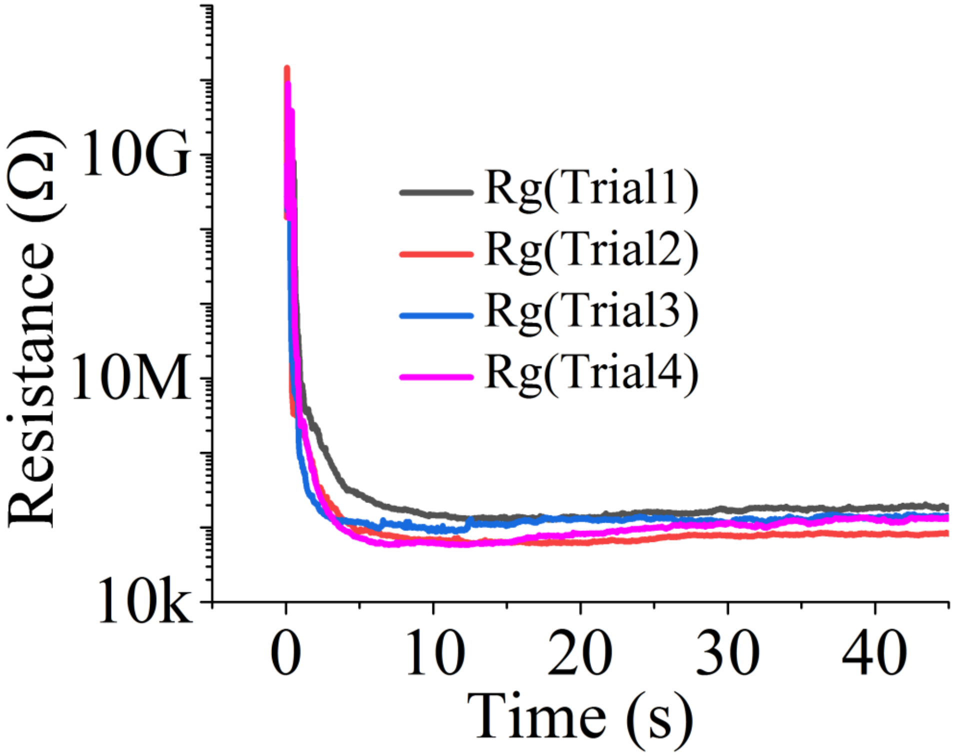

FIG. S64. Concentration = 25 mg/ml

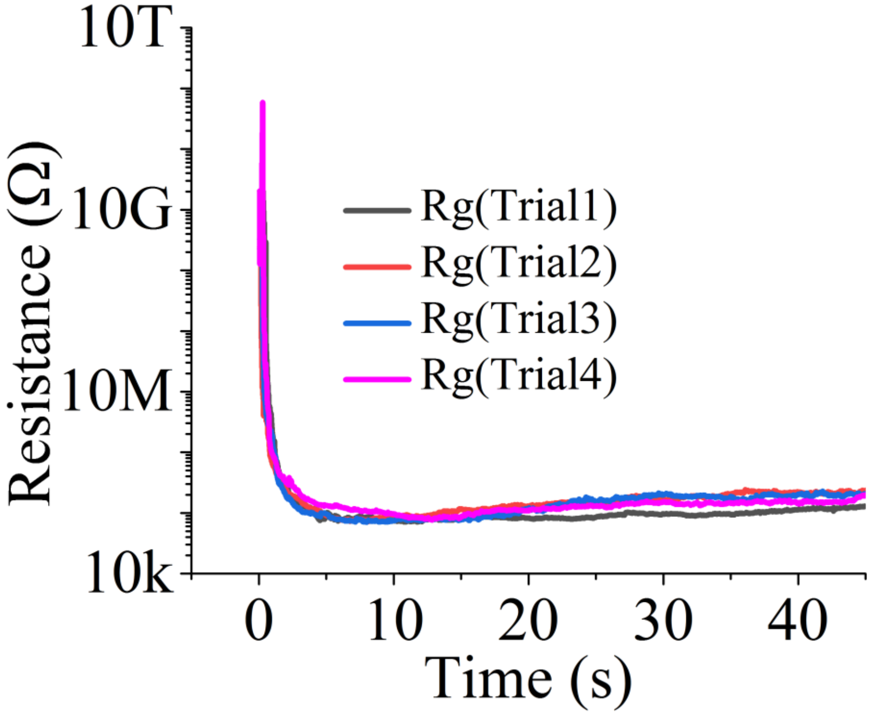

FIG. S65. Concentration =32 mg/ml

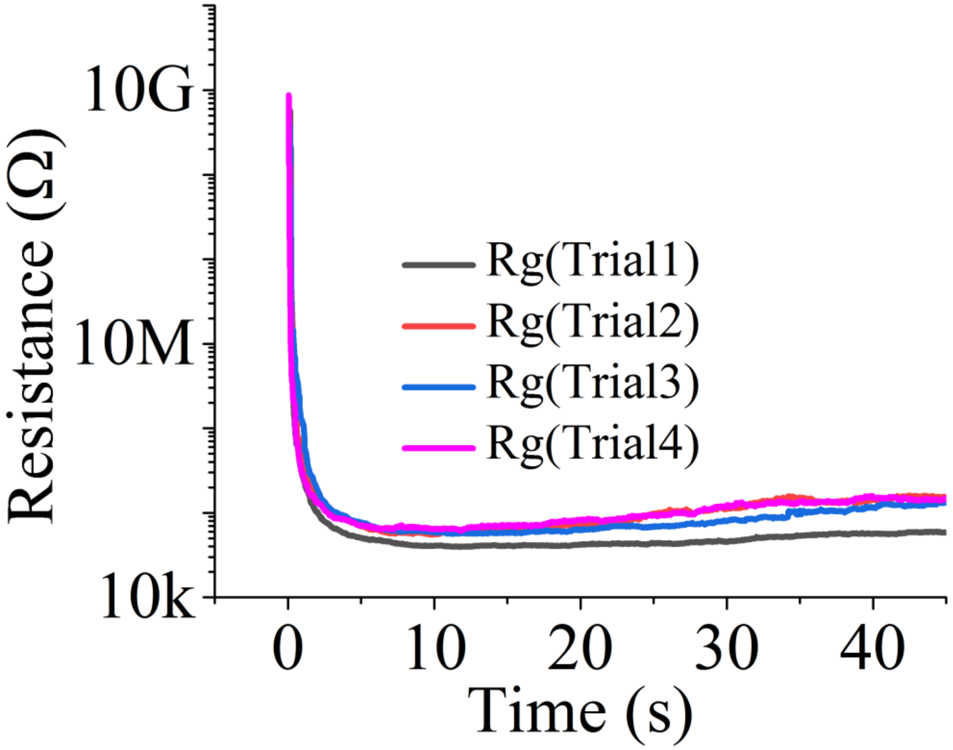

FIG. S66. Concentration = 44 mg/ml

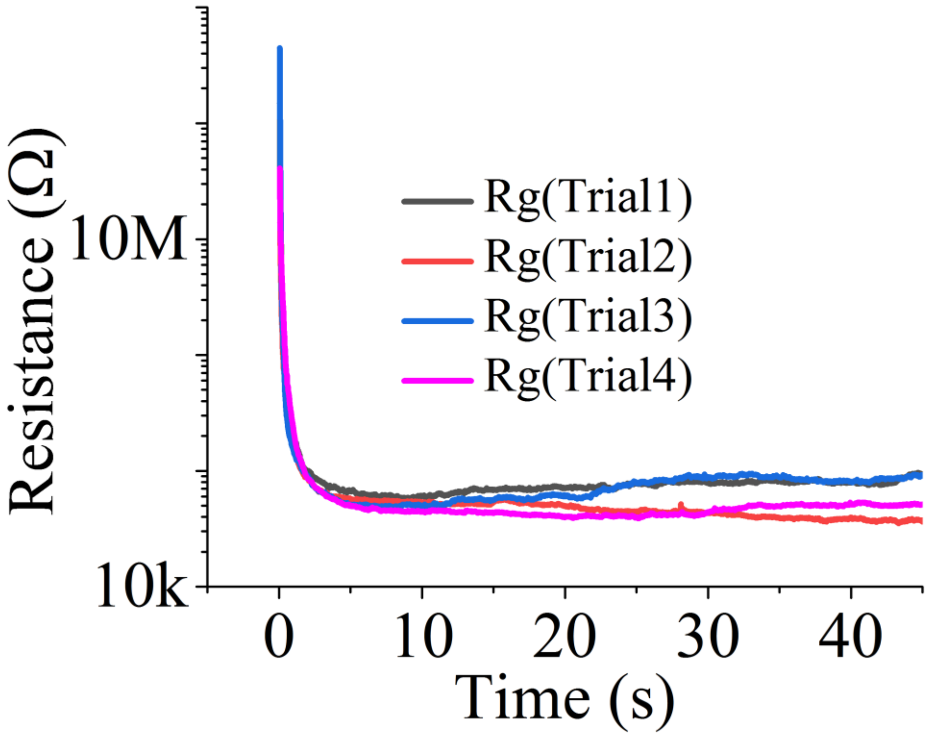

FIG. S67. Concentration = 51 mg/ml

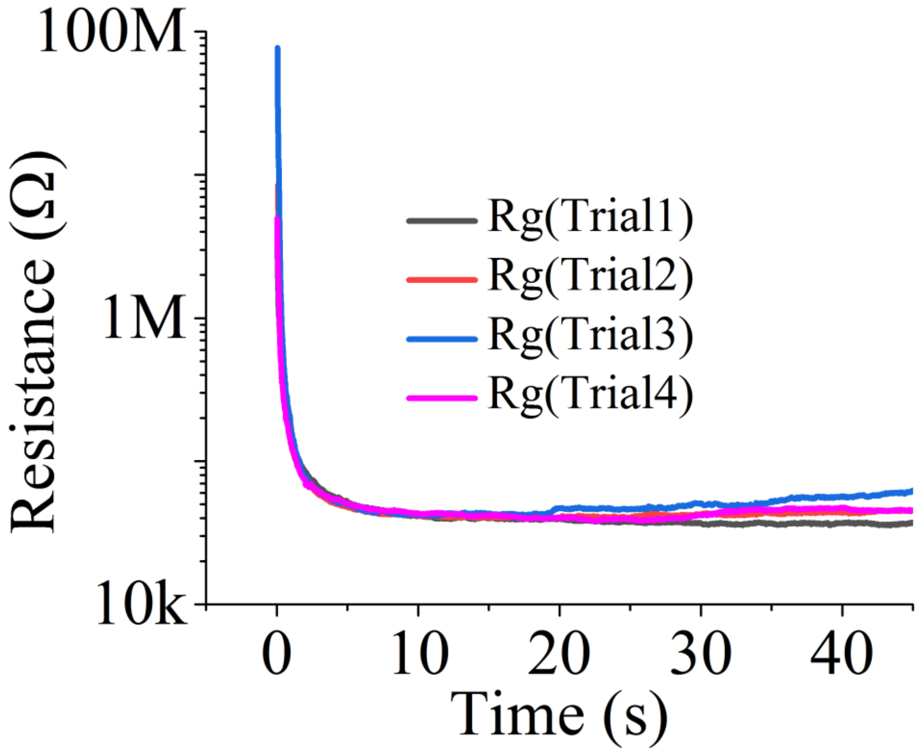

FIG. S68. Concentration = 63 mg/ml

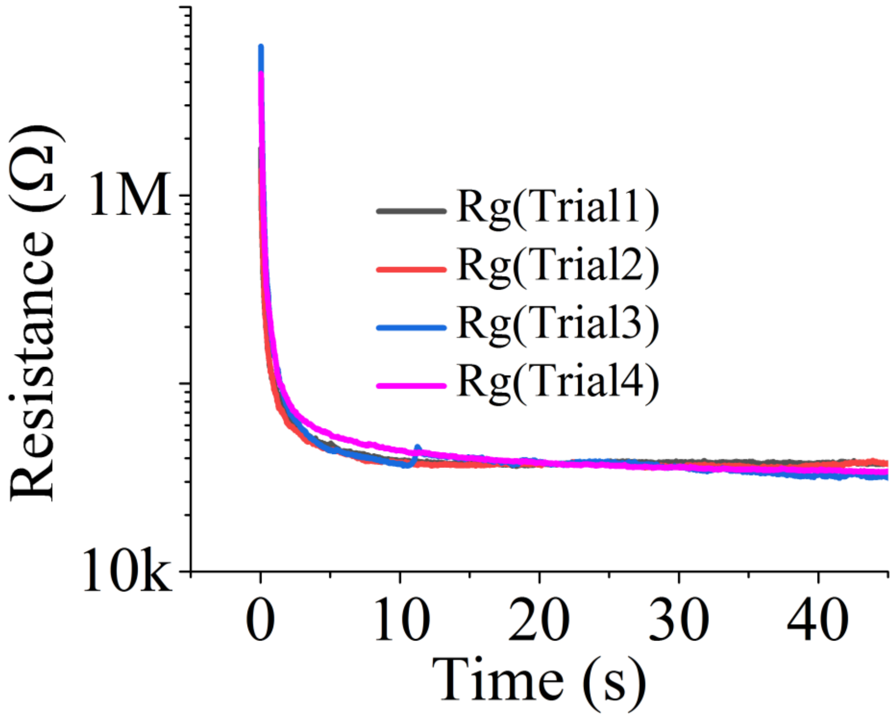

FIG. S69. Concentration = 76 mg/ml

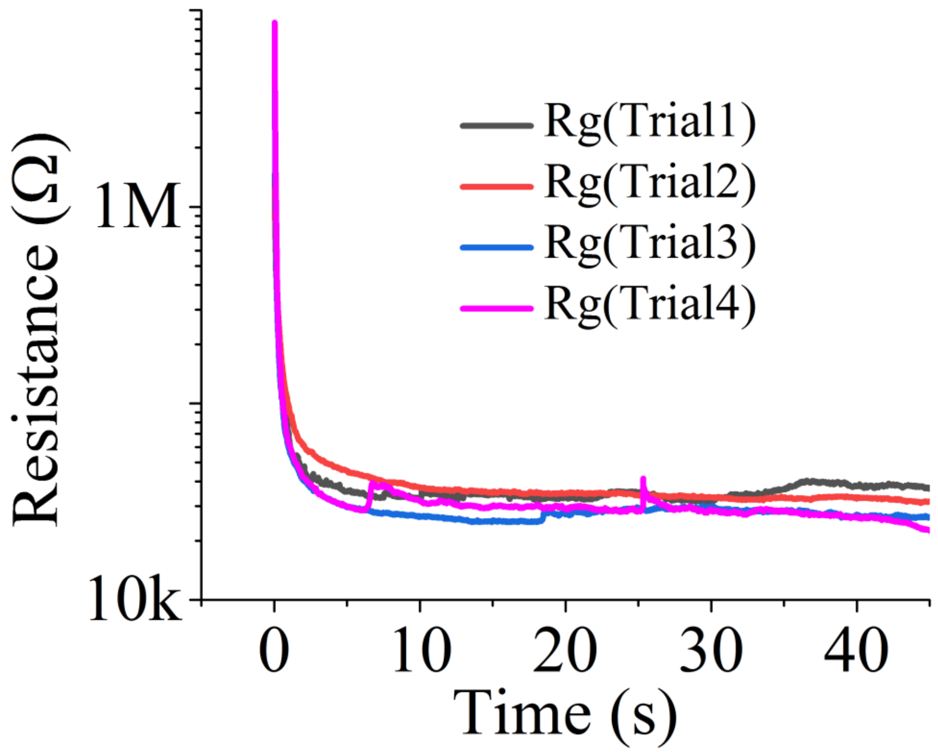

FIG. S70. Concentration = 89 mg/ml

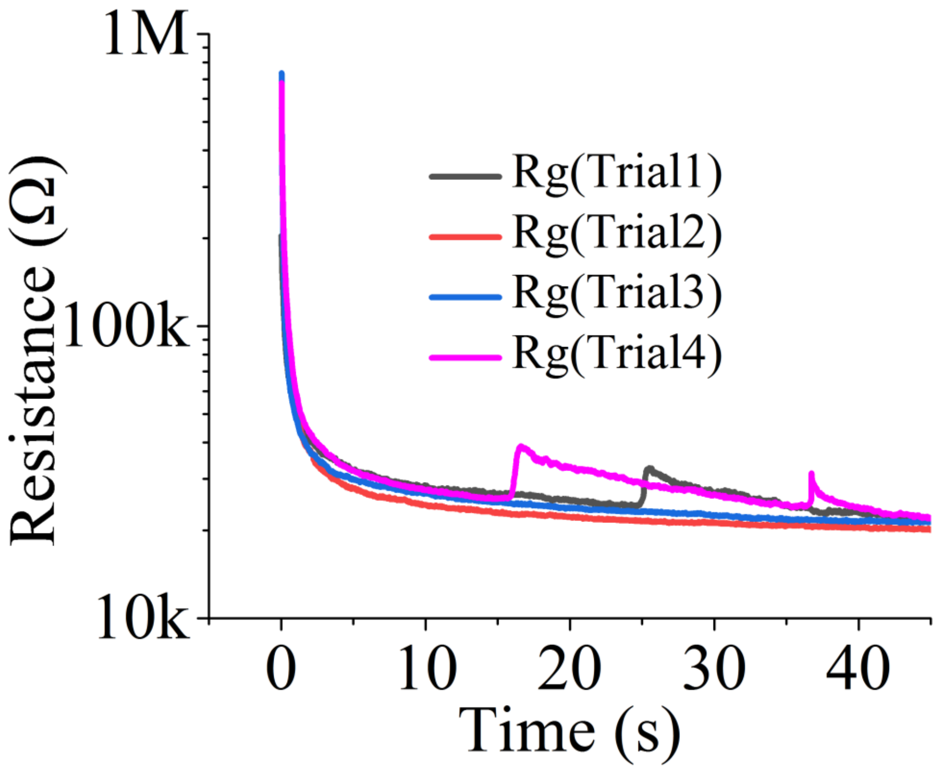

FIG. S71. Concentration = 101 mg/ml

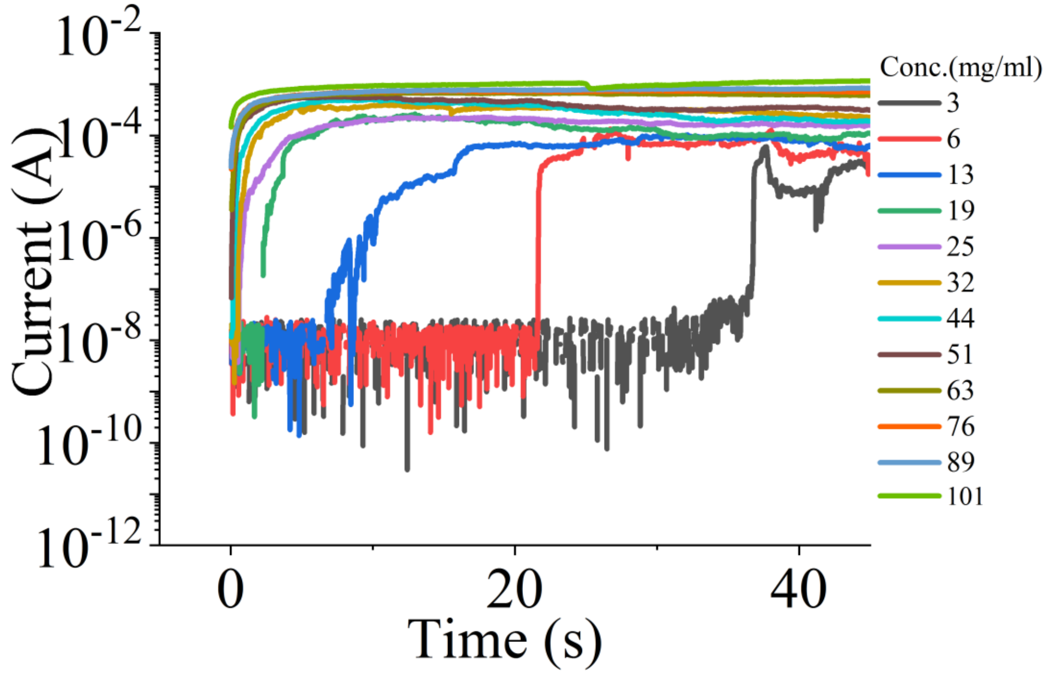

FIG. S72. Cumulative current vs time plot for concentrations

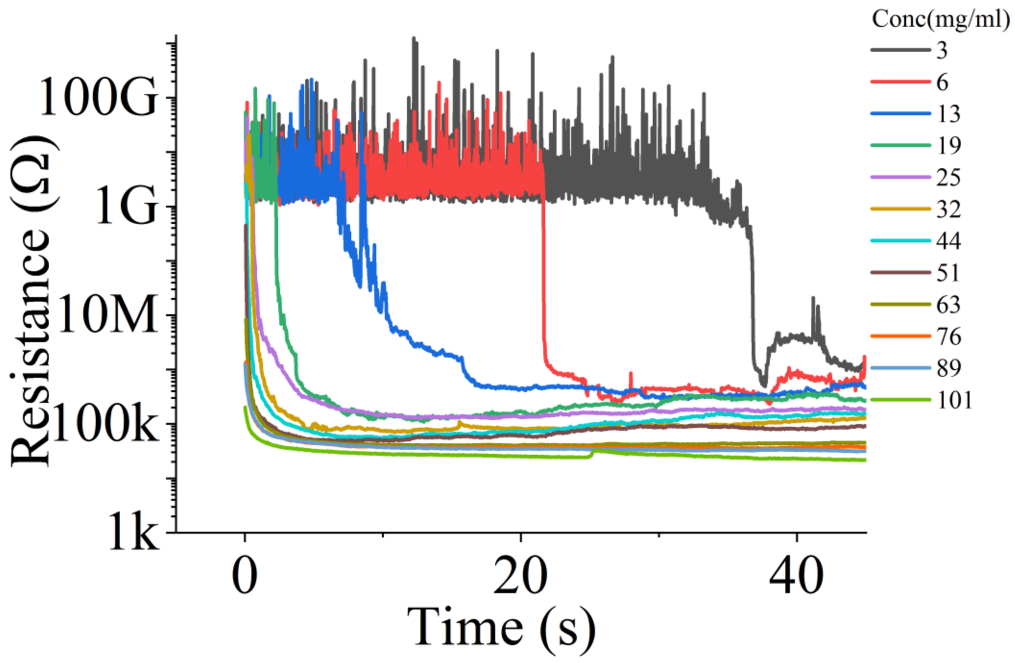

FIG. S73. Cumulative Resistance vs time plot for concentrations

S3. Graphite Bridge SEM Images

There is no sintering in the case of self-healing with graphite dispersions. Since the bridges are not as permanent as in the case of copper, rinsing of the oil with isopropyl alcohol before SEM is not done as it would damage the bridge. The oil residue results in charging and is seen as the bright regions in the images.

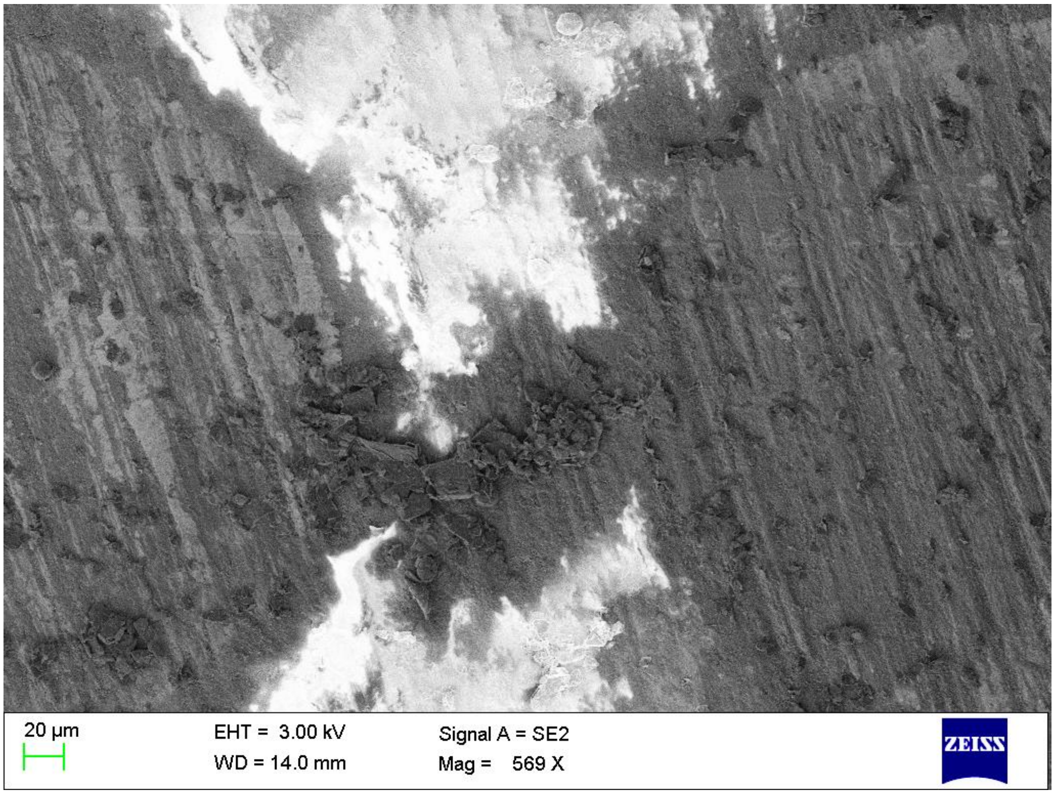

FIG. S74. Graphite bridge SEM 1

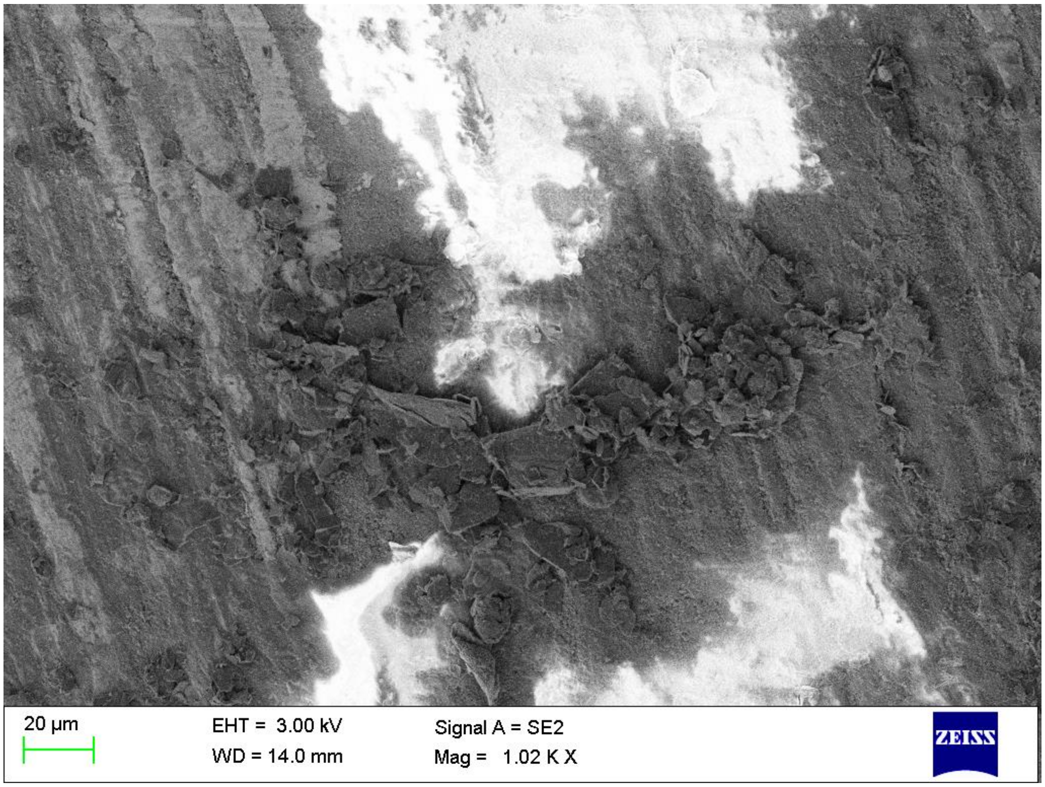

FIG. S75. Graphite bridge SEM 2

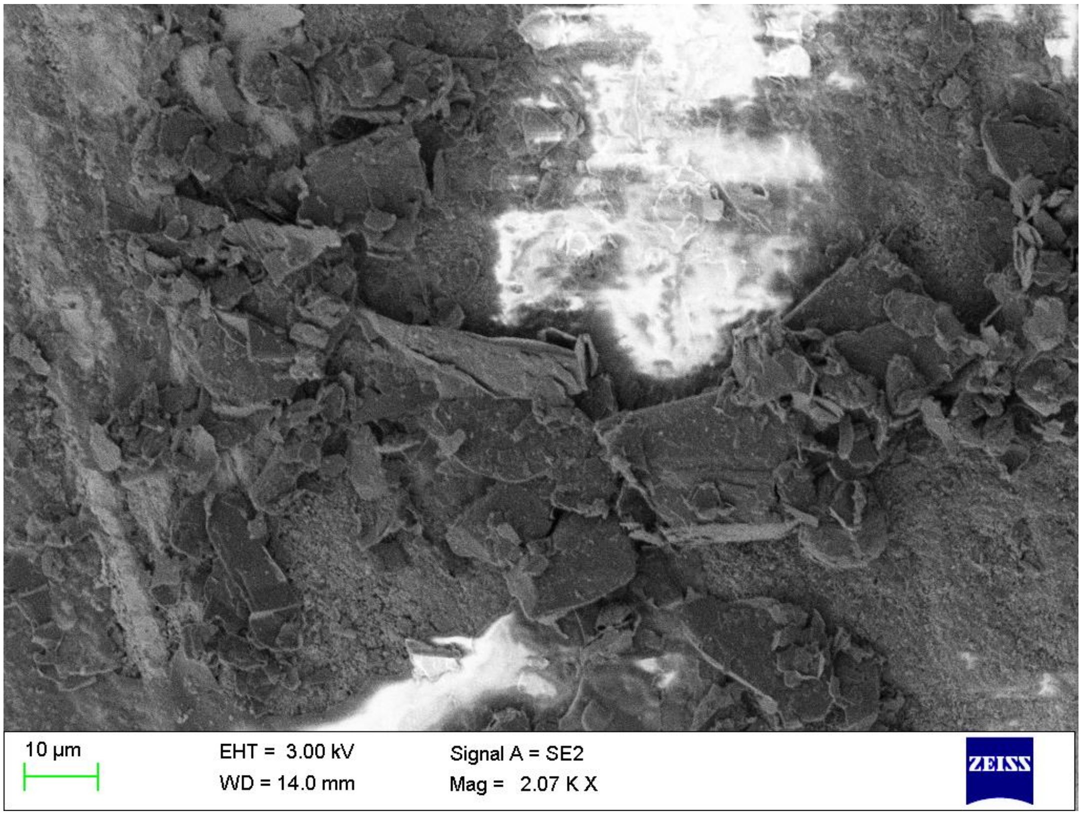

FIG. S76. Graphite bridge left side SEM

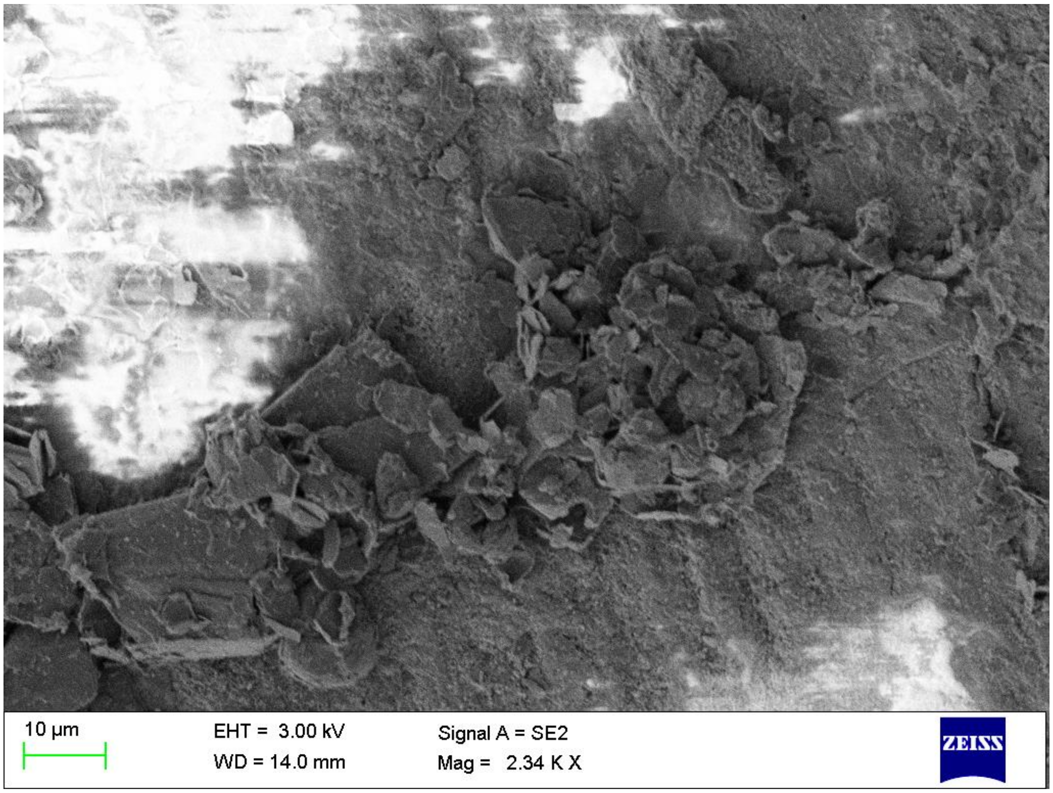

FIG. S77. Graphite bridge right side SEM

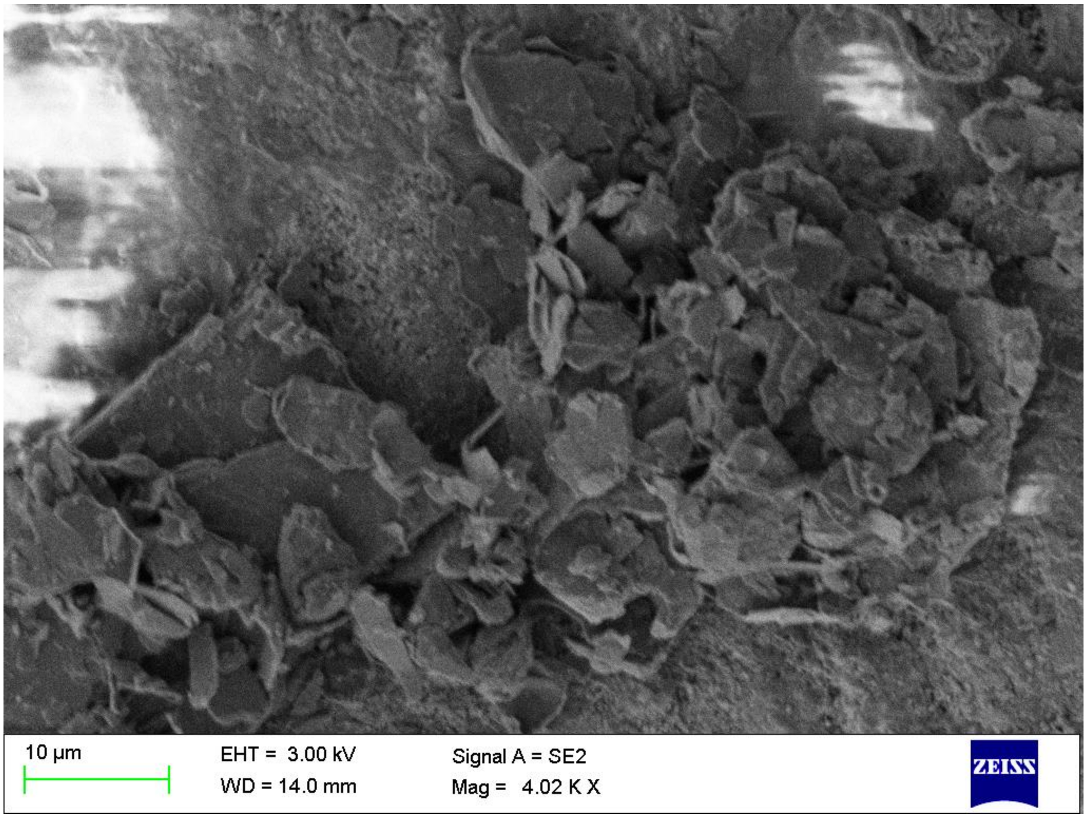

FIG. S78. Graphite bridge right side SEM more zoom

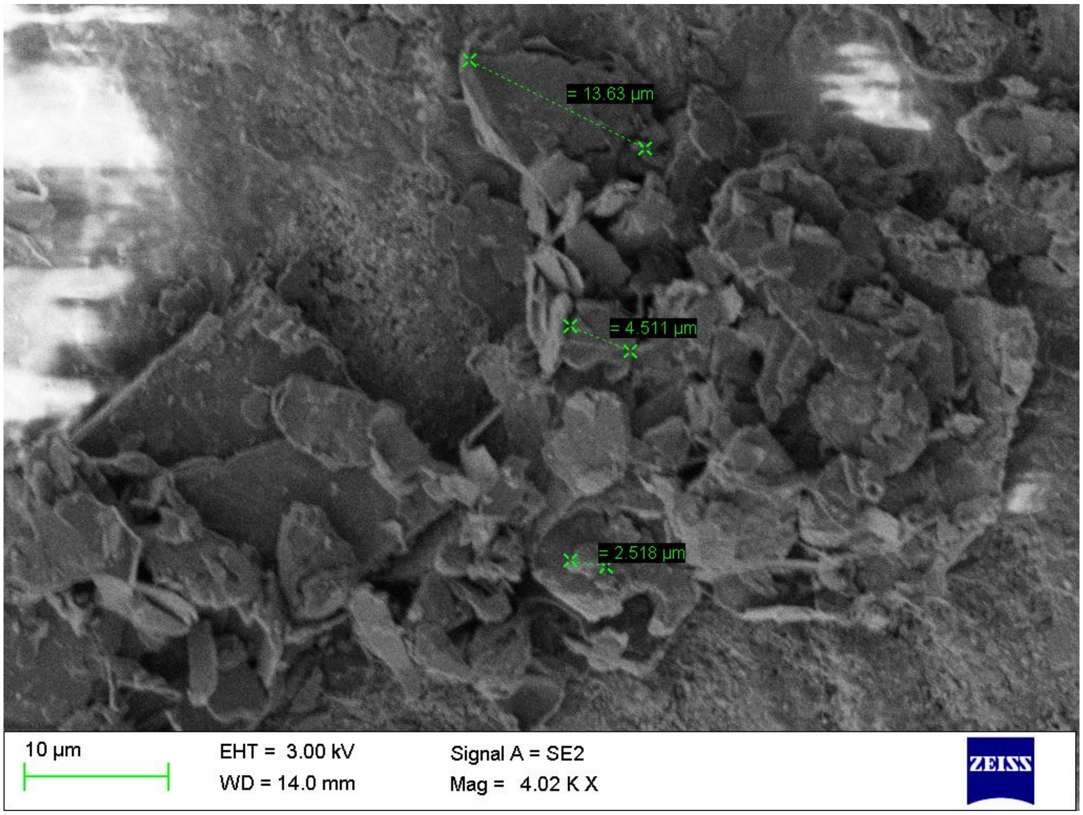

FIG. S79. Graphite bridge right side SEM more zoom with particle size

S4. X-Ray Photoelectron Spectroscopy (XPS) of COPPER PARTICLES

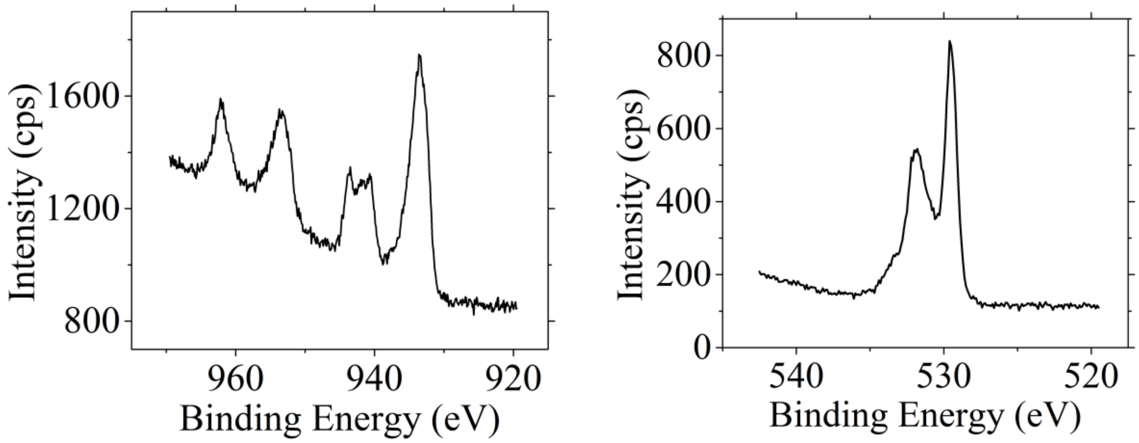

Fig. S80. X-Ray Photoelectron Spectroscopy (XPS) of copper particles. The particles show clear peaks of Oxygen and Copper. This can be compared to reference https://srdata.nist.gov/xps/EnergyTypeValSrch.aspx.

S5. Energy-Dispersive X-Ray Spectroscopy (EDX) SEM of the HEAL

EDX SEM of the heal. Since all experiments (starting from dispersion preparation to healing) are carried at close to ambient conditions, the presence of oxide over the metallic particles is unavoidable. After sintering, during SEM measurements it is expected that the surface still have oxide (as (i) the sintering only results in a necking at the point of contact (ii) and there is sufficient delay between the healing and the EDAX characterization). Yet, since the heal shows excellent conductivity after sintering - it is speculated that inside the sintered neck the oxide should have broken down.

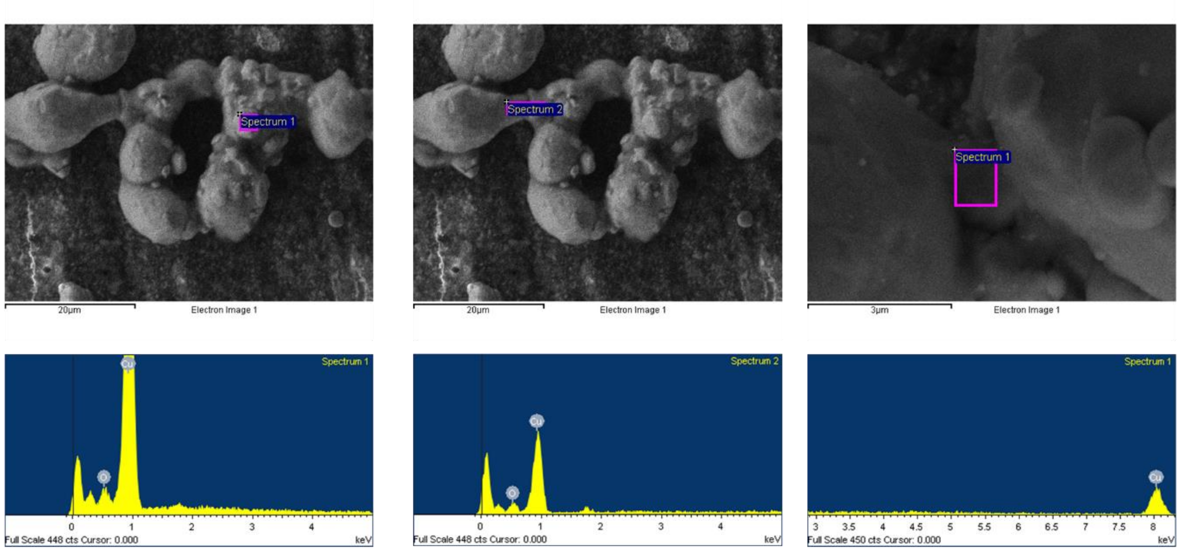

Cu: atomic % = 74.2 , weight % =91.95
O: atomic % = 25.8, weight % =8.05

Cu: atomic % = 81.07 , weight % = 94.45
O: atomic % = 18.93, weight % = 5.55

Cu: atomic % = 21.54, weight % = 93.53
O: atomic % = 78.46, weight % = 6.47

Fig. S81. Energy dispersive X-Ray Spectroscopy of the surface of the heal

S6. CROSSTALK EXPERIMENTAL SETUP

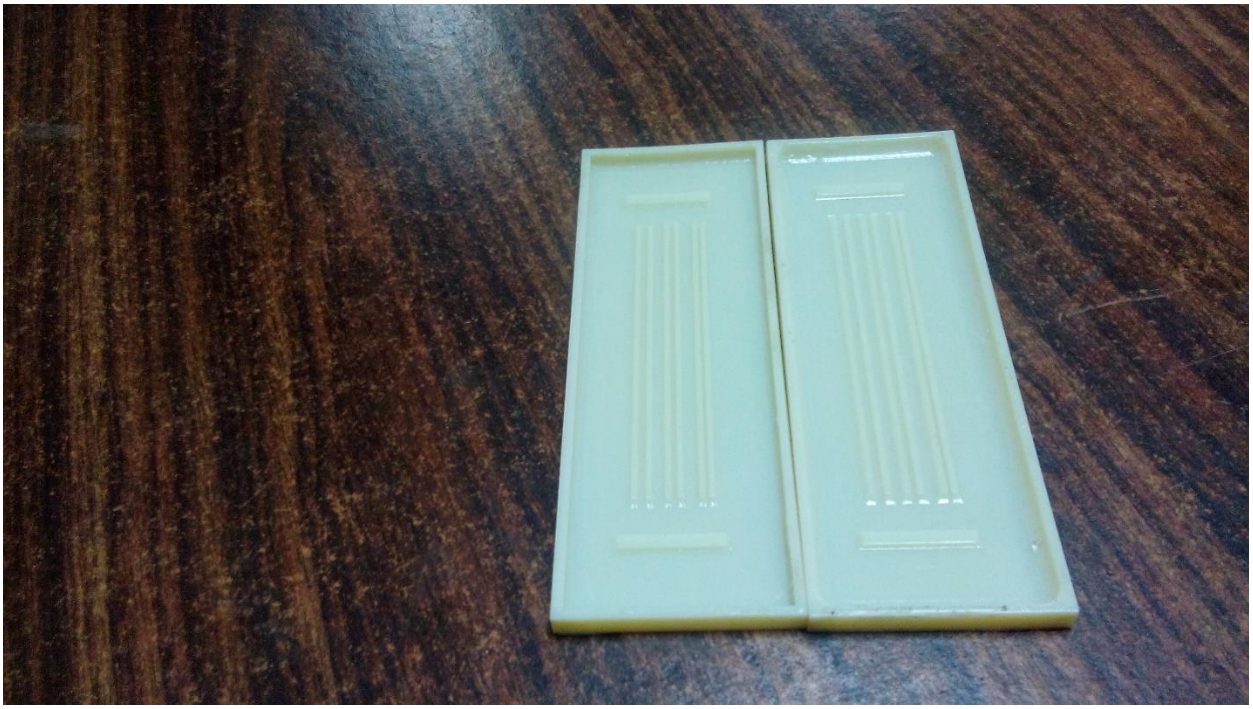

FIG. S82. Plastic mold for PDMS Mask

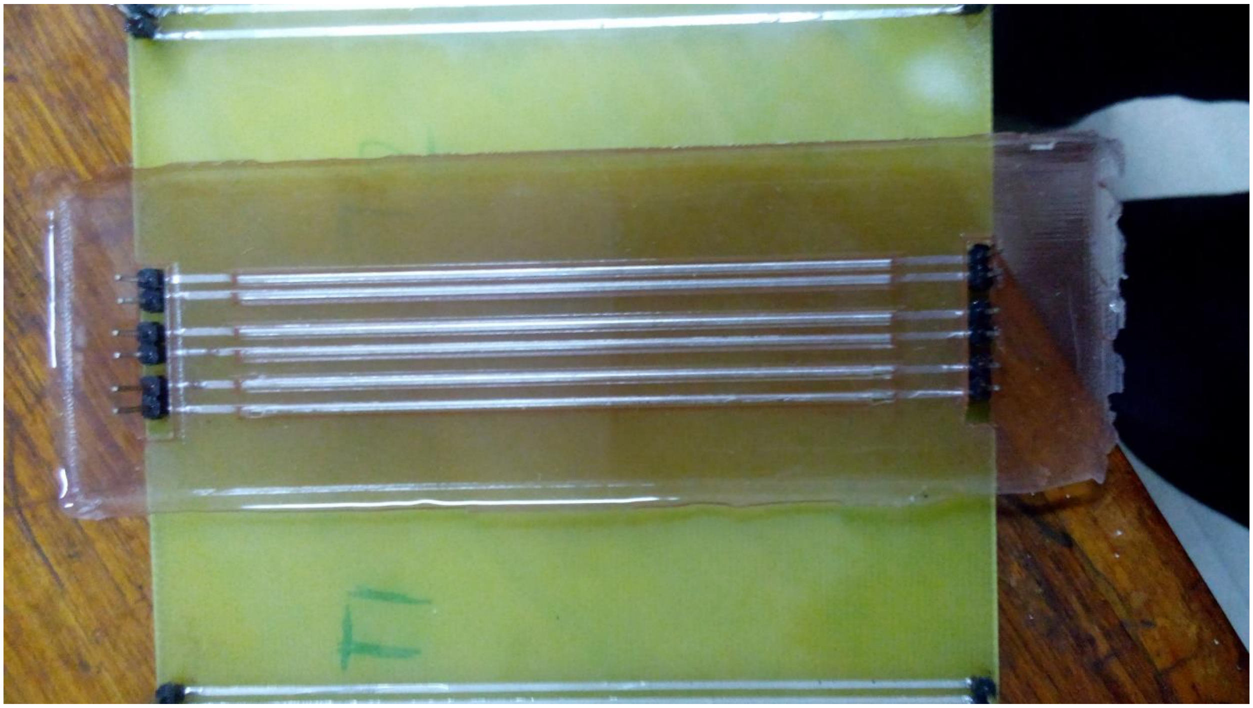

FIG. S83. Copper track with PDMS

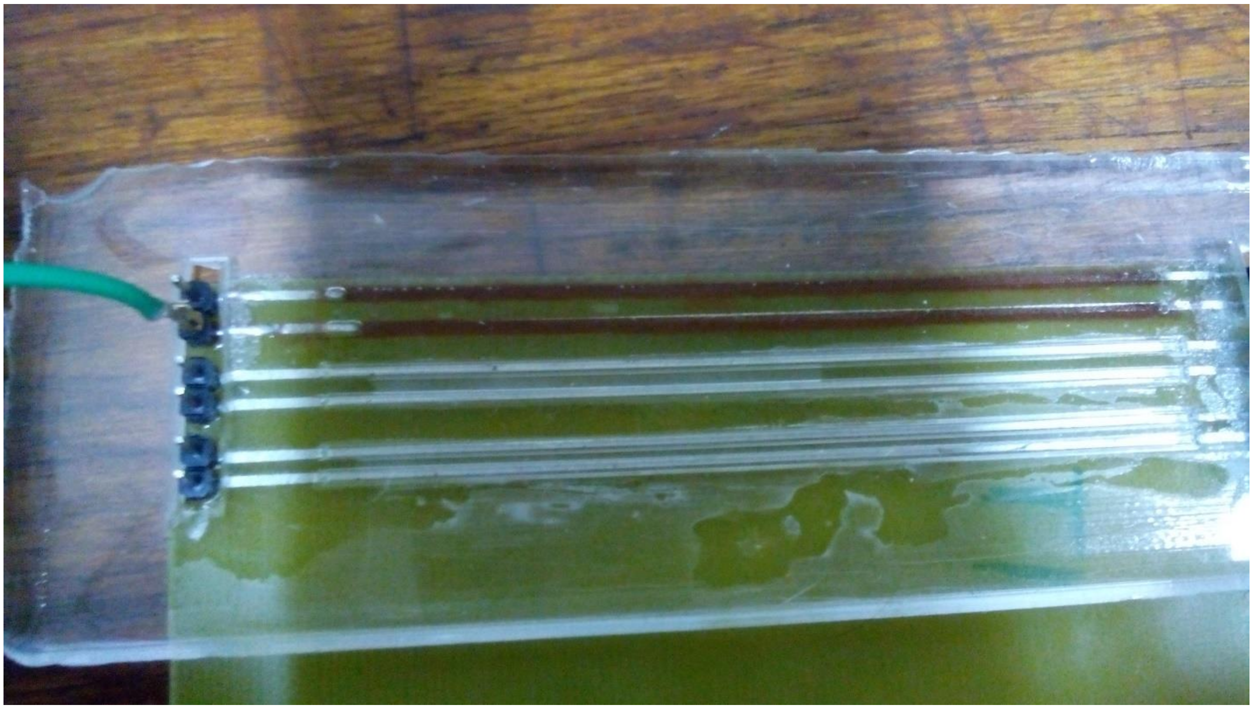

FIG. S84. Copper track with PDMS channel filled up with dispersion

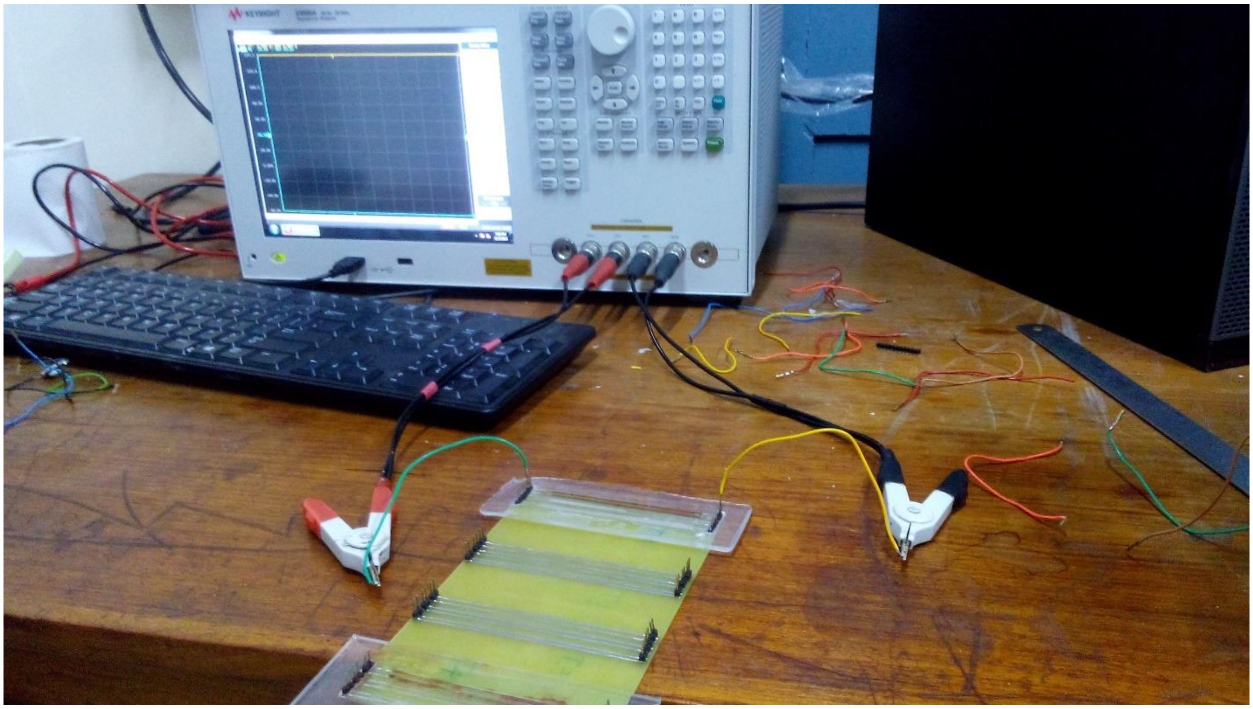

FIG. S85. Experimental Setup.
